# Discovery of a novel allosteric inhibitor scaffold for polyadenosine-diphosphate-ribose polymerase 14 (PARP14) macrodomain 2

**DOI:** 10.1016/j.bmc.2018.03.020

**Published:** 2018-07-15

**Authors:** Moses Moustakim, Kerstin Riedel, Marion Schuller, Andrè P. Gehring, Octovia P. Monteiro, Sarah P. Martin, Oleg Fedorov, Jag Heer, Darren J. Dixon, Jonathan M. Elkins, Stefan Knapp, Franz Bracher, Paul E. Brennan

**Affiliations:** a Structural Genomics Consortium, University of Oxford, ORCRB, Old Road Campus, Headington, Oxford, Oxfordshire OX3 7DQ, UK; b Target Discovery Institute, University of Oxford, NDM Research Building, Old Road Campus, Headington, Oxford, Oxfordshire OX3 7FZ, UK; c Department of Chemistry, Chemistry Research Laboratory, University of Oxford, Oxford OX1 3TA, UK; d Department of Pharmacy - Center for Drug Research, Ludwig-Maximilians University, Munich 81377, Germany; e Charles River, Chesterford Research Park, CB10 1XL, UK; f UCB Pharma Ltd, Slough SL1 3WE, UK; g Structural Genomics Consortium, Universidade Estadual de Campinas, Cidade Universitária Zeferino Vaz, Campinas, SP 13083-886, Brazil; h Johann Wolfgang Goethe-University, Institute for PharmaceuticalChemistry and Buchmann Institute for Life Sciences, Frankfurt am Main 60438, Germany; i German Cancer Centre (DKFZ) and DKTK site Frankfurt/Mainz, 60590, Germany

**Keywords:** PARP, Poly-ADP ribsose, Macrodomain, Inhibitor Design

## Abstract

The polyadenosine-diphosphate-ribose polymerase 14 (PARP14) has been implicated in DNA damage response pathways for homologous recombination. PARP14 contains three (ADP ribose binding) macrodomains (MD) whose exact contribution to overall PARP14 function in pathology remains unclear. A medium throughput screen led to the identification of *N*-(2(-9H-carbazol-1-yl)phenyl)acetamide (GeA-69, **1**) as a novel allosteric PARP14 MD2 (second MD of PARP14) inhibitor. We herein report medicinal chemistry around this novel chemotype to afford a sub-micromolar PARP14 MD2 inhibitor. This chemical series provides a novel starting point for further development of PARP14 chemical probes.

## Graphical abstract

**Figure:**
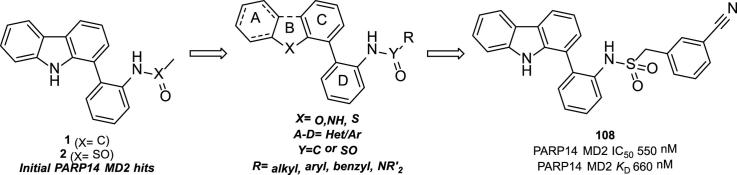

## 1 Introduction

Poly-(ADP ribose) Polymerases (PARPs) are ADP-ribosyl transferase enzymes which post-translationally modify substrate proteins.^1^ Of at least 17 human family members of PARPs a sub-set, referred to as mono(ADP-ribose)transferases (mARTs), are capable of transferring on a single ADP unit to a given substrate.^2^ PARP14 (ARTD8) is the largest of the mARTs and contains multiple domains including an ADP ribose transferase domain (ART), a WWE domain, two (RNA binding) RRM repeats and three (ADP-ribose binding) macrodomains.^3^ PARP14 was found to be highly expressed in B-cell lymphoma and hepatocellular carcinoma and has been associated with poor patient prognosis.^4^ Furthermore PARP14 has been linked to inhibition of pro-apoptotic kinase JNK1 which activates pyruvate kinase M2 isoform (PKM2) which in turn promotes a higher rate of glycolysis in cancer (Warburg effect)^5^ shown in some contexts to be regulated by high MYC expression.^6^ Despite links with cancer pathogenesis^5^, ^7^ and inflammatory diseases,(b), (c), 7, 8 only a few small molecule PARP14 inhibitors have been reported and many have suffered from a lack of selectivity.^9^ Most examples of PARP inhibitors have targeted the catalytic domain (ART)^10^ such as a recent example by Upton and coworkers who identified moderately selective PARP14 inhibitors,10e however to date no PARP14 modulators targeting other domains such as the macrodomains have been reported until recently.^11^

**Figure 1:**
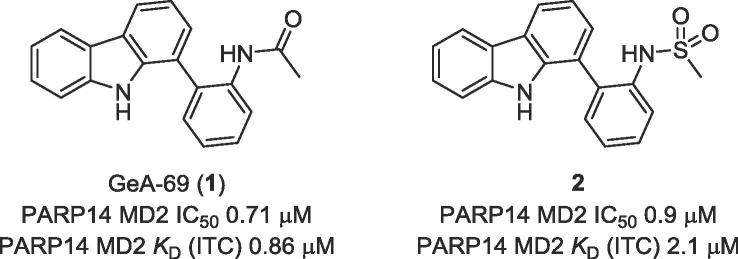
Initial hit PARP14 MD2 inhibitor GeA-69 (**1**) and sulfonamide analogue **2**.

PARP14 contains three macrodomain modules (MD1, MD2 and MD3); biophysical characterisation of macrodomain:ADP-ribsose peptide binding was carried out revealing MD2 as the most potent ADP ribsosyl peptide binding domain and therefore the most likely to deliver a functional effect through small molecule inhibition (PARP14 MD1/ADP-ribose peptide *K*_D_ 137 ± 7 μM, PARP14 MD2/ADP-ribose peptide *K*_D_ 6.8 ± 0.1 μM, PARP14 MD3/ADP-ribose peptide *K*_D_ 15 ± 0.9 μM, Supp. Info Fig. 1 (Fig. 1).

**Figure 2:**
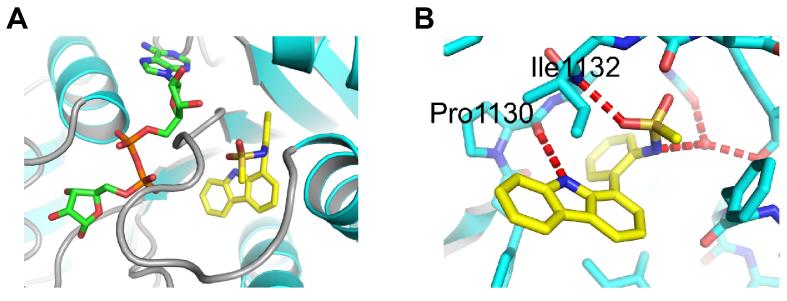
(A) Overlay of bound ADPR (Green sticks) (PDB ID 3Q71) superimposed with PARP14 MD2 (cyan sheets and helices, grey loops): compound **2** (yellow sticks) structure (PDB ID 5O2D). (B) H-Bonding displayed in co-crystal structure of PARP14 MD2 (cyan sticks): compound **2** (yellow sticks) structure (PDB ID 5O2D).

An initial medium throughput screen (∼50 k compounds) revealed compound GeA-69 (**1**) as a sub-micromolar inhibitor of PARP14 MD2 ADP-ribose binding as measured by AlphaScreen™, ITC and BLI.11a A co-crystal structure of closely related sulfonamide derivative **2** with PARP14 MD2, which was obtained in the course of the project revealed a unique allosteric binding mode for this inhibitor (PDB ID 5O2D). Overlay of this structure with bound ADP-ribose from a previously published co-crystal structure of PARP14 MD2 (PDB ID 3Q71)^12^ showed that compound **2** occupied a novel pocket adjacent to the binding site for ADP-ribose (Fig. 2A).11a

Carbazole **2** engages PARP14 MD2 in a pocket adjacent to the ADP-ribose binding site and the interaction is characterised by a H-bond between the carbazole *N-*H and backbone carbonyl of Pro1130 (*N*-O distance 2.8 Å), an H-bond between one sulfonamide carbonyl and the backbone *N-*H of Ile1132 (O-N distance 2.8 Å), and an H-bond from the sulfonamide *N* to a water molecule in the binding pocket. A comparison of the two structures rationalises inhibitory activity as carbazole **2** induces a shift in the loop region adjacent to Pro1130 which consequently moves into the ADP-ribose binding site (Fig. 2A). Evaluation of the co-crystal structure of carbazole **2** with PARP14 MD2 also revealed the possibility of extending the methanesulfonamide motif into larger substituents exploring peripheral regions of this newly identified allosteric site.

## 2 Results

### 2.1 Systematic SAR studies of screening hit GeA-69 (1)

**Figure 3:**
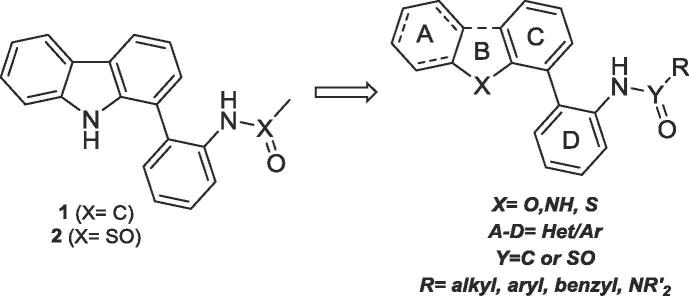
SAR studies of carbazoles GeA-69 (**1**) and **2**.

**Scheme 1:**
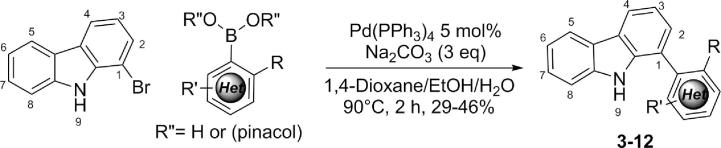
Suzuki-Miyaura coupling of 1-bromo-9(H)-carbazole with arylboronic acids or pinacol esters.

The screening hit GeA-69 (**1**) was part of a focused library from the Bracher lab, originally designed for the improvement of kinase inhibitors derived from the 1-(aminopyrimidyl)-β-carboline alkaloid annomontine.^13^ The SAR studies on screening hit GeA-69 (**1**) are described in the following compound library generated as potential PARP14 MD2 inhibitors (Fig. 3). In this library, the β-carboline ring system was replaced by its deaza analogue carbazole, and a number of aromatic and heteroaromatic rings were attached to position 1 (Scheme 1) using Suzuki-Miyaura cross coupling reactions of known 1-bromocarbazole^14^ with commercially available or synthesised boronic acids and esters to give compounds **3**–**12** (Scheme 1).

**Figure 4:**
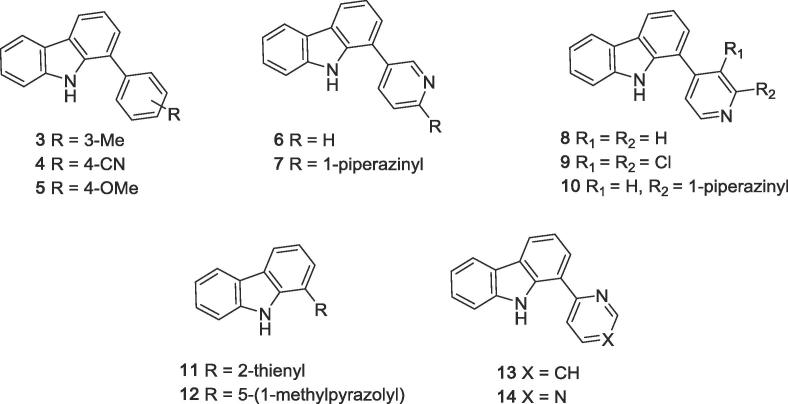
1-Aryl- and 1-heteroarylcarbazoles **3**–**14** from the initial compound library. PARP14 MD2 IC_50_ > 50 µM for all compounds.

2-Pyridyl compound **13** and 4-pyrimidyl analogue **14** were obtained by regioselective nucleophilic addition of 1,9-dilithiated carbazole (obtained in situ from 1-bromocarbazole and 4 equiv. *tert*-butyllithium) to pyridine and pyrimidine, followed by spontaneous rearomatisation during workup. The obtained (hetero)arylcarbazoles are shown in Fig. 4.

Unfortunately none of these analogues (compounds **3**–**14**) showed any inhibition of PARP14 MD2. Only a few further modifications of the 1-aryl substituent were performed, whereby all new compounds contained the acetylamino moeity, which was recognised as important for activity in this early stage of the project.

**Figure 5:**
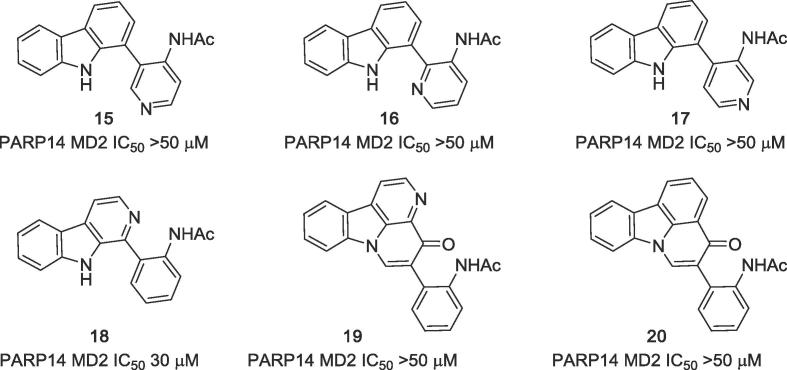
Aza analogues of screening hit GeA-69 (**1**): compounds **15**–**18** and analogues bearing tetracyclic core structures canthin-4-one **19**, desazacanthin-4-one **20**.

The aza analogue **15** was obtained from *N*-SEM protected 1-bromocarbazole by Masuda borylation at C-1, directly followed by Suzuki-Miyaura cross-coupling with 4-amino-3-bromopyridine, subsequent *N*-acetylation and SEM deprotection, as previously described.11a This compound has virtually identical size as the active compound **1**, but interestingly was found to be completely inactive at inhibiting PARP14 MD2 presumably due to the differences in electronics of both molecules. Consequently, this compound could serve as a useful negative control in biochemical experiments. The pyridyl-isomers **16** and **17** were obtained in the same manner using 3-amino-2-chloro- and 3-amino-4-chloropyridine in the cross-coupling reaction (Fig. 5). Furthermore, using Suzuki-Miyaura cross-coupling reactions, the acetylaminophenyl residue was attached to position 1 (Scheme 1) of the β-carboline ring system^15^ in order to obtain a ring A aza-analogue **18** and to the canthin-4-one **19** and desazacanthin-4-one^16^ **20** ring systems in order to give analogues bearing tetracyclic core structures (Fig. 5).

**Figure 6:**
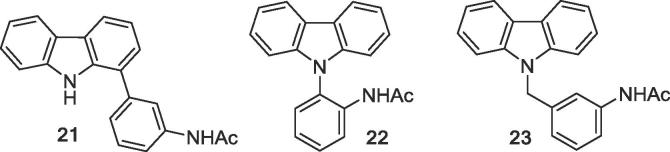
Analogues of GeA-69 (**1**) with the acetylaminophenyl residue shifted to other positions.

An analogue of GeA-69 (**1**) with the acetamido group shifted from the ortho to the meta position at the phenyl ring **21** was prepared by Suzuki-Miyaura cross-coupling of 1-bromocarbazole with 3-aminophenyl boronic acid, followed by *N*-acetylation. Additionally, the complete acetylaminophenyl residue was shifted from *C*-1 to *N*-9, whereby in one example a rigid isomer **22** was obtained, and in the other, by means of a methylene spacer, a product **23** in which by appropriate rotation both the phenyl and the acetamido group can adopt positions that are very similar to those these groups have in the lead structure GeA-69 (**1**). Compound **22** was obtained by *N*-arylation of carbazole with 2-fluoro-1-nitrobenzene,^17^ subsequent reduction of the nitro group, and *N*-acetylation. *N*-Benzyl analogue **23** was prepared in an analogous manner via *N*-alkylation of carbazole with 3-nitrobenzyl chloride (Fig. 6).

**Figure 7:**
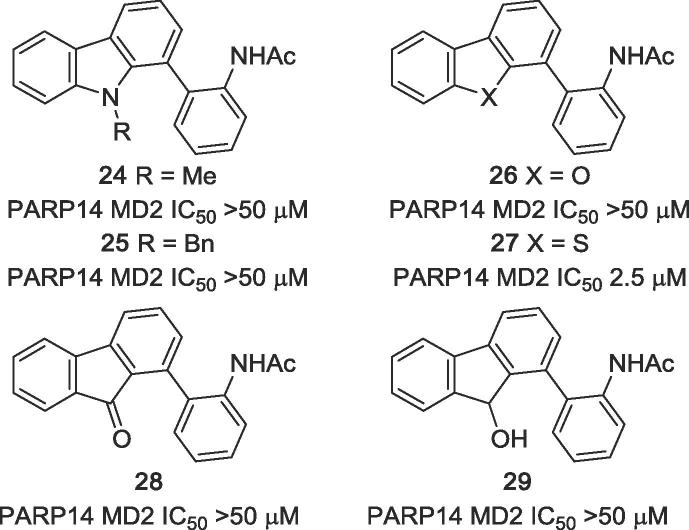
Analogues of GeA-69 (**1**) bearing substituents an *N*-9, as well as dibenzofuran (**26**), dibenzothiophene (**27**), fluorenone (**28**), and fluorenol (**29**) analogues.

As modifications of the central pyrrole ring (ring B) of GeA-69 (**1**) *N*-methyl and *N*-benzyl analogues **24** and **25** were prepared starting from corresponding *N*-substituted 1-bromocarbazoles via Suzuki-Miyaura cross-coupling with 2-aminophenylboronic acid and subsequent *N*-acetylation. Dibenzofuran analogue **26** and dibenzothiophene analogue **27** were obtained in a similar manner from commercially available 4-bromodibenzofuran and known 4-iododibenzothiophene (Fig. 7).^18^ These experiments were performed before we obtained the crystal structure of PARP14 MD2 with inhibitor **2**, which demonstrated the relevance of the pyrrole NH-group (Fig. 2).

In order to replace the NH group of ring B with either an alternative hydrogen bond donor (hydroxy group) or a hydrogen bond acceptor (carbonyl group), known 1-iodofluorenone^19^ was coupled in the established manner to give the 1-arylfluorenone **28** which was easily reduced to the racemic fluorenol **29** with sodium borohydride (Fig. 7).

**Scheme 2:**
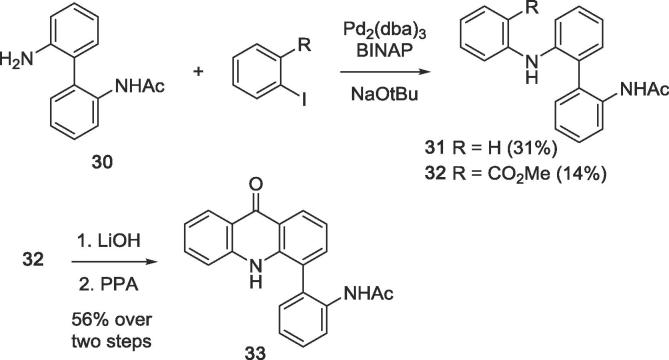
Synthesis of seco analogue **31** and acridone analogue **33**.

Controlled mono-acetylation of 2,2′-diaminobiphenyl with equimolar amounts of acetic anhydride gave monoamide **30** in moderate yield. Monoamide **30** was then used to access the seco analogue **31** and the acridone analogue **33**. Buchwald-Hartwig arylation of the unsubstituted anilino group with iodobenzene to give biaryl **31** and with methyl 2-iodobenzoate to give biaryl **32**, respectively, was accomplished with the BINAP/Pd_2_(dba)_3_ catalyst system. Ester **32** was hydrolysed to give the corresponding carboxylic acid, which was converted into the acridone **33** by polyphosphoric acid-mediated intramolecular acylation (Scheme 2).^20^

**Scheme 3:**
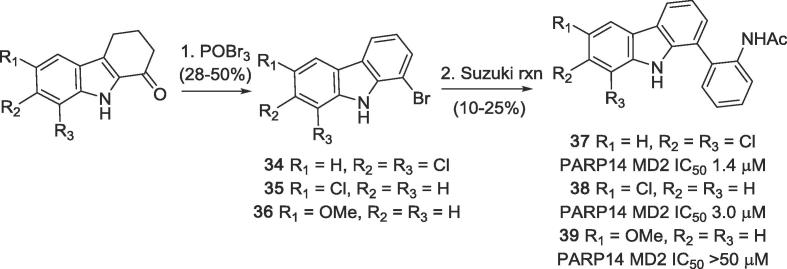
Synthesis of analogues of of GeA-69 (**1**) bearing additional substituents at ring A.

Further, a series of modifications of ring A was performed. Ring-substituted analogues **37**–**39** were obtained in two steps from readily available 1,2,3,4-tetrahydrocarbazol-1-ones^21^ **34**–**36** in two steps. Treatment of the ketones with POBr_3_ in anisole gave the corresponding 1-bromocarbazoles under bromination/dehydrogenation conditions in moderate to poor yields. Subsequent standard Suzuki-Miyaura cross-coupling gave the desired arylcarbazoles **37**–**39** (Scheme 3).

**Scheme 4:**
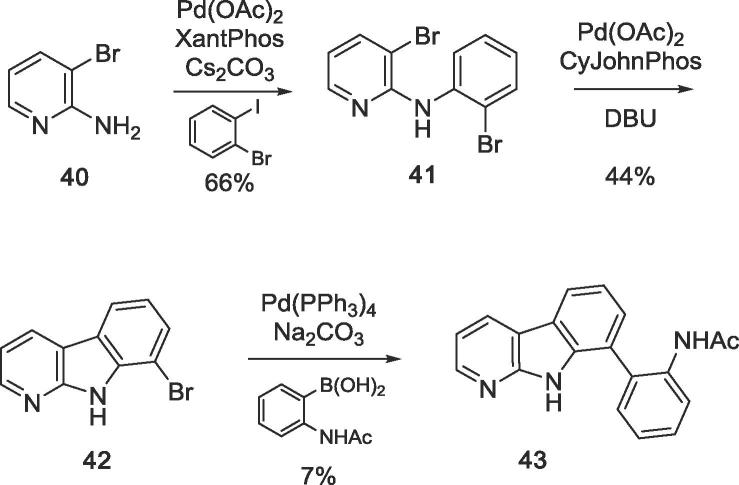
Synthesis of an 8-aza analogue **43** of GeA-69 (**1**).

8-Aza analogue **43** was obtained by a series of three consecutive Pd-catalyzed coupling reactions.^22^ Chemoselective Buchwald-Hartwig amination of 1-bromo-2-iodobenzene with 2-amino-3-bromopyridine **40** using XantPhos as a ligand gave phenylaminopyridine **41**, which was cyclised to 8-bromo-α-carboline **42** using CyJohnPhos in an intramolecular Heck coupling. Finally, the acetylaminophenyl residue was introduced in a standard Suzuki-Miyaura cross-coupling (Scheme 4).

**Scheme 5:**
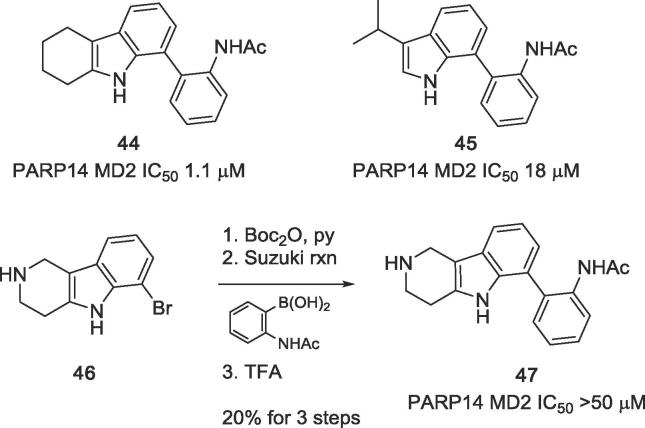
Analogues of GeA-69 (**1**) with partially hydrogenated or truncated ring A.

Analogue **44** bearing a partially hydrogenated A-ring was obtained from the corresponding brominated tetrahydrocarbazole^23^ via Suzuki-Miyaura cross-coupling. A truncated analogue, the 7-aryl-3-isopropylindole **45**, in which ring C is replaced by an isopropyl group, was obtained by Suzuki-Miyaura cross-coupling of the respective 7-bromoindole. The 6-aza-5,6,7,8-tetrahydro analogue **47** was prepared in a similar manner from known intermediate **46**.^24^ Improved yields were obtained, if the secondary amine was protected with the Boc group prior to the cross-coupling reaction (Scheme 5).

**Scheme 6:**
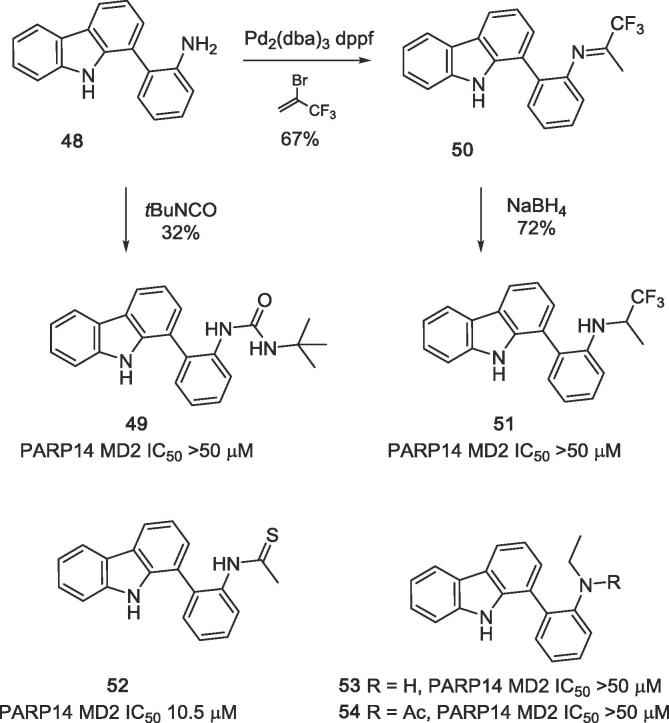
Variations of the acetamide group (thioamide **52**, reduced *N*-ethylamine **53**, *N*-ethyl analogue **54**, urea analogue **49**). Synthesis of the proposed amide bioisoster **51** from aniline **48**.

Finally, modifications of the acetamido group located at the 1-phenyl substituent (ring D) were performed. Aminophenyl intermediate **48** was further converted into the urea analogue **49** by treatment with *tert*-butyl isocyanate (Scheme 6). Since α-trifluoroethylamines are known as bioisosteres of amide groups from peptide chemistry,^25^ we also prepared compound **51** for SAR studies. Intermediate **48** was thus converted into 1,1,1-trifluoropropan-2-imine **50** by Pd-catalysed cross-coupling with 2-bromo-3,3,3-trifluoro-1-propene;^26^ subsequent reduction with sodium borohydride gave the racemic target compound **51**. Treatment of GeA-69 (**1**) with Lawesson’s reagent gave the thioamide analogue **52**. Reduction of the amide group in **1** with borane-disulfide yielded the *N*-ethyl analogue **53**, which in turn could be *N*-acetylated to give the *N*-ethyl acetamide **54**.

A screening of the above presented compounds on PARP14 MD2 clearly demonstrated that lead structure GeA-69 (**1**) is very sensitive to structural modifications. Carbazoles bearing (hetero)aromatic residues different from the acetylaminophenyl residue of GeA-69 (**1**) (Figure 4) were found to be inactive. Analogues with almost identical shape albeit very different electronically (aza analogues in the rings A, C and D) are completely or virtually (β-carboline **18**, IC_50_ 30 μM) inactive. Any changes in the central pyrrole ring (ring B) eliminated inhibitory activity as well. The NH group was found to be essential, it can not be replaced by another hydrogen bond donor, as demonstrated by the inactive fluorenol analogue, **29**. Surprisingly, the dibenzothiophene analogue **27** showed considerable inhibition (IC_50_ 2.5 μM), whereas the dibenzofuran, **26** and the acridone, **33** were inactive. The same holds for the (deaza)compounds having tetracyclic canthin-4-one backbones (canthin-4-one **19**, deazacanthin-4-one **20**). The seco analogue of GeA-69 (**1)**, biaryl **31**, was completely inactive, demonstrating that not only the presence of the functional groups of the lead structure, but also their fixation by the carbazole backbone is most important.

The tetrahydro-analogue **44** showed only a slight loss in activity (IC_50_ 1.1 μM) compared to GeA-69 (**1**), whereas its 6-aza analogue **47** bearing a polar aliphatic amino group in ring A, was inactive. Lipophilic chlorine substituents at ring A (compounds **37**–**38**) were fairly tolerated (IC_50_ 1.4 and 3.0 μM), but the 6-methoxy analogue **39** was inactive. These observations can be rationalised by the hydrophobic environment in the binding region of ring A consisting of residues V1032, V1092, M1108, I111, I1112, F1129, I1132 (Fig. 2).

Removal of the *N*-acetyl residue from GeA-69 (**1**), conversion of the acetamide into a tertiary amide **54** or into the proposed trifluoroalkyl bioisoster **51**, as well as reduction of the amide moiety to an amine **53** resulted in complete loss of activity, the thioamide **52** was an order of magnitude less active (IC_50_ 10.5 μM) than GeA-69 (**1**).

In conclusion, these data confirm a very narrow structure-activity relationship for rings A-C (Fig. 3), and for further optimisation of the screening hit GeA-69 (**1**) only modifications of either the *N*-acyl residue or ring D were deemed promising.

### 2.2 SAR studies of ring D and *N*-acyl residues

Initial construction of the carbazole series was performed using 1-bromo-9*H*-carbazole and a series of pinacol boronic esters which were coupled under standard Suzuki-Miyaura conditions, furnishing biaryl products in moderate to good yields (Scheme 1). A number of these compounds were then converted to the corresponding acetamides or methanesulfonamides and profiled for their binding activity with PARP14 MD2. Whilst binding activity was not improved, additional substituents on ring D such as methyl, fluoro and cyano were tolerated maintaining single digit μM activity (compounds **55**–**57**, Table 1). As previously observed a comparison of these compounds with the inactive non-acetylated and non-sulfonylated anilines (eg compounds **59**–**61**, Table 1) showed the requirement of this group for binding activity.

**Table 1:** Binding affinity characterisation data of carbazole series for PARP14 MD2.

	R	R’	IC_50_ (μM)	*K*_D_ (μM)		R	X	IC_50_ (μM)
**1** (GeA-69)	**-NHAc**	**H**	**0.72 ± 0.04**	**0.86** ± 0.04	**66**	-Et	C	1.0 ± 0.03
**2**	-NHSO_2_Me	H	0.9 ± 0.09	2.1 ± 0.1	**67**	*n*-Pr	C	0.9 ± 0.04
**17**	-NHAc	3-aza	>50	n.d.	**68**	*n*-Bu	C	>50
**49**	-NHC(O)NH-*t*Bu	H	7.2 ± 1.4	n.d.	**69**	-CH_2_CH_2_OMe	SO	8.6 ± 0.4
**52**	-NHC(S)CH_3_	H	10.5 ± 0.4	n.d.	**70**	5-Methylisoxazo-4-yl	SO	>50
**53**	-NHEt	H	>50	n.d.	**71**	-NMe_2_	SO	2.5 ± 0.1
**54**	-NEtAc	H	>50	n.d.	**72**	Ph	SO	>50
**55**	-NHSO_2_Me	4-Me	1.1 ± 0.1	n.d.	**73**	-CF_3_	C	1.1 ± 0.07
**56**	-NHSO_2_Me	4-CN	n.d.^a^	5.2 ± 1.7	**74**	-Cyclopropyl	C	1.2 ± 0.03
**57**	-NHAc	6-Me	1.7 ± 0.1	1.6 ± 0.7	**75**	-Cyclohexyl	C	>50
**58**	-NHSO_2_Me	5-CF_3_	>50	n.d.	**76**	-2-furyl	C	>50
**59**	-NH_2_	4-Me	>50	n.d.	**77**	-Ph	C	1.9 ± 0.07
**60**	-NH_2_	5-CF_3_	>50	n.d.	**78**	**-CH_2_Ph**	**C**	**7.6 ± 0.3**
**61**	-NH_2_	6-Me	>50	n.d.	**79**	**-CH_2_Ph**	**SO**	**3.6 ± 0.3**
**62**	-NHSO_2_Et	H	1.2 ± 0.03	n.d.	**80**	2-OMe-Ph	C	>50
**63**	-NHSO_2_*n*-Pr	H	2.9 ± 0.1	n.d.	**81**	3-OMe-Ph	C	12.7 ± 1.5
**64**	-NHSO_2_*n*-Bu	H	3.3 ± 0.1	n.d.	**82**	4-OMe-Ph	C	9.0 ± 1.4
**65**	-NHC(O)CH_2_NMe_2_	H	5.5 ± 0.6	n.d.				

a Data was not successfully obtained due to solubility issues in the AlphaScreen assay with this example.

**Scheme 7:**
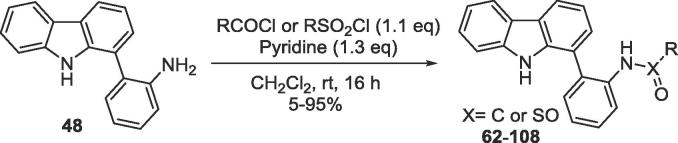
Synthesis of amide and sulfonamide derivatives of aniline **48**.

Further modification of biaryl-amine **48** to the corresponding amides or sulfonamides (Scheme 7) was carried out. The corresponding amides and sulfonamides **62**–**108** were then profiled for their PARP14 MD2 binding affinity (Tables 1 and 2).

**Table 2:** Binding affinity characterisation data of carbazole series for PARP14 MD2.

	R/Het	X	IC_50_ (μM)^a^	*K*_D_ (μM)		R/Het	X	IC_50_ (μM)^a^
**83**	3-aza	C	1.1	1.5^a^	**96**	4-Cl	C	6.2 ± 0.6
**84**	3-aza-4-Me	C	1.0	2.7 a	**97**	4-CF_3_	SO	8.1 ± 0.7
**85**	3, 6-aza	C	2.1 ± 0.1	3.9 a	**98**	3,4-OMe	C	4.3 ± 0.3
**86**	4-aza-3-CN	C	2.4 ± 0.2	n.d.	**99**	3,4-dioxole	C	6.9 ± 0.6
**87**	3-aza-4-CN	C	3.5 ± 0.2	n.d.	**100**	2-F-5-CN	SO	1.2 ± 0.0 (*K*_D_ 1.3 ± 0.51)
**88**	3-aza-4-OH	C	6.6 ± 0.4	n.d.	**101**	2,5-Me	C	44.6 ± 10.4
**89**	2-F	SO	2.4 ± 0.1	n.d.	**102**	3,4-Cl	C	6.3 ± 0.6
**90**	2-F	C	2.8 ± 0.2	n.d.	**103**	3-OMe	C	6.6 ± 0.5
**91**	2-Cl	C	4.4 ± 0.3	n.d.	**104**	3-F	C	4.2 ± 0.3
**92**	4-Me	C	8.7 ± 1.2	n.d.	**105**	3-F	SO	1.4 ± 0.1
**93**	4-F	C	>50	n.d.	**106**	3-CF_3_	C	7.1 ± 0.7
**94**	4-OMe	C	6.2 ± 0.6	n.d.	**107**	3-CN	C	2.1 ± 0.1
**95**	4-CN	SO	6.2 ± 0.6	n.d.	**108**	**3-CN**	**SO**	**0.66 ± 0.03** **(*K*_D_ 0.55 ± 0.22)**

a No error of fit obtained for these *K*_D_ values. n.d. denotes not determined.

Compounds were profiled for binding activity with PARP14 MD2 through a competitive (AlphaScreen™) binding assay measuring the displacement of ADP-ribose peptide from PARP14 MD2.11a Promising compounds were additionally profiled by biophysical assays such as Bio-Layer Interferometry or Isothermal Titration Calorimetry as previously described.11a

As previously described the parent carbazole GeA-69 (**1**) was profiled for its broader selectivity over 12 other human macrodomains, showing exquisite selectivity for MD2 of PARP14.11a Furthermore a representative selectivity screen of 46 kinases in a Differential Scanning Calorimetry assay did not reveal any significant activity of carbazole GeA-69 (**1**) at 10 μM.11a

## 3 Discussion

**Figure 8:**
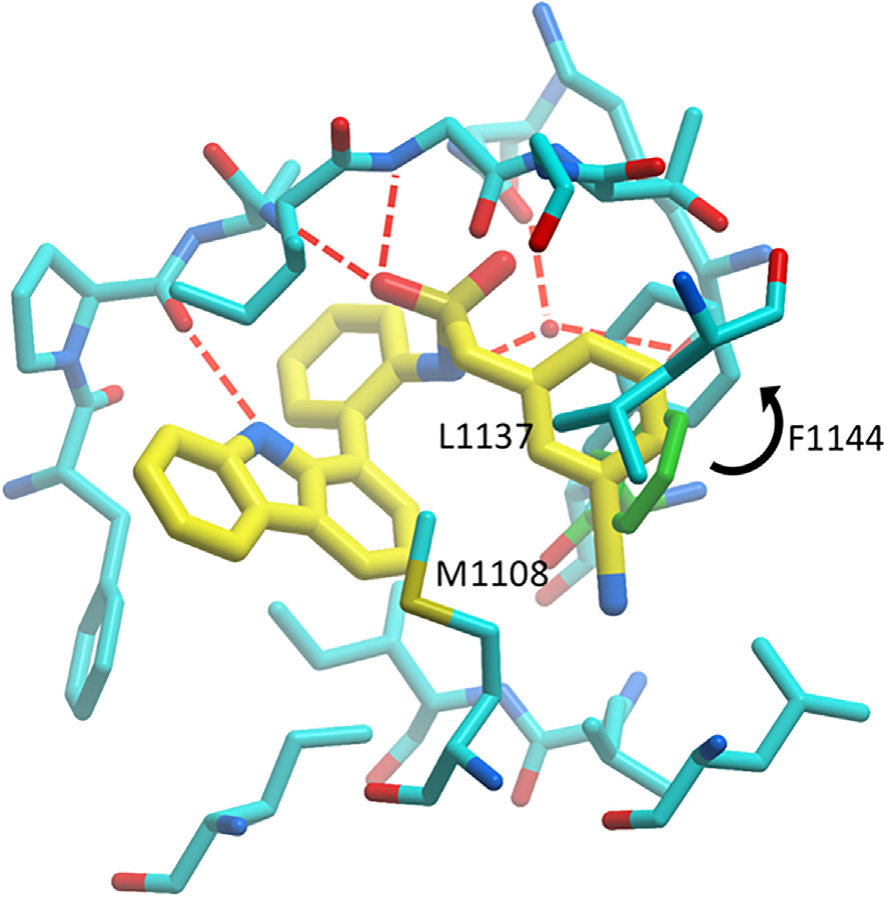
Flexible side-chain docking studies of carbazole**108** with PARP14 MD2 (from PDB ID 5O2D) reveal new potential hydrophobic interactions with M1108, L1137 and F1144 after rotation of F1144 (black arrow, conformation in PDB ID 5O2D shown in green sticks) to accommodate the 3-cyanophenyl group.

The binding activities of synthesised PARP14 MD2 inhibitors are summarised in Tables 1 and 2. Despite comprehensive SAR studies of the A-C rings of this carbazole series, no points for the development of more potent ligands were discovered, a number of derivatives were synthesised functionalising ring D (Figure 3). Only small additional substituents to the ring were tolerated (e.g. compounds **55**–**57**, Table 1). Interestingly, elaboration of the sulfonamide in compound **2** into the homologated ethane-, propane- and butane-sulfonamides analogues (compounds **62**–**64**, Table 1) furnished equipotent compounds. Further elaboration of the acetamide in GeA-69 (**1**) mostly retained single digit μM binding activity (eg compounds **66**,**67**). Interestingly the *n*-pentanoyl analogue **68** was seemingly inactive, which may be due the entropic penalty associated with longer alkyl substituents or a steric clash with the protein. However, guided by the apparent tolerance of some larger substituents in place of the acetamide in GeA-69 (**1**) and methanesulfonamide in compound **2**, the 2-phenylacetamide and phenylmethanesulfonamide of compounds **78** and **79** (IC_50_ 7.6 ± 0.3 and 3.6 ± 0.3 μM respectively, Table 1) were chosen for further development as they enabled rapid access to diversity and provide a suitable vector for binding pocket exploration. A number of hetero- and substituted- aromatics were appended onto the biaryl core (examples **83**–**108**, Table 2). Moderately flat SAR was observed for both 2- and 4- substituted phenylacetyl and phenylmethanesulfonamide groups. It was found that introduction of a 3-cyano substituent in the phenylmethanesulfonamide series provided a slight improvement in binding activity compared with GeA-69 (**1**). Carbazole **108** displays sub-micromolar activity for PARP14 MD2 (IC_50_ 660 ± 30 nM). Notably, by comparison the corresponding 3-cyanophenylacetamide **107** displays diminished binding activity relative to sulfonamide **108**, potentially due to the greater tolerance of the sulfonamide to maintaining H-bond acceptor interactions as shown in the PARP14 MD2:compound **2** co-crystal structure (Fig. 2B). The 3-cyanobenzyl group of compound **108** may make interactions with adjacent hydrophobic residues M1108, L1137 and F1144. Although we were unable to obtain a co-crystal structure of compound **108** to confirm these interactions, we performed docking studies to examine possible binding modes of the larger compound compared to compound **2**. Simple minimisation of compound **108** in PARP14 MD2 is unable to find a binding pose due to clashes between the larger 3-cyanophenyl group and the protein. To account for potential side chain rotations that would be necessary to accommodate this group, we performed SCARE docking (SCan Alanines and Refine) using ICM.^27^ The optimised pose for compound **108** shows a rotation of the side chain of F1144 to open up space so that the 3-cyanophenyl group can make interactions with M1108 and L1137 in addition to a pi-stacking interaction with F1144 (Fig. 8). However, it is not obvious from this docking study why the 3-cyanophenyl group would be preferred to other hydrophobic groups such as in compounds **79** and **83**–**107**.

Sub-micromolar PARP14 MD2 affinity of carbazole **108** was also confirmed by BioLayer Interferometry (BLI) providing a calculated *K*_D_ of 550 nM ± 220. Whilst lead compound **108** is larger and a less ligand efficient inhibitor of PARP14 MD2 than original hit compound **1**, owing to the more tolerant SAR around it represents an attractive chemical starting point for future development. Additional examples similar to compound **108** (see SI, compounds **109**–**116**) have been explored and work to improve the binding activity and physicochemical properties of this lead molecule will be reported in due course.

## 4 Summary

We herein report the development of a novel class of allosteric modulators of the second macrodomain of PARP14. Initial identification of carbazole GeA-69 (**1**) as a submicromolar inhibitor of PARP14 MD2 was made following a medium throughput screen.11a Inhibitory activity can be rationalised through a PARP14 MD2 co-crystal of a similar derivative, sulfonamide **2** (PDB ID 5O2D). Investigation into this carbazole series was then made revealing new opportunities for ligand elaboration. Systematic analysis of SAR demonstrated a very narrow structure activity relationship for rings A-C (carbazole scaffold), and for further optimisation of the screening hit **1** only modifications of either the *N*-acyl residue or ring D showed promise. A number of carbazole containing compounds were tolerated in this newly identified allosteric site of PARP14 MD2 including a 3–cyano substituted phenylmethanesulfonamide **108**. Carbazole **108** displays submicromolar activity binding to PARP14 MD2 by AlphaScreen (IC_50_ 0.66 μM) which was also confirmed by BLI (*K*_D_ 0.55 μM). This lead molecule along with others in this series are useful chemical starting points in the development of chemical probes for this poorly understood epigenetic target.
