# Supplementary material for: Discovery of a novel allosteric inhibitor scaffold for polyadenosine-diphosphate-ribose polymerase 14 (PARP14) macrodomain 2
Source: Bioorg Med Chem. 2018 Jul 15;26(11):2965–72. doi: 10.1016/j.bmc.2018.03.020 (PMC6008491; doi:10.1016/j.bmc.2018.03.020)
Supplement: Supplementary data 2 [file mmc2.docx]

**Chemistry**

***N*-(2-(9*H*-carbazol-1-yl)phenyl)acetamide GeA-69 (1)**

A degassed stirred solution of 1-bromo-9H-carbazole (200 mg, 0.813 mmol, 1 eq), *N*-(2-(4,4,5,5-tetramethyl-1,3,2-dioxaborolan-2-yl)phenyl)acetamide (212 mg, 0.813 mmol, 1 eq), Na_2_CO_3_ (258 mg, 2.44 mmol, 3 eq) and Pd(PPh_3_)_4_ (94 mg, 0.081 mmol, 0.1 eq) in a mixture of 1,4-Dioxane:EtOH:H2O (2 mL, 2:1:1, 0.4 M) under an inert N_2_ atmosphere was heated with microwave irradiation at 90°C for 2 h. Following completion the crude mixture was concentrated onto silica gel and purified by Isolera Biotage LPLC (CH/EA 8:2) to give **1** (85 mg, 35 %) as an off-white solid.

**v_max_ (cm^-1^)** 3397, 3309, 1686, 1574, 1516, 1453, 1442, 1287, 1238, 736; **^1^H-NMR (400MHz, CDCl_3_): δ_H_** 8.3 (d, J=8.19 Hz, 1 H), 8.2 (m, 3 H), 7.4 (m, 6 H), 7.3 (m, 2 H), 7.1 (br. s., 1 H), 1.8 (s, 3 H); **^13^C-NMR (100 MHz CDCl_3_): δ_C_** 169.0, 139.6, 137.7, 135.4, 130.6, 129.0, 128.8, 126.5, 126.4, 124.9, 23.8, 123.2, 122.6, 120.4, 120.4 ,119.8, 110.9, 24.4; **LR-ESI-MS**: C_20_H_17_N_2_O [M+H]^+^ *m/z* found 301.10, cald 301.13.

**Systematic modifications of screening hit 1**

**First generation 1-(hetero)arylcarbazoles (3-12) prepared by Suzuki-Miyaura cross-coupling of 1-bromocarbazole with (hetero)arylboronic acids**

**General procedure:**

100 mg (0.406 mmol) 1-bromo-9*H*-carbazole, 1.1 equivalents of the boronic acid, and 47 mg (0.046 mmol) Pd(PPh_3_)_4_ were placed in a microwave vial under N_2_ atmosphere. Then a degassed mixture of 3 mL 1,2-dimethoxyethane and 2 mL ethanol was added through the septum, followed by 1 mL of a nitrogen-purged 2 M sodium carbonate solution. The reaction was performed at 70 °C under microwave irradiation (10 min, 150 W). After cooling 50 mL water was added, followed by extraction with diethyl ether (3 x 40 mL). The combined organic layers were dried over Na_2_SO_4_ filtered and evaporated. The residue was purified by silica gel chromatography using hexane-methanol mixtures.

**1-(3-Methylphenyl)-9*H*-carbazole (3)**

**Yield:** 41 mg (39 %), white solid, **Mpt** 59-61 °C. **^1^H-NMR (500 MHz, CD_2_Cl_2_):** δ (ppm) = 8.46 (br s, 1H, NH), 8.11 (d, *J* = 7.8 Hz, 1H, 5-H), 8.07 (d, *J* = 7.7 Hz, 1H, 4-H), 7.54 – 7.49 (m, 2H, 2´-H, 6´-H), 7.47 – 7.40 (m, 4H, 2-H, 7-H, 8-H, 5´-H), 7.32 (t, *J* = 7.5 Hz, 1H, 3-H), 7.29 – 7.23 (m, 2H, 6-H, 4´-H), 2.48 (s, 3H, 3´-CH_3_). **^13^C-NMR (100 MHz, CD_2_Cl_2_):** δ (ppm) = 139.9 (C-8a), 139.5 (C-3´), 139.3 (C-1´), 137.6 (C-9a), 129.5 (C-5´), 129.4 (C-2´), 128.7 (C-4´), 126.3 (C-7), 126.0 (C-2), 125.7 (C-6´), 125.6 (C-1),123.9 (C-4a), 123.8 (C-4b), 120.7 (C-5), 120.2 (C-3), 119.9 (C-6), 119.7 (C-4), 111.1 (C-8), 21.7 (C-3´-CH_3_). **MS (EI):** *m/z* (rel. int. in %) = 257 [M^+^**^•^**] (100), 241 (20), 127 (15). **HR-MS (EI):** *m/z* found 257.1208, calcd. for C_19_H_15_N: 257.1204.

**4-(9*H*-Carbazol-1-yl)benzonitrile (4)**

**Yield:** 85 mg (78 %), white solid, **Mpt:** 258-260 °C. **^1^H-NMR (400 MHz, CD_2_Cl_2_):** δ (ppm) = 8.43 (br s, 1H, NH), 8.15 (d, *J* = 7.6 Hz, 1H, 4-H), 8.13 (d, *J* = 6.9 Hz, 1H, 5-H), 7.89 – 7.83 (m, 4H, 2´-H, 3´-H, 5´-H, 6´-H), 7.50 – 7.44 (m, 3H, 2-H, 7-H, 8-H), 7.36 (t, *J* = 7.6 Hz, 1H, 3-H), 7.28 (ddd, *J* = 7.9 Hz, 6.7 Hz, 1.5 Hz, 1H, 6-H). **^13^C-NMR (100 MHz, CD_2_Cl_2_):** δ (ppm) = 144.2 (C-4´), 140.0 (C-8a), 137.3 (C-9a), 133.6 (C-2´, C-6´), 129.4 (C-3´, C-5´), 126.7 (C-7), 126.3 (C-2), 124.5 (C-4a), 123.6 (C-1, C-4b), 121.0 (C-5), 120.8 (C-4), 120.5 (C-3), 120.3 (C-6), 119.2 (CN), 111.6 (C-1´), 111.3 (C-8). **MS (EI):** *m/z* (rel. int. in %) = 268 [M^+^**^•^**] (100), 241 (5), 121 (5). **HR-MS (EI):** *m/z* found 268.1007, calcd. for C_19_H_12_N_2_: 268.1001.

**1-(4-Methoxyphenyl)-9*H*-carbazole (5)**

**Yield:** 36 mg (32 %), pale yellow solid, **Mpt:** 128-130 °C. **^1^H-NMR (400 MHz, CD_2_Cl_2_):** δ (ppm) = 8.43 (br s, 1H, NH), 8.10 (d, *J* = 7.8 Hz, 1H, 5-H), 8.04 (d, *J* = 7.6 Hz, 1H, 4-H), 7.63 (d, *J* = 8.7 Hz, 2H, 2´-H, 6´-H), 7.45 – 7.38 (m, 3H, 2-H, 7-H, 8-H), 7.30 (t, *J* = 7.6 Hz, 1H, 3-H), 7.24 (ddd, *J* = 7.9 Hz, 6.6 Hz, 1.6 Hz, 1H, 6-H), 7.09 (d, *J* = 8.7 Hz, 2H, 3´-H, 5´-H), 3.88 (s, 3H, CH_3_). **^13^C-NMR (100 MHz, CD_2_Cl_2_):** δ (ppm) = 159.6 (C-4´), 139.9 (C-8a), 137.7 (C-9a), 131.6 (C-1´), 129.8 (C-2´, C-6´), 126.3 (C-7), 125.9 (C-2), 125.2 (C-1), 123.8 (C-4a, C-4b), 120.7 (C-5), 120.3 (C-3), 119.8 (C-6), 119.3 (C-4), 115.0 (C-3´, C-5´), 111.1 (C-8), 54.1 (CH_3_). **MS (EI):** *m/z* (rel. int. in %) = 273 [M^+^**^•^**] (100), 268 (30), 229 (15), 137 (10), 114 (10). **HR-MS (EI):** *m/z* found 273.1158, calcd. for C_19_H_15_NO: 273.1154.

**1-(Pyridin-3-yl)-9*H*-carbazole (6)**

**Yield:** 25 mg (25 %), pale yellow solid, **Mpt:** 182-184 °C. **^1^H-NMR (400 MHz, CDCl_3_):** δ (ppm) = 9.63 (br s, 1H, NH), 9.07 (d, *J* = 1.6 Hz, 1H, 2´-H), 8.55 (dd, *J* = 4.8 Hz, 1.4 Hz, 1H, 6´-H), 8.13 (d, *J* = 7.4 Hz, 1H, 4-H), 8.12 (d, *J* = 7.4 Hz, 1H, 5-H), 7.96 (dt, *J* = 7.8 Hz, 1.8 Hz, 1H, 4´-H), 7.46 – 7.38 (m, 4H, 2-H, 7-H, 8-H, 5´-H), 7.34 (t, *J* = 7.5 Hz, 1H, 3-H), 7.25 (ddd, *J* = 7.8 Hz, 6.7 Hz, 1.5 Hz, 1H, 6-H). **^13^C-NMR (100 MHz, CDCl_3_):** δ (ppm) = 149.5 (C-2´), 148.5 (C-6´), 140.2 (C-8a), 137.8 (C-9a), 136.3 (C-4´), 135.4 (C-3´), 126.4 (C-7), 126.1 (C-2), 124.3 (C-4a, C-5´), 123.5 (C-4b), 121.5 (C-1), 120.7 (C-4, C-5), 120.0 (C-3), 119.8 (C-6), 111.2 (C-8). **HR-MS (EI):** *m/z* found 244.1009, calcd. for C_17_H_12_N_2_: 244.1001.

**1-[6-(Piperazin-1-yl)pyridin-3-yl]-9*H*-carbazole (7)**

**Yield:** 70 mg (52 %), white solid, **Mpt:** 253-256 °C. **^1^H-NMR (500 MHz, DMSO-d_6_):** δ (ppm) = 11.16 (br s, 1H, 9-NH), 8.48 (d, *J* = 2.3 Hz, 1H, 2´-H), 8.13 (d, *J* = 7.8 Hz, 1H, 5-H), 8.08 (d, *J* = 7.6 Hz, 1H, 4-H), 7.84 (dd, *J* = 8.8 Hz, 2.4 Hz, 1H, 4´-H), 7.53 (d, *J* = 7.9 Hz, 1H, 8-H), 7.37 (t, *J* = 7.7 Hz, 1H, 7-H), 7.34 (d, *J* = 7.5 Hz, 1H, 2-H), 7.23 (t, *J* = 7.6 Hz, 1H, 3-H), 7.16 (t, *J* = 7.5 Hz, 1H, 6-H), 6.98 (d, *J* = 8.8 Hz, 1H, 5´-H), 3.51 (t, *J* = 4.4 Hz, 4H, 2´´-H, 6´´-H), 3.17 (br s, 1H, 4´´-NH), 2.82 (t, *J* = 4.4 Hz, 4H, 3´´-H, 5´´-H). **^13^C-NMR (100 MHz, DMSO-d_6_):** δ (ppm) = 158.5 (C-6´), 146.8 (C-2´), 140.2 (C-8a), 137.6 (C-4´), 137.1 (C-9a), 125.6 (C-2), 125.1 (C-7), 123.2 (C-4a, C-3´), 122.5 (C-4b), 122.1 (C-1), 120.1 (C-5), 119.2 (C-3), 118.9 (C-4), 118.7 (C-6), 111.6 (C-8), 107.0 (C-5´), 45.8 (C-2´´, C-6´´), 45.5 (C-3´´, C-5´´). **MS (EI):** *m/z* (rel. int. in %) = 328 [M^+^**^•^**] (65), 298 (20), 286 (65), 272 (75), 259 (100), 243 (35), 217 (10), 164 (10), 143 (20), 122 (10), 56 (20). **HR-MS (EI):** *m/z* found 328.1689, calcd. for C_21_H_20_N_4_: 328.1688.

**1-(Pyridin-4-yl)-9*H*-carbazole (8)**

**Yield:** 48 mg (49 %), pale yellow solid, **Mpt:** 269-271 °C. **^1^H-NMR (500 MHz, DMSO-d_6_):** δ (ppm) = 11.28 (br s, 1H, NH), 8.75 (dd, *J* = 4.6 Hz, 1.4 Hz, 2H, 2´-H), 8.22 (d, *J* = 7.7 Hz, 1H, 4-H), 8.17 (d, *J* = 7.8 Hz, 1H, 5-H), 7.75 (dd, *J* = 4.5 Hz, 1.5 Hz, 2H, 3´-H), 7.56 (d, *J* = 8.0 Hz, 1H, 8-H), 7.51 (d, *J* = 7.4 Hz, 1H, 2-H), 7.42 (t, *J* = 7.6 Hz, 1H, 7-H), 7.31 (t, *J* = 7.6 Hz, 1H, 3-H), 7.20 (t, *J* = 7.4 Hz, 1H, 6-H). **^13^C-NMR (100 MHz, DMSO-d_6_):** δ (ppm) = 150.2 (C-2´), 146.0 (C-4´), 140.3 (C-8a), 136.7 (C-9a), 126.0 (C-7), 125.7 (C-2), 123.7 (C-4a), 123.3 (C-3´), 122.3 (C-4b), 121.8 (C-1), 121.0 (C-4), 120.3 (C-5), 119.3 (C-3), 119.1 (C-6), 111.6 (C-8). **MS (EI):** *m/z* (rel. int. in %) = 244 [M^+^**^•^**] (100), 217 (10), 122 (10), 108 (10), 96 (10). **HR-MS (EI):** *m/z* found 244.0975, calcd. for C_17_H_12_N_2_: 244.1001.

**1-(2,3-Dichlorpyridin-4-yl)-9*H*-carbazole (9)**

**Yield:** 10 mg (8 %), light brown solid, **Mpt:** 235-237 °C. **^1^H-NMR (400 MHz, C_2_D_2_Cl_4_):** δ (ppm) = 8.39 (d, *J* = 4.9 Hz, 1H, 6´-H), 8.24 – 8.18 (m, 2H, NH, 4-H), 8.13 (d, *J* = 7.7 Hz, 1H, 5-H), 7.51 – 7.45 (m, 2H, 7-H, 8-H), 7.43 (d, *J* = 4.8 Hz, 1H, 5´-H), 7.39 – 7.35 (m, 2H, 2-H, 3-H), 7.33 – 7.26 (m, 1H, 6-H). **^13^C-NMR (100 MHz, C_2_D_2_Cl_4_):** δ (ppm) = 150.5 (C-4´), 148.4 (C-2´), 146.8 (C-6´), 139.3 (C-8a), 136.3 (C-9a), 129.6 (C-3´), 126.5 (C-7), 126.1 (C-2), 125.3 (C-5´), 123.6 (C-4a), 122.7 (C-4b), 121.4 (C-4), 120.4 (C-5), 120.0 (C-6), 119.5 (C-3), 119.4 (C-1), 111.0 (C-8). **MS (EI):** *m/z* (rel. int. in %) = 316 (15), 314 [M^+^**^•^**] (70), 312 (100), 277 (75), 262 (20), 241 (75), 214 (35), 199 (20), 183 (35), 149 (35), 138 (40), 125 (20), 121 (50), 108 (70), 94 (55), 77 (40), 71 (35), 63 (30), 57 (50), 51 (55). **HR-MS (EI):** *m/z* found 312.0226, calcd. for C_17_H_10_Cl_2_N_2_: 312.0221.

|  |  |
| --- | --- |

**1-[2-(Piperazin-1-yl)pyridin-4-yl]-9*H*-carbazole (10)**

**Yield:** 225 mg (84 %), white solid, **Mpt:** 202-204 °C. **^1^H-NMR (500 MHz, CDCl_3_):** δ (ppm) = 8.63 (br s, 1H, 9-NH), 8.33 (d, *J* = 5.1 Hz, 1H, 6´-H), 8.11 (d, *J* = 7.7 Hz, 2H, 4-H, 5-H), 7.46 – 7.40 (m, 3H, 2-H, 7-H, 8-H), 7.31 (t, *J* = 7.6 Hz, 1H, 3-H), 7.26 (td, *J* = 7.8 Hz, 2.3 Hz, 1H, 6-H), 6.95 (d, *J* = 5.1 Hz, 1H, 5´-H), 6.89 (s, 1H, 3´-H), 3.62 – 3.50 (m, 4H, 2´´-H, 6´´-H), 3.03 – 2.90 (m, 4H, 3´´-H, 5´´-H), 1.66 (br s, 1H, 4´´-NH). **^13^C-NMR (100 MHz, CDCl_3_):** δ (ppm) = 160.5 (C-2´), 148.7 (C-6´), 148.4 (C-4´), 139.5 (C-8a), 137.0 (C-9a), 126.2 (C-7), 125.3 (C-2), 123.9 (C-4a), 123.4 (C-1), 123.3 (C-4b), 120.5 (C-4, C-5), 119.8 (C-3), 119.7 (C-6), 113.0 (C-5´), 110.8 (C-8), 106.4 (C-3´), 46.4 (C-2´´, C-6´´), 45.9 (C-3´´, C-5´´). **MS (EI):** *m/z* (rel. int. in %) = 328 [M^+^**^•^**] (45), 286 (45), 298 (20), 272 (100), 260 (65), 243 (40), 216 (10), 143 (15), 122 (10), 108 (10), 56 (20). **HR-MS (EI):** *m/z* found 328.1691, calcd. for C_21_H_20_N_4_: 328.1688.

**1-(Thiophen-2-yl)-9*H*-carbazole (11)**

**Yield:** 82 mg (81 %), pale yellow solid, **Mpt** 121-123 °C. **^1^H-NMR (400 MHz, CD_2_Cl_2_):** δ (ppm) = 8.66 (br s, 1H, NH), 8.10 (d, *J* = 7.8 Hz, 1H, 5-H), 8.06 (d, *J* = 7.8 Hz, 1H, 4-H), 7.57 (dd, *J* = 7.5 Hz, 1.0 Hz, 1H, 2-H), 7.50 (d, *J* = 8.1 Hz, 1H, 8-H), 7.48 – 7.41 (m, 3H, 7-H, 4´-H, 5´-H), 7.28 (t, *J* = 7.6 Hz, 1H, 3-H), 7.27 – 7.23 (m, 2H, 6-H, 3´-H). **^13^C-NMR (100 MHz, CD_2_Cl_2_):** δ (ppm) = 141.3 (C-2´), 140.0 (C-8a), 137.2 (C-9a), 128.5 (C-3´), 126.6 (C-7), 125.9 (C-5´), 125.6 (C-2), 125.3 (C-4´), 124.4 (C-4a), 123.7 (C-4b), 120.8 (C-5), 120.3 (C-4), 120.2 (C-3, C-6), 118.3 (C-1), 111.3 (C-8). **MS (EI):** *m/z* (rel. int. in %) = 249 [M^+^**^•^**] (100), 204 (65), 125 (10), 102 (10), 57 (10). **HR-MS EI):** *m/z* found 249.0606, calcd. for C_16_H_11_NS: 249.0612.

**1-(1-Methyl-1*H*-pyrazol-5-yl)-9*H*-carbazole (12)**

**Yield:** 90 mg (88 %), pale yellow solid, **Mpt:** 219-221 °C. **^1^H-NMR (400 MHz, CD_2_Cl_2_):** δ (ppm) = 9.14 (br s, 1H, NH), 8.16 (d, *J* = 7.7 Hz, 1H, 4-H), 8.13 (d, *J* = 7.9 Hz, 1H, 5-H), 7.60 (d, *J* = 1.9 Hz, 1H, 3´-H), 7.52 (d, *J* = 8.1 Hz, 1H, 8-H), 7.45 (ddd, *J* = 8.1 Hz, 7.0 Hz, 1.1 Hz, 1H, 7-H), 7.39 (dd, *J* = 7.4 Hz, 1.2 Hz, 1H, 2-H), 7.33 (t, *J* = 7.5 Hz, 1H, 3-H), 7.26 (ddd, *J* = 7.8 Hz, 6.9 Hz, 1.1 Hz, 1H, 6-H), 6.51 (d, *J* = 1.9 Hz, 1H, 4´-H), 3.76 (s, 3H, CH_3_). **^13^C-NMR (125 MHz, CD_2_Cl_2_):** δ (ppm) = 140.2 (C-8a, C-5´), 139.2 (C-3´), 138.7 (C-9a), 126.9 (C-2), 126.6 (C-7), 124.0 (C-4a), 123.5 (C-4b), 121.2 (C-4), 120.8 (C-5), 120.1 (C-6), 119.6 (C-3), 114.0 (C-1), 111.4 (C-8), 106.8 (C-4´), 37.5 (CH_3_). **MS (EI):** *m/z* (rel. int. in %) = 247 [M^+^**^•^**] (100), 231 (10), 219 (40), 191 (10), 124 (15), 110 (10). **HR-MS (EI):** *m/z* found 247.1107, calcd. for C_16_H_13_N_3_: 247.1109.

**1-(Pyridin-2-yl)-9*H*-carbazole (13)**

| A solution of 150 mg (0.61 mmol) 1-bromo-9*H*-carbazole under N_2_ atmosphere was cooled to –78 °C and treated with 1.3 mL (2.4 mmol) of a 1.9 M solution of *tert*-butyllithium (in pentane). The mixture was allowed to warm up to –20 °C under stirring over 2 h, then 59 µL (0.73 mmol) pyridine was added with a syringe, and the mixture was allowed to come to room temperature under stirring for 2 h. After addition of water (30 mL) the mixture was extracted with ethyl acetate (3 x 30 mL). The combined organic layers were dried over Na_2_SO_4_ and evaporated. The residue was purified by silica gel chromatography using dichlormethane/hexanes/triethylamine 5:5:1). **Yield:** 35 mg (23 %), pale brown solid, **Mpt:** 145-146 °C. **^1^H-NMR (500 MHz, CDCl_3_):** δ (ppm) = 11.44 (br s, 1H, NH), 8.78 (ddd, *J* = 4.9 Hz, 1.8 Hz, 0.9 Hz, 1H, 6´-H), 8.15 (d, *J* = 7.7 Hz, 1H, 4-H), 8.12 (dd, *J* = 7.8 Hz, 1.0 Hz, 1H, 5-H), 8.02 (d, *J* = 8.2 Hz, 1H, 3´-H), 7.96 (d, *J* = 7.7 Hz, 1H, 2-H), 7.78 (ddd, *J* = 8.1 Hz, 7.5 Hz, 1.9 Hz, 1H, 4´-H), 7.57 (d, *J* = 8.1 Hz, 1H, 8-H), 7.45 (td, *J* = 8.1 Hz, 1.1 Hz, 1H, 7-H), 7.30 (t, *J* = 7.7 Hz, 1H, 3-H), 7.25 (t, *J* = 7.8 Hz, 1H, 6-H), 7.22 (ddd, *J* = 7.4 Hz, 4.8 Hz, 1.0 Hz, 1H, 5´-H). **^13^C-NMR (100 MHz, CDCl_3_):** δ (ppm) = 157.7 (C-2´), 148.6 (C-6´), 139.7 (C-8a), 138.5 (C-9a), 136.7 (C-4´), 125.9 (C-7), 124.7 (C-4a), 123.0 (C-2), 122.7 (C-4b), 121.6 (C-4), 121.3 (C-5´), 120.3 (C-5), 120.0 (C-3´, C-1), 119.1 (C-6), 118.7 (C-3), 111.1 (C-8). **MS (EI):** *m/z* (rel. int. in %) = 244 [M^+^**^•^**] (100), 122 (30), 108 (10). **HR-MS (EI):** *m/z* found 244.0999, calcd. for C_17_H_12_N_2_: 244.1001. | |
| --- | --- |
|  |  |

**1-(Pyrimidin-4-yl)-9*H*-carbazole (14)**

| Prepared in the same manner as described for compound **13**; from 300 mg (1.22 mmol) 1-bromo-9*H*-carbazole using 4.88 mmol of *tert*-butyllithium and 1.46 mmol pyrimidine. Purification by silica gel chromatography using dichloromethane/ethyl acetate 1:1). **Yield:** 120 mg (40 %), yellow solid. **Mpt:** 116-118 °C. **^1^H-NMR (400 MHz, CD_2_Cl_2_):** δ (ppm) = 11.37 (br s, 1H, NH), 9.33 (s, 1H, 2´-H), 8.75 (d, *J* = 5.6 Hz, 1H, 6´-H), 8.23 (d, *J* = 7.6 Hz, 1H, 4-H), 8.12 (d, *J* = 7.8 Hz, 1H, 5-H), 8.02 (d, *J* = 7.8 Hz, 1H, 2-H), 7.92 (d, *J* = 5.6 Hz, 1H, 5´-H), 7.60 (d, *J* = 8.1 Hz, 1H, 8-H), 7.48 (t, *J* = 7.7 Hz, 1H, 7-H), 7.31 (t, *J* = 7.7 Hz, 1H, 3-H), 7.27 (t, *J* = 7.9 Hz, 1H, 6-H). **^13^C-NMR (100 MHz, CD_2_Cl_2_):** δ (ppm) = 164.4 (C-4´), 158.7 (C-2´), 157.4 (C-6´), 140.1 (C-8a), 139.2 (C-9a), 126.8 (C-7), 125.4 (C-4a), 124.3 (C-2), 124.1 (C-4), 122.6 (C-4b), 120.7 (C-5), 120.6 (C-6), 119.2 (C-3), 117.6 (C-1), 116.5 (C-5´), 111.6 (C-8). **MS (EI):** *m/z* (rel. int. in %) = 245 [M^+^**^•^**] (100), 191 (10), 168 (10), 157 (10), 123 (15), 109 (10), 85 (15), 64 (10). **HR-MS (EI):** *m/z* found 245.0959, calcd. for C_16_H_11_N_3_: 245.0953. |
| --- |

***N*-[2-(9*H*-Carbazol-1-yl)pyridin-3-yl]acetamide (16)**

A solution of 100 mg (0.266 mmol) 1-bromo-9-{[2-(trimethylsilyl)ethoxy]methyl}-9*H*-carbazole (N-SEM-carbazole) and 10 mg (0.0087 mmol) Pd(PPh_3_)_4_ in 2 mL anhydrous dioxane was purged with N_2_, then heated to 80 °C, and 380 µL (2.72 mmol) triethylamine and 118 µL (0.758 mmol) 4,4,5,5-tetramethyl-1,3,2-dioxaborolane were added slowly. After heating at 80 °C for 3 h another 116 µL (0.799 mmol) 4,4,5,5-tetramethyl-1,3,2-dioxaborolane was added and the mixture heated at 80 °C for 12 h. After cooling to ambient temperature 3 mL methanol, 35 mg (0.27 mmol) 3-amino-2-chloropyridine, and 220 mg (0.675 mmol) cesium carbonate were added, and the mixture was stirred for 12 h at 100 °C. After cooling 40 mL water was added, followed by extraction with diethyl ether (3 x 50 mL). The combined organic layers were dried over Na_2_SO_4_ and evaporated. The residue was purified by silica gel chromatography using ethyl acetate/hexane (1:9 – 9:1). The product was dissolved in 3 mL dichloromethane, cooled to 0 °C and treated with 45 µL (0.56 mmol) pyridine and 30 µL (0.42 mmol) acetyl chloride. After stirring for 2 h at ambient temperature 10 mL water was added, followed by extraction with dichloromethane (3 x 20 mL). The combined organic layers were dried over Na_2_SO_4_ and evaporated. The residue was purified by silica gel chromatography using ethyl acetate/hexane (1:1). The product was refluxed with 2 mL tetrabutylammonium fluoride in THF (1M) for 16 h, then 20 mL water was added, followed by extraction with ethyl acetate (3 x 20 mL). The combined organic layers were dried over Na_2_SO_4_ and evaporated. The residue was purified by silica gel chromatography using ethyl acetate/hexane (9:1). The product was recrystallised from pentane to give 23 mg (29 %) **16** as white crystals.

**Mpt:** 214 °C. **^1^H-NMR (400 MHz, acetone-d_6_):** δ (ppm) = 10.43 (bs, 1H, 9´´-H), 8.68 (bs, 1H, 1-NH), 8.53 (d, *J* = 8.1 Hz, 1H, 4´-H), 8.48 (dd, *J* = 4.6, 1.6 Hz, 1H, 6´-H), 8.23 – 8.13 (m, 2H, 4´´-H, 5´´-H), 7.65 (dd, *J* = 7.4, 1.1 Hz, 1H, 2´´-H), 7.55 (dt, *J* = 8.1, 0.9 Hz, 1H, 8´´-H), 7.45 – 7.35 (m, 2H, 5´-H, 7´´-H), 7.29 (t, *J* = 7.6 Hz, 1H, 3´´-H), 7.21 (ddd, *J* = 8.0, 7.1, 1.0 Hz, 1H, 6´´-H), 2.02 (s, 3H, 2-H). **^13^C-NMR (101 MHz, acetone-d_6_):** δ (ppm) = 169.58 (C-1), 149.45 (C-2´), 145.88 (C-6´), 141.27 (C-8´´a), 139.37 (C-9´´a), 133.91 (C-3´), 132.28 (C-5´), 126.71 (C-7´´), 126.58 (C-2´´), 125.27 (C-4´´a), 123.84 (C-4´´b), 123.20 (C-4´), 121.66 (C-1´´), 121.47 (C-4´´), 120.87 (C-5´´), 119.92 (C-3´´), 119.57 (C-6´´), 112.12 (C-8´´), 24.04 (C-2). **HR-MS (EI):** *m/z* found 301.1213, calcd. for C_19_H_15_N_3_O: 301.1215.

**3-(9H-carbazol-1-yl)pyridin-2-amine (17b)**

A degassed stirred solution of 1-bromo-9*H*-carbazole (0.5 g, 2.03 mmol, 1 eq), 3-(4,4,5,5-tetramethyl-1,3,2-dioxaborolan-2-yl)pyridin-2-amine (0.492 g, 2.24 mmol, 1.1 eq), Na_2_CO_3_ (0.646 g, 6.09 mmol, 3 eq) and Pd(PPh_3_)_4_ (117 mg, 0.102 mmol, 0.05 eq) in a mixture of 1,4-Dioxane:EtOH:H_2_O (5 mL, 2:1:1, 0.5 M) under an inert N_2_ atmosphere was heated with microwave irradiation at 90°C for 2 h. Following completion the crude mixture was concentrated onto silica gel and purified by Isolera Biotage LPLC (CH/EA 8:2) to give **17b** (128 mg, 24 %) as a beige solid.

**v_max_ (cm^-1^)** 3450, 3353, 1590, 1447, 1417, 1317, 1237, 849; **^1^H-NMR (400MHz, CDCl_3_): δ_H_** 8.79 (br. s., 1H), 8.04 - 8.30 (m, 3H), 7.60 (dd, J = 1.83, 7.34 Hz, 1H), 7.39 - 7.50 (m, 3H), 7.33 - 7.38 (m, 1H), 7.24 - 7.30 (m, 1H), 6.84 (dd, J= 7.3, 5.1 Hz, 1H), 4.53 (br. s., 2H); **^13^C-NMR (100 MHz CDCl_3_): δ_C_** 155.8, 147.9, 139.8, 139.0, 137.3, 126.7, 126.2, 123.9, 123.3, 120.4, 120.2, 119.9, 119.7, 118.8, 114.8, 110.9; **LR-ESI-MS**: C_17_H_14_N_3_ [M+H]^+^ *m/z* found 260.10, cald 260.12.

***N*-[2-(4-Oxo-4*H*-pyrido[3,2,1-*jk*]carbazol-5-yl)phenyl]acetamide (20)**

| Prepared following the General Procedure for the “First generation 1-(heteroaryl)carbazoles (**3-14**) prepared by Suzuki-Miyaura cross-coupling of 1-bromo-9*H*-carbazole with (hetero)arylboronic acids“ utilizing 100 mg (0.290 mmol) 5-iodo-4*H*-pyrido[3,2,1-*jk*]carbazol-4-one, 59 mg (0.33 mmol) 2-acetamidophenylboronic acid, and 38 mg (0.033 mmol) Pd(Ph_3_P)_4_. Purification of the crude product by silica gel chromatography using ethyl acetate/hexane (5:1) gave the compound as a brownish-red solid. Yield: 8 mg (8 %). **Mpt:** 230-232 °C. **^1^H-NMR (400 MHz, CD_2_Cl_2_):** δ (ppm) = 8.85 (s, 1H), 8.51 (s, 1H), 8.49 (d, *J* = 8.2 Hz, 1H), 8.36 (d, *J* = 7.4 Hz, 1H), 8.16 (d, *J* = 7.7 Hz, 1H), 7.87 (d, *J* = 8.3 Hz, 1H), 7.82 – 7.75 (m, 2H), 7.62 (t, *J* = 7.7 Hz, 1H), 7.56 – 7.44 (m, 3H), 7.36 (d, *J* = 7.3 Hz, 1H), 2.05 (s, 3H). **^13^C-NMR:** data not available due to solubility issues. **MS (EI):** *m/z* (rel. int. in %) = 352 [M^+^**^•^**] (5), 293 (10), 277 (100), 219 (15), 199 (20), 183 (20), 152 (10), 77 (20), 51 (10). **HR-MS (EI):** *m/z* found 352.1175, calcd. for C_23_H_16_N_2_O_2_: 352.1212. |
| --- |

***N*-[3-(9*H*-Carbazol-1-yl)phenyl]acetamide (21)**

A solution of 50 mg (0.19 mmol) 3-(9*H*-carbazol-1-yl)aniline, prepared in 88 % yield from 1-bromo-9*H*-carbazole and 3-aminophenylboronic acid following the General Procedure for the “First generation 1-(heteroaryl)carbazoles (**3-14**) prepared by Suzuki-Miyaura cross-coupling of with (hetero)arylboronic acids“ in 1 mL dichloromethane was treated at 0 °C with 18 µL (0.22 mmol) pyridine and 15 µL (0.21 mmol) acetyl chloride and stirred at ambient temperature for 2 h. The solution was washed with water (3 x 10 mL) and satd. NaHCO_3_ solution, dried over Na_2_SO_4_ and evaporated. The residue was purified by silica gel chromatography using ethyl acetate/dichloromethane (1:9) to give 54 mg (93 %) **21** as a pale yellow solid.

**Mpt:** 220 °C. **^1^H-NMR (500 MHz, acetone-d_6_):** δ (ppm) = 10.38 (bs, 1H, 1-NH), 9.32 (bs, 1H, 9´´-H), 8.17 – 8.12 (m, 2H, 4´´-H, 5´´-H), 7.94 (t, *J* = 1.9 Hz, 1H, 2´-H), 7.79 (ddd, *J* = 8.1, 2.2, 1.2 Hz, 1H, 4´-H), 7.55 (dt, *J* = 8.1, 0.9 Hz, 1H, 8´´-H), 7.45 (t, *J* = 7.8 Hz, 1H, 5´-H), 7.44 – 7.35 (m, 3H, 6´-H, 2´´-H, 7´´-H), 7.29 (t, *J* = 7.6 Hz, 1H, 3´´-H), 7.20 (ddd, *J* = 8.0, 7.1, 1.0 Hz, 1H, 6´´-H), 2.12 (s, 3H, 2-H). **^13^C-NMR (126 MHz, acetone-d_6_):** δ (ppm) = 169.02 (C-1), 141.21 (C-8´´a), 140.97 (C-1´), 140.42 (C-3´), 138.30 (C-9´´a), 130.19 (C-5´), 126.53 (C-7´´), 126.19 (C-2´´), 125.89 (C-4´´b), 124.61 (C-1´´), 124.05 (C-6´), 123.99 (C-4´´a), 120.90 (C-5´´), 120.21 (C-3´´), 120.19 (C-4´´), 119.97 (C-6´´), 119.84 (C-2´), 118.91 (C-4´), 112.06 (C-8´´), 24.28 (C-2). **MS (ESI):** m/z (rel. int. in %) = 299 (100) (M - H)^-^. **HR-MS (ESI):** *m/z* found 299.1188, calcd. for C_20_H_15_N_2_O^-^ [M - H]^-^: 299.1184.

***N*-[2-(9*H*-Carbazol-9-yl)phenyl]acetamide (22)**

200 mg (0.696 mmol) 9-(2-nitrophenyl)-9*H*-carbazole, 194 mg (3.47 mmol) iron powder, and 19 mg (0.35 mmol) ammonium chloride in 6 mL ethanol-water (2:1) was heated at 100 °C for 90 min, then coolded to ambient temperature, filtered, and extracted with ethyl acetate. The organic layer were dried over Na_2_SO_4_ and evaporated. The residue was dissolved in 3 mL dichloromethane, treated at 0 °C with 61 µL (0.76 mmol) pyridine and 52 µL (0.73 mmol) acetyl chloride. After 15 h another 61 µL (0.76 mmol) pyridine and 52 µL (0.73 mmol) acetyl chloride was added. After stirring for 2 h 50 mL dichloromethane was added, and the solution was washed with water (3 x 40 mL) and satd. NaHCO_3_ solution, dried over Na_2_SO_4_ and evaporated. The residue was purified by silica gel chromatography using dichloromethane/hexane (9:1) to give 132 mg (63 %) **22** as a white solid.

**Mpt:** 68 °C. **^1^H-NMR (400 MHz, CD_2_Cl_2_):** δ (ppm) = 8.49 (d, *J* = 8.2 Hz, 1H, 6´-H), 8.18 (ddd, *J* = 7.7, 1.2, 0.7 Hz, 2H, 4´´-H, 5´´-H), 7.57 – 7.51 (m, 1H, 5´-H), 7.42 (ddd, *J* = 8.3, 7.2, 1.2 Hz, 2H, 2´´-H, 7´´-H), 7.39 – 7.26 (m, 4H, 3´´-H, 6´´-H, 4´-H, 3´-H), 7.13 (d, *J* = 8.2 Hz, 2H, 1´´-H, 8´´-H), 6.92 (br s, 1H, 1-NH), 1.74 (s, 3H, 2-H). **^13^C-NMR (126 MHz, CD_2_Cl_2_):** δ (ppm) = 168.8 (C-1), 141.4 (C-8´´a, C-9´´a), 136.2 (C-1´, C-2´), 129.8 (C-5´), 129.3 (C-3´), 126.9 (C-2´´, C-7´´), 125.3 (C-4´), 124.3 (C-4´´a, C-4´´b), 122.8 (C-6´), 121.3 (C-4´´, C-5´´), 121.0 (C-3´´, C-6´´), 110.5 (C-1´´, C-8´´), 24.9 (C-2). **MS (ESI):** m/z (rel. int. in %) = 299 (100) (M - H)^-^. **HR-MS (ESI):** *m/z* found 299.1186, calcd. for C_20_H_15_N_2_O^-^ [M - H]^-^: 299.1184.

***N*-{3-[(9*H*-Carbazol-9-yl)methyl]phenyl}acetamide (23)**

Prepared in the same manner as described for **22** by reduction of 300 mg (0.992 mmol) 9-(3-nitrobenzyl)-9*H*-carbazole with 277 mg (4.96 mmol) iron powder and 27 mg (0.49 mmol) ammonium chloride, followed by *N*-acetylation of the crude primary amine with acetyl chloride and pyridine. Purification by silica gel chromatography using dichloromethane/ethyl acetate (99:1) gave 112 mg (38 %) **23** as a beige solid.

**Mpt:** 178 °C. **^1^H-NMR (500 MHz, CD_2_Cl_2_):** δ (ppm) = 8.15 – 8.10 (m, 2H, 4´´´-H, 5´´´-H), 7.47 (d, *J* =7.8 Hz, 1H, 5´-H), 7.43 (ddd, *J* = 8.2, 7.0, 1.2 Hz, 2H, 2´´´-H, 7´´´-H), 7.38 (d, *J* = 8.2 Hz, 2H, 1´´´-H, 8´´´-H), 7.25 (ddd, *J* = 8.0, 7.0, 1.1 Hz, 2H, 3´´´-H, 6´´´-H), 7.21 (d, *J*=7.9 Hz, 1H, 6´-H), 7.18 (br s, 1H, 1-NH), 7.15 (s, 1H, 2´-H), 6.90 (d, *J* = 7.7 Hz, 1H, 4´-H), 5.51 (s, 2H, 1´´-H), 2.00 (s, 3H, 2-H). **^13^C-NMR (126 MHz, CD_2_Cl_2_):** δ (ppm) = 168.7 (C-1), 141.1 (C-3´), 139.3 (C-1´), 138.9 (C-8´´´a, C-9´´´a), 129.9 (C-4´), 126.4 (C-2´´´, C-7´´´), 123.4 (C-4´´´a, C-4´´´b), 122.5 (C-5´), 120.8 (C-4´´´, C-5´´´), 119.8 (C-3´´´, C-6´´´), 119.2 (C-6´), 117.7 (C-2´), 109.4 (C-1´´´, C-8´´´), 46.9 (C-1´´), 24.8 (C-2). **MS (EI):** m/z (rel. int. in %) = 314 (100) [M^+.^], 148 (65), 106 (40). **HR-MS (EI):** *m/z* found 314.1408, calcd. for C_21_H_18_N_2_O: 314.1419.

***N*-[2-(9-Methyl-9*H*-carbazol-1-yl)phenyl]acetamide (24)**

2-(9-Methyl-9*H*-carbazol-1-yl)aniline was prepared in 96% yield from 1-bromo-9-methyl-9*H*-carbazole and 2-aminophenylboronic acid following the General Procedure for the “First generation 1-(heteroaryl)carbazoles (**3-14**) prepared by Suzuki-Miyaura cross-coupling of with (hetero)arylboronic acids“. *N*-Acetylation of the crude primary amine with acetyl chloride and pyridine was performed in the same manner as described for the preparation of **23**. Purification by silica gel chromatography using dichloromethane/hexane (4:1) gave 102 mg (86 %) **23** as a pale pink solid.

**Mpt:** 75 °C. **^1^H-NMR (400 MHz, CD_2_Cl_2_):** δ (ppm) = 8.35 (d, *J* = 8.0 Hz, 1H, 6´-H), 8.20 (dd, *J* = 7.5, 1.5 Hz, 1H, 4´´-H), 8.15 (ddd, *J* = 7.8, 1.3, 0.7 Hz, 1H, 5´´-H), 7.52 – 7.43 (m, 2H, 5´-H, 7´´-H), 7.41 – 7.35 (m, 2H, 3´-H, 8´´-H), 7.32 (t, *J* = 7.4 Hz, 1H, 3´´-H), 7.29 – 7.25 (m, 2H, 2´´-H, 6´´-H), 7.22 (td, *J* = 7.5, 1.3 Hz, 1H, 4´-H), 6.90 (bs, 1H, 1-NH), 3.30 (s, 3H, 1´´´-H), 1.74 (s, 3H, 2-H). **^13^C-NMR (126 MHz, CD_2_Cl_2_):** δ (ppm) = 161.95 (C-1), 142.64 (C-8´´a), 138.93 (C-9´´a), 137.27 (C-1´), 131.59 (C-3´), 130.19 (C-2´), 129.25 (C-2´´), 129.11 (C-5´), 126.75 (C-7´´), 124.76 (C-4´´a), 124.04 (C-4´), 123.06 (C-4´´b), 121.12 (C-6´), 120.96 (C-4´´), 120.65 (C-1´´), 120.56 (C-5´´), 119.89 (C-6´´-), 119.70 (C-3´´), 109.55 (C-8´´), 31.79 (C-1´´´), 24.85 (C-2). **MS (ESI):** m/z (rel. int. in %) = 313 (100) (M - H)^-^. **HR-MS (ESI):** *m/z* found 313.1351, calcd. for C_21_H_17_N_2_O^-^ [M - H]^-^: 313.1341.

***N*-[2-(9-Benzyl-9*H*-carbazol-1-yl)phenyl]acetamide (25)**

2-(9-Benzyl-9*H*-carbazol-1-yl)aniline was prepared in 87% yield from 1-bromo-9-benzyl-9*H*-carbazole and 2-aminophenylboronic acid following the General Procedure for the “First generation 1-(heteroaryl)carbazoles (**3-14**) prepared by Suzuki-Miyaura cross-coupling of with (hetero)arylboronic acids“. N-Acetylation of the crude primary amine with acetyl chloride and pyridine was performed in the same manner as described for the preparation of **23**. Purification by silica gel chromatography using dichloromethane/hexane (4:1) gave 90 mg (83 %) **25** as a white solid.

**Mpt:** 75 °C. **^1^H-NMR (400 MHz, CD_2_Cl_2_):** δ (ppm) = 8.25 (dd, *J* = 7.8, 1.3 Hz, 1H, 4´´-H), 8.22 (ddd, *J* = 7.8, 1.3, 0.8 Hz, 2H, 6´-H, 5´´-H), 7.46 (ddd, *J* = 8.3, 7.1, 1.3 Hz, 1H, 7´´-H), 7.40 – 7.28 (m, 4H, 5´-H, 3´´-H, 6´´-H, 8´´-H), 7.18 (dd, *J* = 7.3, 1.3 Hz, 1H, 2´´-H), 7.13 – 7.07 (m, 2H, 3´-H ,4´´´´-H), 7.07 – 6.98 (m, 3H, 4´-H, 3´´´´-H, 5´´´´-H), 6.48 (s, 1H, 1-NH), 6.42 – 6.36 (m, 2H, 2´´´´-H, 6´´´´-H), 5.19 – 5.03 (m, 2H, 1´´´-H), 1.62 (s, 3H, 2-H). **^13^C-NMR (126 MHz, CD_2_Cl_2_):** δ (ppm) = 168.22 (C-1), 142.57 (C-8´´a), 138.26 (C-1´´´´), 138.22 (C-9´´a), 137.21 (C-1´), 131.49 (C-3´), 129.47 (C-2´), 129.46 (C-2´´), 129.23 (C-5´), 128.76 (C-3´´´´, C-5´´´´), 127.52 (C-4´´´´), 126.98 (C-7´´), 125.99 (2´´´´, C-6´´´´), 125.48 (C-4´´a), 123.69 (C-4´), 123.34 (C-4´´b), 121.09 (C-6´), 121.02 (C-4´´), 121.00 (C-1´´), 120.74 (C-5´´), 120.35 (C-3´´), 120.13 (C-6´´), 110.07 (C-8´´), 47.89 (C-1´´´), 24.81 (C-2). **MS (ESI):** m/z (rel. int. in %) = 389 (100) (M - H)^-^. **HR-MS (ESI):** *m/z* found 389.1665, calcd. for C_27_H_21_N_2_O^-^ [M - H]^-^: 389.1654.

***N*-[2-(Dibenzofuran-4-yl)phenyl]acetamide (26)**

A degassed solution of 200 mg (0.809 mmol) 4-bromodibenzofuran,145 mg (0.810 mmol) 2-acetamidophenylboronic acid and 94 mg (0.081mmol) Pd(Ph_3_P)_4_ in 10 mL 1,2-dimethoxyethane and 5 mL ethanol under nitrogen was srirred for 10 min, then treated with 4 mL degassed 2M sodium carbonate solution. The mixture was heated at 90 °C for 15 h, then poured into 100 mL water and extracted with diethyl ether (4 x 60 mL). The combined organic layers were dried over Na_2_SO_4_ and evaporated. The residue was purified by silica gel chromatography using dichloromethane/hexane (9:1) to give 126 mg (52 %) **26** as a white solid.

**Mpt:** 78 °C. **^1^H-NMR (400 MHz, CD_2_Cl_2_):** δ (ppm) = 8.18 (d, *J* = 8.1 Hz, 1H, 6´-H), 8.10 – 8.00 (m, 2H, 1´´-H, 9´´-H), 7.57 (d, *J* = 8.2 Hz, 1H, 6´´-H), 7.54 – 7.44 (m, 5H, 3´-H, 5´-H, 2´´-H, 3´´-H, 7´´-H), 7.41 (td, *J* = 7.5, 1.1 Hz, 1H, 8´´-H), 7.29 (t, *J* = 7.5 Hz, 1H, 4´-H), 7.22 (bs, 1H, 1-NH), 1.82 (s, 3H, 2-H). **^13^C-NMR (126 MHz, CD_2_Cl_2_):** δ (ppm) = 168.76 (C-1), 156.64 (C-5´´a), 153.81 (C-4´´a), 136.27 (C-1´), 131.53 (C-3´), 129.40 (C-2´´), 129.23 (C-3´´), 128.39 (C-2´), 128.26 (C-7´´), 125.40 (C-4´´), 125.16 (C-4´), 124.60 (C-9´´a), 124.15 (C-5´), 123.76 (C-8´´), 123.51 (C-6´), 122.92 (C-9´´b), 121.46 (C-1´´), 121.28 (C-9´´), 112.27 (C-6´´), 24.70 (C-2). **MS (EI):** m/z (rel. int. in %) = 301 (50) [M^+.^], 283 (20), 259 (100), 230 (20), 204 (15). **HR-MS (EI):** *m/z* found 301.1099, calcd. for C_20_H_15_NO_2_: 301.1103.

***N*-[2-(Dibenzothiophen-4-yl)phenyl]acetamide (27)**

Prepared from 462 mg (1.49 mmol) 4-iododibenzothiophene and 322 mg (1.80 mmol) 2-Acetamidophenylboronic acid in the same manner as described for **26**. Purification by silica gel chromatography using dichloromethane/hexane (9:1 to 1:9), followed by crystallisation from pentane gave 238 mg (50 %) **27** as a pale brown solid.

**Mpt:** 79 °C. **^1^H-NMR (400 MHz, CD_2_Cl_2_):** δ (ppm) = 8.31 (d, *J* = 8.3 Hz, 1H, 9´´-H), 8.30 – 8.21 (m, 2H, 1´´-H, 6´-H), 7.86 – 7.82 (m, 1H, 7´´-H), 7.63 (dd, *J* = 7.9, 7.3 Hz, 1H, 2´´-H), 7.53 – 7.49 (m, 2H, 5´-H, 6´´-H), 7.48 – 7.45 (m, 1H, 8´´-H), 7.43 (dd, *J* = 7.4, 1.2 Hz, 2H, 3´-H, 3´´-H), 7.26 (t, *J* = 7.5 Hz, 1H, 4´-H), 6.99 (bs, 1H, 1-NH), 1.83 (s, 3H, 2-H). **^13^C-NMR (126 MHz, CD_2_Cl_2_):** δ (ppm) = 168.69 (C-1), 140.61 (C-4´´a), 140.04 (C-1´), 136.90 (C-4´´), 136.23 (C-5´´a), 135.90 (C-9´´a), 133.18 (C-9´´b), 130.85 (C-2´), 130.46 (C-5´), 129.69 (C-3´), 128.44 (C-3´´), 127.70 (C-6´´), 125.83 (C-2´´), 125.22 (C-9´´), 124.82 (C-4´), 123.38 (C-7´´), 122.44 (C-6´, C-8´´), 121.93 (C-1´´), 24.91 (C-2). **MS (EI):** m/z (rel. int. in %) = 317 (40) [M^+.^], 275 (100), 184 (25). **HR-MS (EI):** *m/z* found 317.0873, calcd. for C_20_H_15_NOS: 317.0874.

***N*-[2-(9-Oxo-9*H*-fluoren-1-yl)phenyl]acetamide (28)**

Prepared from 102 mg (0.334 mmol) 1-iodo-9*H*-fluoren-9-one and 60 mg (0.33 mmol) 2-acetamidophenylboronic acid using 40 mg (0.035 mmol) Pd(Ph_3_P)_4_. Tetrakis(triphenylphosphin)palladium(0) following the General Procedure for the “First generation 1-(heteroaryl)carbazoles (**3-14**) prepared by Suzuki-Miyaura cross-coupling of with (hetero)arylboronic acids“. Purification by silica gel chromatography using ethyl acetate/hexane (3:7) gave 83 mg (79 %) **28** as a yellow solid.

**Mpt:** 176 °C. **^1^H-NMR (500 MHz, CD_2_Cl_2_):** δ (ppm) = 7.99 (d, *J* = 8.2 Hz, 1H, 6‘-H), 7.65 – 7.60 (m, 2H, 4´´-H, 6´´-H), 7.56 (ddd, *J* = 17.2, 8.4, 6.9 Hz, 3H, 3´´-H, 5´´-H, 8´´-H), 7.43 (dt, *J* = 8.6, 4.7 Hz, 1H, 5‘-H), 7.38 – 7.30 (m, 1H, 7´´-H), 7.24 (d, *J* = 4.5 Hz, 2H, 4‘-H, 3‘-H), 7.15 (d, *J* = 7.5 Hz, 1H, 2´´-H), 7.00 (bs, 1H, 1-NH), 1.86 (s, 3H, 2-H). **^13^C-NMR (126 MHz, CD_2_Cl_2_):** δ (ppm) = 193.7 (C-9´´), 168.7 (C-1), 145.9 (C-4´´a), 144.2 (C-4´´b), 137.8 (C-8´´a), 136.1 (C-1’), 135.4 (C-3´´), 135.4 (C-5´´), 134.5 (C-2’), 132.6 (C-2´´), 131.2 (C-9´´a), 131.1 (C-1´´), 130.6 (C-3’), 129.9 (C-7´´), 129.3 (C-5’), 125.1 (C-4’), 124.6 (C-8´´), 123.8 (C-6’), 120.9 (C-6´´), 120.8 (C-4´´), 24.5 (C-2). **MS (EI):** m/z (rel. int. in %) = 313 (24) [M^+.^], 254 (100). **HR-MS (EI):** *m/z* found 313.1099, calcd. for C_21_H_15_NO_2_: 313.1103.

***N*-[2-(9-Hydroxy-9*H*-fluoren-1-yl)phenyl]acetamide (29)**

A solution of 54 mg (0.17 mmol) *N*-(2-(9-oxo-9*H*-fluoren-1-yl)phenyl)acetamide (**28**) in 1 mL THF was treated with 36 mg (0.94 mmol) sodium borohydride and stirred at 75 °C for 3 h. After addition of water the mixture was extracted with dichloromethane (3 x 15 mL), the combined organic layers were dried over Na_2_SO_4_ and evaporated. The residue was purified by silica gel chromatography using hexane/ethyl acetate (7:3) to give 28 mg (52 %) **29** as a white solid.

**Mpt:** 115 °C. **^1^H-NMR (400 MHz, CD_2_Cl_2_):** δ (ppm) = 8.27 (bs, 1H, 1-NH), 7.81 (d, *J* = 8.4 Hz, 1H, 4‘-H), 7.72 (d, *J* = 7.6 Hz, 2H, 2´´-H, 6‘-H), 7.53 (d, *J* = 7.4 Hz, 1H, 8´´-H), 7.49 – 7.35 (m, 4H, 4´´-H, 6´´-H, 5‘-H, 3‘-H), 7.32 (td, *J* = 7.4 Hz, 1.1 Hz, 2H, 5´´-H, 7´´-H), 7.09 (d, *J* = 7.6 Hz, 1H, 3´´-H), 5.67 (s, 1H, 9´´-H), 3.19 (bs, 1H, 9´´-OH), 1.82 (s, 3H, 2-H). **^13^C-NMR (126 MHz, CD_2_Cl_2_):** δ (ppm) = 169.0 (C-1), 146.6 (C-8´´a), 144.5 (C-1’), 141.7 (C-4´´a), 140.2 (C-4´´b), 137.0 (C-1´´), 135.8 (C-9´´a), 134.5 (C-2’), 129.9 (C-6´´), 129.8 (C-4´´), 129.5 (C-3´´), 129.2 (C-5’), 129.1 (C-2´´), 128.6 (C-7´´), 126.1 (C-3’), 125.6 (C-8´´), 124.7 (C-4’), 120.6 (C-5´´), 120.2 (C-6’), 74.3 (C-9´´), 24.2 (C-2). **MS (EI):** m/z (rel. int. in %) = 315 (20) [M^+.^], 272 (30), 254 (100). **HR-MS (EI):** *m/z* found 315.1246, calcd. for C_21_H_17_NO_2_: 315.1259.

***N*-[2'-(Phenylamino)-(1,1'-biphenyl)-2-yl]acetamide (31)**

A dispersion of 200 mg (0.884 mmol) *N*-(2'-amino-[1,1'-biphenyl]-2-yl)acetamide (**30**), 100 µL (0.897 mmol) iodbenene, 2 mg (0.002 mmol) Pd_2_(dba)_3_, 4 mg (0.006 mmol) BINAP and 119 mg (1.06 mmol) potassium *tert-*butanolate in 2 mL toluene was heated under N_2_ at 110°C for 12 h. after cooling 5 mL satd. ammonium chloride solution was added and the mixture extracted with diethyl ether (4 x 30 mL). The combined organic layers were dried over Na_2_SO_4_ and evaporated. The residue was purified by silica gel chromatography using dichloromethane/ethyl acetate (9:1), but only impure product was obtained. Thus the product was submittd to amide hydrolysis (500 mg NaOH in 5 mL ethanol, 90 °C, 4 h). After neutralisation the free amino compound was collected by extraction with ethyl acetate (3 x 30 mL), purified by silica gel chromatography using dichloromethane/hexane (1:1), and submitted to N-acetylation in 5 mL dichloromethane with 100 µL (1.23 mmol) pyridine and 80 µL (0.85 mmol) acetanhydride at 0 °C for 2 h. The solution was washed with satd. sodium bicarbonate solution, dried over Na_2_SO_4_ and evaporated. The residue was purified by silica gel chromatography using dichloromethane/ethyl acetate (9:1) to give 82 mg (31 %) **31** as a white solid.

**Mpt:** 52 °C. **^1^H-NMR (500 MHz, methanol-d_4_):** δ (ppm) = 7.58 (dt, *J* = 8.0, 0.9 Hz, 1H, 3´-H), 7.41 – 7.33 (m, 2H, 4´-H, 3´´-H), 7.31 – 7.25 (m, 3H, 5´-H, 6´-H, 4´´-H), 7.20 – 7.12 (m, 3H, 6´´-H, 5´´´´-H, 3´´´´-H), 7.01 (td, *J* = 7.5, 1.2 Hz, 1H, 5´´-H), 6.99 – 6.94 (m, 2H, 2´´´´-H, 6´´´´-H), 6.82 (tt, *J* = 7.5, 1.1 Hz, 1H, 4´´´´-H), 1.81 (s, 3H, 2-H). **^13^C-NMR (126 MHz, methanol-d_4_):** δ (ppm) = 172.44 (C-1), 145.12 (C-1´´´´), 142.28 (C-2´´), 136.46 (C-1´), 135.57 (C-2´), 132.73 (C-6´´), 132.63 (C-4´´), 130.67 (C-1´´), 130.12 (C-3´´´´, C-5´´´´), 129.61 (C-6´), 129.24 (C-4´), 127.52 (C-5´), 127.30 (C-3´), 122.38 (C-5´´), 121.46 (C-4´´´´), 119.67 (C-3´´), 118.37 (C-2´´´´, C-6´´´´), 22.99 (C-2). **MS (EI):** m/z (rel. int. in %) = 302 (65) [M^+.^], 243 (100), 167 (55). **HR-MS (EI):** *m/z* found 302.1438, calcd. for C_20_H_18_N_2_O: 302.1419.

**Methyl 2-[(2'-acetamido-[1,1'-biphenyl]-2-yl)amino]benzoate (32)**

A dispersion of 400 mg (1.77 mmol) *N*-(2'-amino-[1,1'-biphenyl]-2-yl)acetamide (**30**), 265 µL (1.80 mmol) methyl 2-iodbenzoate, 3.5 mg (0.0038 mmol) Pd_2_(dba)_3_, 6.6 mg (0.011 mmol) BINAP, and 242 mg (2.16 mmol) potassium *tert-*butanolate in 4 mL toluene under N_2_ was heated at 110 °C for 12 h. After cooling 20 mL satd. ammonium chloride solution was added and the mixture extracted with diethyl ether (3 x 50 mL) and ethyl acetate (2 x 40 mL). The combined organic layers were dried over Na_2_SO_4_ and evaporated. The residue was purified by silica gel chromatography using dichloromethane/ethyl acetate (9:1) to give 89 mg (14 %) **32** as a pale orange solid.

**Mpt:** 71 °C. **^1^H-NMR (500 MHz, CDCl_3_):** δ (ppm) = 8.96 (bs, 1H, 2´´´´-NH), 8.20 (d, *J* = 8.2 Hz, 1H, 3´´´´-H), 7.90 (d, *J* = 8.2 Hz, 1H, 6´-H), 7.55 (d, *J* = 8.1 Hz, 1H, 6´´´-H), 7.43 – 7.34 (m, 2H, 5´´´-H, 4´´´´-H), 7.34 – 7.28 (m, 3H, 3´-H, 4´-H, 6´´´´-H), 7.25 – 7.17 (m, 3H, 3´´´-H, 4´´´-H, 5´´´´-H), 7.09 (bs, 1H, 1´´-H), 6.78 – 6.73 (m, 1H, 5´-H), 3.73 (s, 3H, O-CH_3_), 1.81 (s, 3H, C-CH_3_). **^13^C-NMR (126 MHz, CDCl_3_):** δ (ppm) = 168.24 (C-1), 168.22 (C-CH_3_), 146.72 (C-2´), 139.10 (C-2´´´´), 135.56 (C-1´´´´), 133.95 (C-4´), 132.18 (C-6´, C-6´´´´), 131.16 (C-2´´´), 130.85 (C-3´´´), 129.20 (C-1´´´), 129.17 (C-4´´´´), 128.92 (C-5´´´), 124.67 (C-5´´´´), 124.03 (C-4´´´), 121.95 (C-6´´´), 121.64 (C-3´´´´), 118.24 (C-5´), 113.88 (C-3´), 113.60 (C-1´), 51.93 (O-CH_3_), 24.55 (C-CH_3_). **MS (EI):** m/z (rel. int. in %) = 360 (100) [M^+.^], 301 (45), 285 (60), 167 (50). **HR-MS (EI):** *m/z* found 360.1466, calcd. for C_22_H_20_N_2_O_3_: 360.1474.

***N*-[2-(9-Oxo-9,10-dihydroacridin-4-yl)phenyl]acetamid (33)**

178 mg (0.494 mmol) **32** and 63 mg (1.5 mmol) lithium hydroxide monohydrate were dissolved in a mixture of 2 mL methanol, 2 mL THF and 1 mL water, and refluxed for 3h. The mixture was evaporated to dryness and the residue taken up in 10 mL water and acidified with hydrochloric acid. The precipitated carboxylic acid of **32** was collected by filtration, dried, and treated with 3 g polyphosphoric acid at 165 °C for 2 h. The mixture was treated with crushed ice, brought to pH 3-4 with sodium hydroxide, and extracted with dichloromethane (3 x 40 mL) to give, after drying over Na_2_SO_4_ and evaporation, a mixture of the target compound and its N-deacetylated analogue. The residue was dissolved in 3 mL dichloromethane and treated at 0 °C with 120 µL (1.49 mmol) pyridine and 100 µL (1.06 mmol) acetanhydride for 2h. The solution was washed with satd. sodium bicarbonate solution, dried over Na_2_SO_4_ and evaporated. The residue was purified by silica gel chromatography using dichloromethane/ethyl acetate (9:1) to give 91 mg (56 %) **33** as a pale yellow solid.

**Mpt:** 313 °C. **^1^H-NMR (400 MHz, DMSO-d_6_):** δ (ppm) = 9.91 (bs, 1H, 10´´-H), 9.15 (bs, 1H, 1-NH), 8.31 (dd, *J* = 8.0, 1.6 Hz, 1H, 1´´-H), 8.23 (dd, *J* = 8.1, 1.6 Hz, 1H, 8´´-H), 7.76 – 7.68 (m, 2H, 3´-H, 5´´-H), 7.65 (ddd, *J* = 8.5, 6.8, 1.6 Hz, 1H, 6´´-H), 7.50 (ddd, *J* = 8.2, 6.4, 2.4 Hz, 1H, 5´-H), 7.45 (dd, *J* = 7.2, 1.6 Hz, 1H, 3´´-H), 7.40 – 7.34 (m, 2H, 4´-H, 6´-H), 7.32 (dd, *J* = 8.0, 7.2 Hz, 1H, 2´´-H), 7.24 (ddd, *J* = 8.1, 6.8, 1.2 Hz, 1H, 7´´-H), 1.69 (s, 3H, 2-H). **^13^C-NMR (101 MHz, DMSO-d_6_):** δ (ppm) = 177.00 (C-9´´), 168.84 (C-1), 140.92 (C-10´´a), 138.71 (C-4´´a), 136.46 (C-2´), 135.03 (C-3´´), 133.10 (C-6´´), 131.41 (C-6´), 131.21 (C-1´), 128.74 (C-5´), 127.53 (C-9´´a), 126.56 (C-3´), 125.77 (C-1´´, C-8´´), 125.62 (C-4´), 121.28 (C-4´´), 121.21 (C-7´´), 120.80 (C-2´´), 120.22 (C-8´´a), 118.38 (C-5´´), 22.91 (C-2). **MS (EI):** m/z (rel. int. in %) = 328 (15) [M^+.^], 286 (100), 268 (40), 162 (25). **HR-MS (EI):** *m/z* found 328.1220, calcd. for C_21_H_16_N_2_O_2_: 328.1212.

**8-Bromo-1,2-dichloro-9*H*-carbazole (34)**

A suspension of 200 mg (0.787 mmol) 7,8-dichloro-2,3,4,9-tetrahydro-1*H*-carbazol-1-one dissolved in 12 mL anisole was treated with 1.4 g (4.9 mmol) POBr_3_ and heated at 120 °C for 1 h. After cooling to ambient temperature 70 mL water was added, the mixture was neutralised with satd. sodium bicarbonate solution, and extracted with ethyl acetate (3 x 50 mL). The combined organic layers were dried over Na_2_SO_4_ and evaporated. The residue was purified by silica gel chromatography using dichloromethane/hexane (1:1) to give 125 mg (50 %) **34** as a beige solid.

**Mpt:** 147 °C. **^1^H-NMR (500 MHz, CD_2_Cl_2_):** δ (ppm) = 8.50 (bs, 1H, 9-H), 7.98 (ddd, *J* = 7.9, 0.8 Hz, 1H, 5-H), 7.88 (dd, *J* = 8.4, 0.7 Hz, 1H, 4-H), 7.63 (dd, *J* = 7.8, 0.9 Hz, 1H, 7-H), 7.35 (d, *J* = 8.4 Hz, 1H, 6-H), 7.18 (t, *J* = 7.8 Hz, 1H, 3-H). **^13^C-NMR (101 MHz, CD_2_Cl_2_):** δ (ppm) = 138.85 (C-8a), 137.93 (C-9a), 130.26 (C-2), 129.54 (C-7), 124.96 (C-4b), 123.91 (C-4a), 122.45 (C-3), 122.26 (C-6), 120.30 (C-5), 120.25 (C-4), 115.50 (C-1), 104.96 (C-8). **MS (EI):** m/z (rel. int. in %) = 317 (50) [M^+.^], 315 (100) [M^+.^], 313 (70) [M^+.^], 280 (20), 252 (40), 234 (50), 198 (40), 162 (40). **HR-MS (EI):** *m/z* found 312.9067, calcd. for C_12_H_6_BrCl_2_N: 312.9061.

**1-Bromo-6-chloro-9*H*-carbazole (35)**

Prepared in the same manner as described for **34** from 300 mg (1.37 mmol) 6-chloro-2,3,4,9-tetrahydro-1*H*-carbazol-1-one and 2.4 g (8.4 mmol) POBr_3_ in 15 ml anisole. **Yield:** 106 mg (28 %), pale yellow solid.

**Mpt:** 117 °C. **^1^H-NMR (400 MHz, CD_2_Cl_2_):** δ (ppm) = 8.41 (bs, 1H, 9-H), 8.02 (ddd, *J* = 1.8, 0.8 Hz, 1H, 5-H), 7.98 (dt, *J* = 7.8, 0.9 Hz, 1H, 4-H), 7.61 (dd, *J* = 7.8, 1.0 Hz, 1H, 2-H), 7.48 – 7.40 (m, 2H, 7-H, 8-H), 7.14 (t, *J* = 7.8 Hz, 1H, 3-H). **^13^C-NMR (101 MHz, CD_2_Cl_2_):** δ (ppm) = 139.25 (C-9a), 138.05 (C-8a), 129.24 (C-2), 127.16 (C-7), 125.96 (C-6), 125.30 (C-4b), 124.17 (C-4a), 121.46 (C-3), 121.00 (C-5), 120.10 (C-4), 112.76 (C-8), 104.70 (C-1). **MS (ESI):** m/z (rel. int. in %) = 282 (33) (M - H)^-^, 280 (100) (M - H)^-^, 278 (85) (M - H)^-^. **HR-MS (ESI):** *m/z* found 277.9377, calcd. for C_12_H_6_BrClN^-^ [M - H]^-^: 277.9372.

**1-Bromo-6-methoxy-9*H*-carbazole (36)**

Prepared in the same manner as described for **34** from 300 mg (1.37 mmol) 6-methoxy-2,3,4,9-tetrahydro-1*H*-carbazol-1-one and 3.5 g (12 mmol) POBr_3_ in 30 ml anisole. **Yield:** 372 mg (29 %), beige solid.

**Mpt:** 182 °C. **^1^H-NMR (400 MHz, CD_2_Cl_2_):** δ (ppm) = 8.26 (bs, 1H, 9-H), 7.99 (ddd, *J* = 7.8, 0.8 Hz, 1H, 4-H), 7.56 (dd, *J* = 7.7, 0.9 Hz, 1H, 2-H), 7.54 (d, *J* = 2.6 Hz, 1H, 5-H), 7.43 (dd, *J* = 8.8, 0.6 Hz, 1H, 8-H), 7.13 – 7.08 (m, 2H, 3-H, 7-H), 3.91 (s, 3H, CH_3_). **^13^C-NMR (101 MHz, CD_2_Cl_2_):** δ (ppm) = 154.94 (C-6), 139.38 (C-9a), 134.56 (C-8a), 128.41 (C-2), 125.08 (C-4a), 124.56 (C-4b), 120.67 (C-3), 119.83 (C-4), 116.46 (C-7), 112.39 (C-8), 104.61 (C-1), 103.81 (C-5), 56.44 (CH_3_). **MS (EI):** m/z (rel. int. in %) = 277 (95) [M^+.^], 275 (100) [M^+.^], 262 (75), 260 (80), 234 (20), 232 (20). **HR-MS (EI):** *m/z* found 274.9948, calcd. for C_13_H_10_BrNO: 274.9946.

***N*-[2-(7,8-Dichloro-9*H*-carbazol-1-yl)phenyl]acetamide (37)**

A solution of 150 mg (0.476 mmol) **34**, 86 mg (0.48 mmol) 2-acetamidophenylboronic acid and 56 mg (0.046 mmol) Pd(Ph_3_P)_4_ in 10 mL 1,2-dimethoxyethane and 5 mL ethanol under N_2_ was stirred for 10 min, then treated with 4 mL of a nitrogen-purged 2M sodium carbonate solution, and heated at 90 °C for 15 h. After addition of 100 mL water the mixture was extracted with diethyl ether (4 x 60 mL). The combined organic layers were dried over Na_2_SO_4_ and evaporated. The residue was purified by silica gel chromatography using ethyl acetate/hexane (1:1) to give 20 mg (11 %) **37** as a white solid.

**Mpt:** 233 °C. **^1^H-NMR (400 MHz, DMSO-d_6_):** δ (ppm) = 10.64 (bs, 1H, 9´´-H), 9.06 (bs, 1H, 1-NH), 8.23 – 8.12 (m, 2H, 4´´-H, 5´´-H), 7.69 (d, *J* = 7.4 Hz, 1H, 6´-H), 7.49 – 7.37 (m, 3H, 3´-H, 5´-H, 3´´-H), 7.37 – 7.27 (m, 3H, 4´-H, 2´´-H, 6´´-H), 1.74 (s, 3H, 2-H). **^13^C-NMR (101 MHz, DMSO-d_6_):** δ (ppm) = 169.19 (C-1), 138.49 (C-8´´a), 137.96 (C-9´´a), 135.89 (C-1´), 132.44 (C-2´), 131.07 (C-5´), 128.09 (C-3´, C-2´´), 127.86 (C7´´), 126.34 (C-6´), 125.56 (C-4´), 123.39 (C-4´´a), 123.24 (C-4´´b), 123.14 (C-8´´), 120.61 (C-3´´), 120.29 (C-6´´), 120.04 (C-5´´), 119.79 (C-4´´), 113.88 (C-1´´), 23.08 (C-2). **MS (EI):** m/z (rel. int. in %) = 370 (50) [M^+.^], 369 (10) [M^+.^], 368 (60) [M^+.^], 326 (100), 327 (35), 328 (55), 277 (55), 278 (20), 159 (20). **HR-MS (EI):** *m/z* found 368.0481, calcd. for C_20_H_14_Cl_2_N_2_O: 368.0483.

***N*-[2-(6-Chloro-9*H*-carbazol-1-yl)phenyl]acetamide (38)**

Prepared in the same manner as described for **37** from 120 mg (0.428 mmol) **35** and 77 mg (0.43 mmol) 2-acetamidophenylboronic acid.

**Yield:** 15 mg (10 %), beige solid, **Mpt:** >250 °C. **^1^H-NMR (500 MHz, CD_2_Cl_2_):** δ (ppm) = 8.29 (bs, 1H, 9´´-H), 8.22 (d, *J* = 8.3 Hz, 1H, 6´-H), 8.11 (dd, *J* = 7.6, 1.0 Hz, 1H, 4´´-H), 8.09 (d, *J* = 1.9 Hz, 1H, 5´´-H), 7.47 (ddd, *J* = 8.6, 7.5, 1.7 Hz, 1H, 5´-H), 7.44 (dd, *J* = 7.6, 1.7 Hz, 1H, 3´-H), 7.42 – 7.33 (m, 4H, 2´´-H, 3´´-H, 7´´-H, 8´´-H), 7.31 (ddd, *J* = 7.5, 1.3 Hz, 1H, 4´-H), 7.03 (bs, 1H, 1-NH), 1.78 (s, 3H, 2-H). **^13^C-NMR (126 MHz, CD_2_Cl_2_):** δ (ppm) = 169.47 (C-1), 139.04 (C-9´´a), 138.52 (C-8´´a), 136.20 (C-1´), 131.16 (C-3´), 129.51 (C-5´), 127.89 (C-2´´, C-4´´a), 126.81 (C-7´´), 125.57 (C-4´), 125.55 (C-2´), 124.99 (C-4´´b), 123.49 (C-6´), 123.41 (C-6´´), 121.45 (C-1´´), 121.04 (C-4´´), 120.78 (C-5´´), 120.64 (C-3´´), 112.52 (C-8´´), 24.67 (C-2). **MS (EI):** m/z (rel. int. in %) = 336 (40) [M^+.^], 335 (30) [M^+.^], 334 (90) [M^+.^], 292 (100), 293 (25), 294 (40), 255 (20), 256 (60), 257 (20). **HR-MS (EI):** *m/z* found 334.0871, calcd. for C_20_H_15_ClN_2_O: 334.0873.

***N*-[2-(6-Methoxy-9*H*-carbazol-1-yl)phenyl]acetamide (39)**

Prepared in the same manner as described for **37** from 50 mg (0.18 mmol) **36** and 32 mg (0.18 mmol) 2-acetamidophenylboronic acid. Final purification was performed by crystallisation from pentane.

**Yield:** 30 mg (25 %), white solid, **Mpt:** 224 °C. **^1^H-NMR (500 MHz, acetone-d_6_):** δ (ppm) = 9.80 (bs, 1H, 9´´-H), 8.25 (d, *J* = 8.2 Hz, 1H, 6´-H), 8.15 (bs, 1H, 1-NH), 8.13 (ddd, *J* = 7.6, 1.4, 0.7 Hz, 1H, 4´´-H), 7.72 (d, *J* = 2.5 Hz, 1H, 5´´-H), 7.45 – 7.38 (m, 3H, 3´-H, 5´-H, 8´´-H), 7.30 – 7.21 (m, 3H, 4´-H, 2´´-H, 3´´-H), 7.04 (dd, *J* = 8.8, 2.5 Hz, 1H, 7´´-H), 3.90 (s, 3H, O-CH_3_), 1.80 (s, 3H, 2-H). **^13^C-NMR (126 MHz, acetone-d_6_):** δ (ppm) = 169.21 (C-1), 154.92 (C-6´´), 139.77 (C-9´´a), 137.50 (C-1´), 136.11 (C-8´´a), 131.54 (C-5´), 129.44 (C-2´), 128.99 (C-3´), 127.45 (C-2´´), 125.07 (C-4´), 124.66 (C-4´´a), 124.46 (C-4´´b), 123.81 (C-6´), 122.20 (C-1´´), 120.76 (C-4´´), 119.63 (C-3´´), 116.08 (C-7´´), 112.78 (C-8´´), 103.62 (C-5´´), 56.12 (O-CH_3_), 24.06 (C-2). **MS (ESI):** m/z (rel. int. in %) = 331 (100) (M + H)^+^, 289 (15). **HR-MS (ESI):** *m/z* found 331.1441, calcd. for C_21_H_19_N_2_O_2_^+^ [M + H]^+^: 331.1447.

**3-Bromo-*N*-(2-bromophenyl)pyridin-2-amine (41)**

38 mg (0.17 mmol) Pd(OAc)_2_ and 98 mg (0.17 mmol) Xantphos were suspended in 10 mL anisole and stirred for 10 min. Then 600 mg (3.47 mmol) 2-amino-3-bromopyridine, 450 µL (3.50 mmol) 1-bromo-2-iodobenzene and 1.6 g (4.9 mmol) cesium carbonate wee added. The mixture was stirred at ambient temperature for 15 min, then heated at 130 °C for 12 h. After cooling to ambient temperature 12 mL water was added, and the organic layer was separated, washed with water, dreid over Na_2_SO_4_, and evaporated. The residue was purified by silica gel chromatography using hexane/dichloromethane (9:1) to give 756 mg (66 %) **41** as a white solid.

**Mpt:** 83 °C. **^1^H-NMR (400 MHz, CD_2_Cl_2_):** δ (ppm) = 8.60 (dd, *J* = 8.3, 1.6 Hz, 1H, 6´-H), 8.18 (dd, *J* = 4.8, 1.6 Hz, 1H, 6-H), 7.82 (dd, *J* = 7.8, 1.6 Hz, 1H, 4-H), 7.71 (bs, 1H, 2-NH), 7.59 (dd, *J* = 8.0, 1.5 Hz, 1H, 3´-H), 7.38 – 7.28 (m, 1H, 5´-H), 6.91 (ddd, *J* = 8.0, 7.3, 1.6 Hz, 1H, 4´-H), 6.73 (dd, *J* = 7.8, 4.8 Hz, 1H, 5-H). **^13^C-NMR (101 MHz, CD_2_Cl_2_):** δ (ppm) = 152.03 (C-2), 146.76 (C-6), 141.02 (C-4), 138.52 (C-1´), 132.87 (C-3´), 128.49 (C-5´), 123.64 (C-4´), 120.92 (C-6´), 117.03 (C-5), 114.13 (C-2´), 107.82 (C-3). **MS (ESI):** m/z (rel. int. in %) = 327 (40) (M + H)^+^, 329 (100) (M + H)^+^, 331 (45) (M + H)^+^, 247 (65), 249 (65). **HR-MS (ESI):** *m/z* found 326.9129, calcd. for C_11_H_9_Br_2_N_2_^+^ [M + H]^+^: 326.9132.

***N*-[2-(9H-Pyrido[2,3-b]indol-8-yl)phenyl]acetamide (43)**

940 mg (2.87 mmol) **41**, 129 mg (0.575 mmol) Pd(OAc)_2_, and 201 mg (0.573 mmol) CyJohnPhos were disperged in 5 mL *N*,*N*-dimethylacetamide and stirred for 10 min. Then 860 µL (5.76 mmol) DBU was added and the mixture heated at 130 °C for 16 h. After cooling 5 mL water, 20 mL ethyl acetate, and 5 mL THF was added. The organic layer was separated, dried over Na_2_SO_4_, and evaporated. The residue was purified by silica gel chromatography using hexane/dichloromethane (1:1) to give intermediate 8-bromo-9*H*-pyrido[2,3-b]indole (**42**). A solution of 313 mg (1.27 mmol) **42**, 269 mg (1.50 mmol) 2-acetamidophenylboronic acid and 150 mg (0.130 mmol) Pd(Ph_3_P)_4_ in a degassed mixture 15 mL 1,2-dimethoxyethane und 8 mL ethanol was stirred for 10 min, then 10 mL nitrogen-purged 2M sodium carbonate solution was added and the mixture heated to 90 °C for 12 h. After cooling 100 mL water was added, followed by extraction with dichloromethane (4 x 60 mL). The combined organic layers were dried over Na_2_SO_4_ and evaporated. The residue was purified by silica gel chromatography using dichloromethane/ethyl acetate (9:1 to 1:9) to give 26 mg (7 %) **43** as a reddish-brown solid.

**Mpt:** >250 °C, **^1^H-NMR (500 MHz, DMSO-d_6_):** δ (ppm) = 11.29 (bs, 1H, 9´´-H), 9.01 (bs, 1H, 1-NH), 8.54 (dd, *J* = 7.7, 1.7 Hz, 1H, 4´´-H), 8.40 (dd, *J* = 4.9, 1.6 Hz, 1H, 2´´-H), 8.18 (dd, *J* = 6.2, 2.8 Hz, 1H, 5´´-H), 7.72 (d, *J* = 8.0 Hz, 1H, 6´-H), 7.48 – 7.38 (m, 2H, 3´-H, 5´-H), 7.35 – 7.25 (m, 3H, 4´-H, 6´´-H, 7´´-H), 7.21 (dd, *J* = 7.7, 4.8 Hz, 1H, 3´´-H), 1.75 (s, 3H, 2-H). **^13^C-NMR (126 MHz, DMSO-d_6_):** δ (ppm) = 168.67 (C-1), 152.34 (C-9´´a), 146.20 (C-2´´), 137.01 (C-8´´a), 135.82 (C-1´), 132.27 (C-2´), 130.85 (C-3´), 128.25 (C-4´´), 127.92 (C-5´), 127.58 (C-7´´), 125.98 (C-6´), 125.41 (C-4´), 122.48 (C-8´´), 121.22 (C-4´´b), 120.29 (C-5´´), 119.48 (C-6´´), 115.36 (C-4´´a), 115.07 (C-3´´), 23.17 (C-2). **MS (EI):** m/z (rel. int. in %) = 301 (80) [M^+.^], 258 (100), 170 (65). **HR-MS (EI):** *m/z* found 301.1217, calcd. for C_19_H_15_N_3_O: 301.1215.

***N*-[2-(5,6,7,8-Tetrahydro-9*H*-carbazol-1-yl)phenyl]acetamide (44)**

Prepared from 143 mg (0.572 mmol) 8-bromo-1,2,3,4-tetrahydro-1*H*-carbazole and 102 mg (0.570 mmol) 2-acetamidophenylboronic acid using 66 mg (0.057 mmol) Pd(Ph_3_P)_4_ following the General Procedure for the “First generation 1-(heteroaryl)carbazoles (**3-14**) prepared by Suzuki-Miyaura cross-coupling of with (hetero)arylboronic acids“. Microwave conditions: 80 °C, 150 Watt, 15 min. Purification by silica gel chromatography using dichloromethane/hexane (4:1) gave 70 mg (40 %) **44** as a pale yellow solid.

**Mpt:** 185 °C. **^1^H-NMR (400 MHz, CD_2_Cl_2_):** δ (ppm) = 8.29 (d, *J* = 8.2 Hz, 1H, 6´-H), 7.74 (bs, 1H, 1-NH), 7.50 (d, *J* = 7.8 Hz, 1H, 5´´-H), 7.44 – 7.35 (m, 2H, 3´-H, 5´-H), 7.23 (td, *J* = 7.5, 1.3 Hz, 1H, 4´-H), 7.20 – 7.13 (m, 2H, 6´´-H, 9´´-H), 7.03 (dd, *J* = 7.3, 1.1 Hz, 1H, 7´´-H), 2.72 (t, *J* = 4.9 Hz, 2H, 3´´-H), 2.66 (t, *J* = 5.5 Hz, 2H, 2´´-H), 1.94 – 1.85 (m, 4H, 4´´-H, 1´´-H), 1.85 (s, 3H, 2-H). **^13^C-NMR (126 MHz, CD_2_Cl_2_):** δ (ppm) = 169.12 (C-1), 136.24 (C-1´), 135.71 (C-9´´a), 134.41 (C-8´´a), 130.88 (C-3´), 129.55 (C-2´), 129.01 (C-4´´b), 128.94 (C-5´), 124.89 (C-4´), 122.34 (C-6´), 122.04 (C-7´´), 120.51 (C-8´´), 119.95 (C-6´´), 118.30 (C-5´´), 110.93 (C-4´´a), 24.83 (C-2), 23.79 (C-4´´), 23.66 (C-1´´), 23.59 (C-2´´), 21.46 (C-3´´). **MS (EI):** m/z (rel. int. in %) = 304 (100) [M^+.^], 261 (51), 234 (67), 219 (18), 43 (19). **HR-MS (EI):** *m/z* found 304.1564, calcd. for C_20_H_20_N_2_O: 304.1576.

***N*-(2-(3-Isopropyl-1*H*-indol-7-yl)phenyl)acetamide (45)**

Prepared from 400 mg (1.68 mmol) 7-bromo-3-isopropyl-1*H*-indole and 301 mg (1.68 mmol)) 2-acetamidophenylboronic acid using 194 mg (0.168 mmol) Pd(PPh_3_)_4_ following the General Procedure for the “First generation 1-(heteroaryl)carbazoles (**3-14**) prepared by Suzuki-Miyaura cross-coupling of with (hetero)arylboronic acids“ at 90 °C over 12 h (without microwave irradiation). The residue was purified by silica gel chromatography using dichloromethane/hexane (9:1) and hexane/ethyl acetate (9:1) to give 98 mg (20 %) **45** as a white solid.

**Mpt:** 185 °C. **^1^H-NMR (500 MHz, CD_2_Cl_2_):** δ (ppm) = 8.25 (d, *J* = 8.2 Hz, 1H, 6´-H), 8.03 (bs, 1H, 1´´-H), 7.71 (ddd, *J* = 7.9, 0.9 Hz, 1H, 6´´-H), 7.41 (ddd, *J* = 8.5, 7.6, 1.7 Hz, 1H, 5´-H), 7.38 (dd, *J* = 7.6, 1.6 Hz, 1H, 3´-H), 7.27 – 7.18 (m, 2H, 4´-H, 5´´-H), 7.15 (bs, 1H, 1-NH), 7.11 (dd, *J* = 7.2, 1.1 Hz, 1H, 4´´-H), 6.97 (d, *J* = 2.2 Hz, 1H, 2´´-H), 3.24 (septd, *J* = 6.9, 0.9 Hz, 1H, 1´´´-H), 1.82 (s, 3H, 2-H), 1.38 (d, *J* = 6.9 Hz, 6H, 2´´´-H, 1´´´´-H). **^13^C-NMR (126 MHz, CD_2_Cl_2_):** δ (ppm) = 169.20 (C-1), 136.21 (C-1´), 135.33 (C-7´´a), 130.94 (C-3´), 129.53 (C-2´), 129.03 (C-5´), 127.97 (C-3´´a), 125.05 (C-4´), 124.79 (C-3´´), 123.05 (C-4´´), 122.67 (C-6´), 121.46 (C-7´´), 120.66 (C-2´´), 120.02 (C-6´´), 119.91 (C-5´´), 26.09 (C-1´´´), 24.76 (C-2), 23.67 (C-2´´´, C-1´´´´). **MS (EI):** m/z (rel. int. in %) = 292 (49) [M^+.^], 277 (41), 235 (100). **HR-MS (EI):** *m/z* found 292.1564, calcd. for C_19_H_20_N_2_O: 292.1576

***tert*-Butyl 6-bromo-1,3,4,5-tetrahydro-2*H*-pyrido[4,3-*b*]indole-2-carboxylate (46b)**

A suspension of 100 mg (0.398 mmol) 6-bromo-2,3,4,5-tetrahydro-1*H*-pyrido[4,3-*b*]indole (**46**) and 81 µL (1.0 mmol) pyridine in 3 mL dichloromethane was cooled to 0 °C, treated with 218 mg (0.999 mmol) di-*tert*-butyl dicarbonate, and stirred at ambient temperature for 12 h. Then 10 mL water was added, followed by extraction with ethyl acetate (3 x 20 mL). The combined organic layers were dried over Na_2_SO_4_ and evaporated. The residue was purified by silica gel chromatography using dichloromethane/ethyl acetate/hexane (1:1:2) to give 137 mg (98 %) **46b** as a pale yellow solid.

**Mpt:** 79 °C. **^1^H-NMR (500 MHz, CDCl_3_):** δ (ppm) = 8.29 (bs, 1H, 5´-H), 7.48 – 7.32 (m, 1H, 7´-H), 7.29 (d, *J* = 7.6 Hz, 1H, 9´-H), 6.97 (t, *J* = 7.7 Hz, 1H, 8´-H), 4.63 (s, 2H, 1´-H), 3.91 – 3.72 (m, 2H, 4´-H), 2.85 (dd, *J* = 5.7 Hz, 2H, 3´-H), 1.52 (s, 9H, C(CH_3_)_3_). **^13^C-NMR (101 MHz, CDCl_3_):** δ (ppm) = 155.28 (C-1), 134.67 (C-5´a), 133.24 (C-4´a), 126.87 (C-9´a), 123.96 (C-9´), 120.80 (C-8´), 116.88 (C-7´), 108.76 (C-9´b), 104.39 (C-6´), 80.16 (C(CH_3_)_3_), 41.47 (C-4´), 40.69 (C-1´), 28.61 (C(CH_3_)_3_), 23.64 (C-3´). **MS (EI):** m/z (rel. int. in %) = 350 (2) [M^+.^], 352 (1) [M^+.^], 293 (85), 249 (35), 223 (100). **HR-MS (EI):** *m/z* found 350.0626, calcd. for C_16_H_19_BrN_2_O_2_: 350.0630.

***N*-[2-(2,3,4,5-Tetrahydro-1*H*-pyrido[4,3-*b*]indol-6-yl)phenyl]acetamide hydrochloride (47)**

Prepared from 530 mg (1.51 mmol) **46b**, and 270 mg (1.51 mmol) 2-acetamidophenylboronic acid using 175 mg (0.151 mmol) Pd(PPh_3_)_4_ following the General Procedure for the “First generation 1-(heteroaryl)carbazoles (**3-14**) prepared by Suzuki-Miyaura cross-coupling of with (hetero)arylboronic acids“ at 70 °C over 12 h (without microwave irradiation). The intermediate obtained by purification by silica gel chromatography using ethyl acetate/hexane (1:1) was dissolved in 5 mL dichloromethane and 2 mL trifluoroacetic acid and stirred for 12 h. After neutralisation with sodium bicarbonate solution the mixture was extracted with dichloromethane (2 x 15 mL). The combined organic layers were dried over Na_2_SO_4_ and evaporated. The residue was purified by silica gel chromatography using acetone/triethylamine (97:3) and precipitated from dichloromethane with hydrochloric acid (4M in dioxane) to give 91 mg (20 %) **47** as a non-crystallizing yellow oil.

**^1^H-NMR (400 MHz, DMSO-d_6_):** δ (ppm) = 10.69 (bs, 1H, 5´´-H), 9.65 (bs, 2H, 2´´-H), 8.97 (bs, 1H, 1-NH), 7.69 (d, *J* = 7.9 Hz, 1H, 6´-H), 7.45 (d, *J* = 7.7 Hz, 1H, 9´´-H), 7.42 – 7.36 (m, 2H, 3´-H, 5´-H), 7.34 – 7.27 (m, 1H, 4´-H), 7.07 (t, *J* = 7.5 Hz, 1H, 8´´-H), 6.96 (d, *J* = 7.2 Hz, 1H, 7´´-H), 4.30 (s, 2H, 1´´-H), 3.48 – 3.35 (m, 2H, 3´´-H), 2.98 (t, *J* = 5.9 Hz, 2H, 4´´-H), 1.78 (s, 3H, 2-H). **^13^C-NMR (101 MHz, DMSO-d_6_):** δ (ppm) = 168.55 (C-1), 135.76 (C-1´), 133.94 (C-5´´a), 132.72 (C-2´), 131.47 (C-4´´a), 130.42 (C-5´), 127.76 (C-3´), 126.22 (C-6´), 125.78 (C-6´´), 125.37 (C-4´), 122.18 (C-7´´), 122.06 (C-9´´a), 119.03 (C-8´´), 116.90 (C-9´´), 102.09 (C-9´´b), 40.76 (C-3´´), 39.91 (C-1´´), 23.16 (C-2), 20.19 (C-4´´). **MS (EI)** (free amine)**:** m/z (rel. int. in %) = 305 (45) [M^+.^], 276 (85), 234 (100). **HR-MS (EI)** (free amine)**:** *m/z* found 305.1529, calcd. for C_19_H_19_N_3_O: 305.1528.

**2-(9*H*-Carbazol-1-yl)aniline (48)**

Prepared from 710 mg (2.89 mmol) 1-bromo-9*H*-carbazole and 500 mg (2.88 mmol) 2-aminophenylboronic acid using 333 mg (0.288 mmol) Pd(PPh_3_)_4_ following the General Procedure for the “First generation 1-(heteroaryl)carbazoles (**3-14**) prepared by Suzuki-Miyaura cross-coupling of with (hetero)arylboronic acids“ at 90 °C over 12 h (without microwave irradiation). The residue was purified by silica gel chromatography using hexane/dichloromethane (7:3) to give 598 mg (80 %) **48** as a pale yellow solid.

**Mpt:** 173 °C. **^1^H-NMR (500 MHz, CD_2_Cl_2_):** δ (ppm) = 8.41 (bs, 1H, 9´-H), 8.13 – 8.09 (m, 2H, 4´-H, 5´-H), 7.46 – 7.40 (m, 3H, 2´-H, 7´-H, 8´-H), 7.34 (t, *J* = 7.6 Hz, 1H, 6´-H), 7.31 (dd, *J* = 8.0, 1.6 Hz, 1H, 3-H), 7.29 – 7.22 (m, 2H, 5-H, 3´-H), 6.96 – 6.86 (m, 2H, 4-H, 6-H), 3.77 (s, 2H, 1-NH_2_). **^13^C-NMR (126 MHz, CD_2_Cl_2_):** δ (ppm) = 144.49 (C-1), 140.29 (C-8´a), 138.20 (C-9´a), 131.68 (C-3), 129.53 (C-3´), 127.36 (C-7´), 126.51 (C-2´), 124.54 (C-2), 124.06 (C-4´a), 123.94 (C-4´b), 122.74 (C-1´), 120.83 (C-5´), 120.37 (C-6´), 120.03 (C-5), 119.97 (C-4´), 119.33 (C-4), 116.34 (C-6), 111.40 (C-8´). **MS (ESI):** m/z (rel. int. in %) = 259 (100) (M + H)^+^. **HR-MS (ESI):** *m/z* found 259.1233, calcd. for C_18_H_15_N_2_^+^ [M + H]^+^: 259.1235.

**1-[2-(9*H*-Carbazol-1-yl)phenyl]-3-(*tert*-butyl)urea (49)**

An ice-cooled dispersion of 8 mg (0.2 mmol) sodium hydride (60 % in mineral oil) in 1.5 mL DMSO was stirred under N_2_ for 10 min, then 50 mg (0.19 mmol) **48** was added. The mixture was stirred until it turned red, then 23 µL (0.20 mmol) *tert-*butyl isocyanate was added and the mixture was heated at 55 °C for 12 h. 10 mL water was added, followed by extraction with diethyl ether (3 x 20 mL). The combined organic layers were dried over Na_2_SO_4_ and evaporated. The residue was purified by silica gel chromatography using dichloromethane/hexane (9:1) to give 22 mg (32 %) **49** as a beige solid.

**Mpt:** 162 °C. **^1^H-NMR (500 MHz, CD_2_Cl_2_):** δ (ppm) = 8.42 (bs, 1H, 9´´-H), 8.14 – 8.09 (m, 2H, 4´´-H, 5´´-H), 7.92 (dd, *J* = 8.2, 1.2 Hz, 1H, 6´-H), 7.43 – 7.31 (m, 5H, 3´-H, 5´-H, 2´´-H, 3´´-H, 7´´-H, 8´´-H), 7.24 (ddd, *J* = 8.0, 6.4, 1.7 Hz, 1H, 6´´-H), 7.21 (td, *J* = 7.5, 1.2 Hz, 1H, 4´-H), 5.96 (bs, 1H, 1-H), 4.31 (bs, 1H, 2-H), 1.15 (s, 9H, 2´´´-H, 3´´´-H, 4´´´-H). **^13^C-NMR (126 MHz, CD_2_Cl_2_):** δ (ppm) = 155.42 (C-2), 140.24 (C-8´´a), 138.46 (C-9´´a), 137.41 (C-1´), 131.38 (C-3´), 130.05 (C-2´), 129.27 (C-5´), 127.30 (C-7´´), 126.60 (C-2´´), 124.30 (C-4´), 124.06 (C-4´´a), 123.69 (C-4´´b), 123.66 (C-6´), 121.91 (C-1´´), 120.77 (C-4´´), 120.49 (C-5´´), 120.29 (C-3´´), 120.01 (C-6´´), 111.57 (C-8´´), 51.08 (C-1´´´), 29.36 (C-2´´´, C-3´´´, C-4´´´). **MS (ESI):** m/z (rel. int. in %) = 356 (55) (M - H)^-^, 257 (100). **HR-MS (ESI):** *m/z* found 356.1769, calcd. for C_23_H_22_N_3_O^-^ [M - H]^-^: 356.1763.

***N*-[2-(9*H*-Carbazol-1-yl)phenyl]-1,1,1-trifluorpropan-2-imine (50)**

40 mg (0.039 mmol) Pd_2_(dba)_3_-chloroform adduct, 64 mg (0.12 mmol) 1,1′-bis(diphenylphosphino)ferrocene and 303 mg (0.929 mmol) cesium carbonate in 2 mL toluene under N_2_ were treated under ice-cooling with 200 mg (0.774 mmol) **48** and 96 mg (0.93 mmol) 2-bromo-3,3,3-trifluoro-1-propene. After stirring at 110 °C for 15 h the solid components were removed by filtration, and after evaporation the residue was purified by silica gel chromatography using hexane/ethyl acetate (9:1) to give 182 mg (67 %) **50** as a colorless oil.

**^1^H-NMR (400 MHz, CD_2_Cl_2_):** δ (ppm) = 8.67 (bs, 1H, 9´´-H), 8.12 – 8.08 (m, 2H, 4´´-H, 5´´-H), 7.65 (dd, *J* = 7.7, 1.4 Hz, 1H, 3´-H), 7.51 (ddd, *J* = 7.7, 1.5 Hz, 1H, 5´-H), 7.47 (ddd, *J* = 8.2, 1.4, 0.8 Hz, 1H, 8´´-H), 7.45 – 7.36 (m, 3H, 4´-H, 2´´-H, 7´´-H), 7.31 (t, *J* = 7.6 Hz, 1H, 3´´-H), 7.25 (ddd, *J* = 8.1, 6.8, 1.4 Hz, 1H, 6´´-H), 6.96 (dd, *J* = 7.8, 1.4 Hz, 1H, 6´-H), 1.70 (s, 3H, 3-H). **^13^C-NMR (126 MHz, CD_2_Cl_2_):** δ (ppm) = 158.66 (q, *J* = 33.8 Hz, C-2), 145.46 (C-1´), 140.63 (C-8´´a), 137.48 (C-9´´a), 132.24 (C-3´), 129.22 (C-5´), 129.12 (C-2´), 127.58 (C-7´´), 126.56 (C-2´´), 126.37 (C-4´), 124.45 (C-4´´a), 123.86 (C-4´´b), 122.37 (C-1´´), 120.73 (C-4´´), 120.33 (C-5´´), 120.17 (C-6´´), 119.99 (C-3´´), 119.29 (C-6´), 118.67 (C-1), 111.45 (C-8´´), 15.16 (C-3). **MS (EI):** m/z (rel. int. in %) = 353 (18) [M^+.^], 352 (84) [M^+.^], 284 (21), 283 (100), 241 (31), 142 (25), 121 (28). **HR-MS (EI):** *m/z* found 352.1182, calcd. for C_21_H_15_F_3_N_2_: 352.1187.

**(±)-2-(9*H*-Carbazol-1-yl)-*N*-(1,1,1-trifluoropropan-2-yl)aniline (51)**

A solution of 150 mg (0.426 mmol) **50** in 2 mL THF was added to 50 mg (1.3 mmol) sodium borohydride in 1 mL THF containing 3 drops of methanol. After stirring for 2 h 5 mL ethyl acetate was added and the solid components were removed by filtration. The filtrate was evaporated and the residue purified by silica gel chromatography using hexane/dichloromethane (4:1) to give 108 mg (72 %) **51** as a colorless oil.

**^1^H-NMR (400 MHz, methanol-d_4_, 50 °C):** δ (ppm) = 8.13 – 8.06 (m, 2H, 4´-H, 5´-H), 7.42 (ddd, *J* = 8.2, 1.0 Hz, 1H, 8´-H), 7.37 – 7.30 (m, 2H, 3-H, 7´-H), 7.30 – 7.23 (m, 3H, 5-H, 2´-H, 3´-H), 7.17 (ddd, *J* = 8.0, 7.0, 1.1 Hz, 1H, 6´-H), 6.98 (d, *J* = 8.3 Hz, 1H, 6-H), 6.91 (ddd, *J* = 7.4, 1.1 Hz, 1H, 4-H), 4.15 (bs, 1H, 2´´-H), 1.09 (d, *J* = 6.8 Hz, 3H, 3´´-H). **^13^C-NMR (101 MHz, methanol-d_4_, 50 °C):** δ (ppm) = 145.37 (C-1), 141.75 (C-8´a), 139.45 (C-9´a), 132.08 (C-5), 130.07 (C-2´), 129.29 (C-1´´), 127.73 (C-3), 126.82 (C-7´), 126.44 (C-2), 125.05 (C-4´a), 124.43 (C-4´b), 122.95 (C-1´), 121.01 (C-5´), 120.59 (C-4´), 120.32 (C-3´), 120.03 (C-6´), 119.76 (C-4), 113.76 (C-6), 112.08 (C-8´), 51.52 (q, *J* = 30.7 Hz, C-2´´), 15.10 (C-3´´). **MS (ESI):** m/z (rel. int. in %) = 356 (15) (M + H)^+^, 355 (100) (M + H)^+^. **HR-MS (ESI):** *m/z* found 355.1417, calcd. for C_21_H_18_F_3_N_2_^+^ [M + H]^+^: 355.1422.

***N*-[2-(9*H*-Carbazol-1-yl)phenyl]thioacetamide (52)**

40 mg (0.13 mmol) *N*-(2-(9*H*-carbazol-1-yl)phenyl)acetamide (**1**) and 59 mg (0.15 mmol) Lawesson‘s reagent werde suspended in 1 mL anhydrous THF and heated at 55 °C for 12 h. After adding 20 mL water the mixture was extracted with dichloromethane (3 x 20 mL). The combined organic layers were dried over Na_2_SO_4_ and evaporated. The residue was purified by silica gel chromatography using dichloromethane to give 25 mg (59 %) **52** as a yellow solid.

**Mpt:** 90 °C. **^1^H-NMR (400 MHz, CD_2_Cl_2_):** δ (ppm) = 8.61 (bs, 1H, 9´´-H), 8.39 (bs, 1H, 1-NH), 8.14 (ddd, *J* = 7.7, 1.3, 0.7 Hz, 1H, 4´´-H), 8.10 (ddd, *J* = 7.8, 0.8 Hz, 1H, 5´´-H), 8.00 – 7.96 (m, 1H, 6´-H), 7.60 – 7.56 (m, 1H, 4´-H), 7.56 – 7.47 (m, 2H, 3´-H, 5´-H), 7.43 – 7.36 (m, 3H, 2´´-H, 7´´-H, 8´´-H), 7.33 (t, *J* = 7.6 Hz, 1H, 3´´-H), 7.24 (ddd, *J* = 8.1, 6.4, 1.8 Hz, 1H, 6´´-H), 2.28 (d, *J* = 0.5 Hz, 3H, 2-H). **^13^C-NMR (101 MHz, CD_2_Cl_2_):** δ (ppm) = 203.67 (C-1), 140.09 (C-8´´a), 138.70 (C-9´´a), 137.03 (C-1´), 134.23 (C-2´), 131.93 (C-4´), 128.90 (C-5´), 128.75 (C-3´), 128.69 (C-6´), 127.27 (C-2´´), 126.78 (C-7´´), 123.69 (C-4´´a), 123.34 (C-4´´b), 121.01 (C-1´´), 120.80 (C-4´´), 120.78 (C-5´´), 120.06 (C-6´´), 120.02 (C-3´´), 111.21 (C-8´´), 35.38 (C-2). **MS (EI):** m/z (rel. int. in %) = 316 (10) [M^+.^], 282 (100), 141 (15). **HR-MS (EI):** *m/z* found 316.1036, calcd. for C_20_H_16_N_2_S: 316.1034.

**2-(9*H*-Carbazol-1-yl)-*N*-ethylaniline (53)**

An ice-cooled suspension of 100 mg (0.333 mmol) *N*-(2-(9*H*-carbazol-1-yl)phenyl)acetamide (**1**) in 2 mL toluene was treated with 200 µL (0.400 mmol) borane-dimethyl sulfide complex (2M in THF) and stirred at 0 °C for 15 min and then refluxed for 4 h. After neutralisation with sodium carbonate solution (10%) the mixture was extracted with dichloromethane (3 x 20 mL). The combined organic layers were dried over Na_2_SO_4_ and evaporated. The residue was purified by silica gel chromatography using dichloromethane/hexane (1:1) to give 92 mg (96 %) **53** as a beige solid.

**Mpt:** 130 °C. **^1^H-NMR (500 MHz, CD_2_Cl_2_):** δ (ppm) = 8.30 (bs, 1H, 9´´-H), 8.12 (dd, *J* = 7.8, 0.9 Hz, 1H, 4´´-H), 8.11 (ddd, *J* = 7.8, 0.7 Hz, 1H, 5´´-H), 7.44 – 7.39 (m, 3H, 2´´-H, 7´´-H, 8´´-H), 7.36 – 7.32 (m, 2H, 5´-H, 6´´-H), 7.28 – 7.22 (m, 2H, 3´-H, 3´´-H), 6.87 – 6.81 (m, 2H, 4´-H, 6´-H), 3.68 (bs, 1H, 1-NH)), 3.27 – 3.01 (m, 2H, 1-H), 1.08 (t, *J* = 7.1 Hz, 3H, 2-H). **^13^C-NMR (126 MHz, CD_2_Cl_2_):** δ (ppm) = 146.13 (C-1´), 140.15 (C-9´´a), 138.34 (C-8´´a), 131.43 (C-3´), 129.73 (C-5´), 127.50 (C-2´´), 126.48 (C-7´´), 124.29 (C-2´), 124.01 (C-4´´a), 123.92 (C-4´´b), 122.61 (C-1´´), 120.81 (C-4´´), 120.40 (C-6´´), 120.00 (C-5´´), 119.94 (C-3´´), 117.59 (C-6´), 111.36 (C-8´´), 111.32 (C-4´), 38.92 (C-1), 15.03 (C-2). **MS (EI):** m/z (rel. int. in %) = 286 (100) [M^+.^], 271 (90), 136 (40). **HR-MS (EI):** *m/z* found 286.1469, calcd. for C_20_H_18_N_2_: 286.1470.

***N*-[2-(9*H*-Carbazol-1-yl)phenyl]-*N*-ethylacetamide (54)**

An ice-cooled solution of 100 mg (0.349 mmol) **53** and 32 µL (0.40 mmol) pyridine in 2 mL dichloromethane was treated with 33 µL (0.35 mmol) acetanhydride and stirred at ambient temperature for 2 h. The mixture was washed with satd. sodium carbonate solution and brine, then dried over Na_2_SO_4_ and evaporated. The residue was purified by silica gel chromatography using dichloromethane/hexane (9:1) to give 82 mg (72 %) **54** as a white solid.

**Mpt:** 227 °C. **^1^H-NMR (400 MHz, methanol-d_4_, 50 °C):** δ (ppm) = 8.09 – 8.04 (m, 2H, 4´´-H, 5´´-H), 7.68 – 7.63 (m, 1H, 3´-H), 7.62 – 7.51 (m, 2H, 4´-H, 5´-H), 7.44 – 7.38 (m, 2H, 6´-H, 8´´-H), 7.34 (ddd, *J* = 8.2, 7.1, 1.2 Hz, 1H, 7´´-H), 7.21 (t, *J* = 7.5 Hz, 1H, 3´´-H), 7.19 – 7.13 (m, 2H, 2´´-H, 6´´-H), 3.74 (dq, *J* = 14.0, 7.0 Hz, 1H, 1´´´-H), 2.69 (dq, *J* = 13.9, 7.0 Hz, 1H, 1´´´-H), 1.90 (s, 3H, 2-H), 0.96 (t, *J* = 7.2 Hz, 3H, 2´´´-H). **^13^C-NMR (101 MHz, methanol-d_4_, 50 °C):** δ (ppm) = 172.85 (C-1), 142.59 (C-1´), 141.82 (C-8´´a), 139.51 (C-9´´a), 138.10 (C-2´), 133.35 (C-3´), 131.31 (C-6´), 130.10 (C-4´), 130.01 (C-5´), 126.83 (C-7´´), 126.45 (C-2´´), 125.32 (C-4´´a), 124.43 (C-4´´b), 122.76 (C-1´´), 120.98 (C-4´´), 120.73 (C-5´´), 120.13 (C-6´´), 119.81 (C-3´´), 112.12 (C-8´´), 45.05 (C-1´´´), 22.92 (C-2), 12.89 (C-2´´´). **MS (EI):** m/z (rel. int. in %) = 328 (100) [M^+.^], 271 (85), 131 (55). **HR-MS (EI):** *m/z* found 328.1576, calcd. for C_22_H_20_N_2_O: 328.1576.

**Modifications of the acylamino residue on ring D:**

**General Procedure A: Sulfonamide/carboxamide synthesis**

To a stirred solution of 2-(9H-carbazol-1-yl)aniline (1 eq) in CH_2_Cl_2_ (0.1 M, anhydrous) was added pyridine (1.3 eq) followed by sulfonyl chloride or acid chloride (1.1 eq) and the solution was allowed to stir at room temperature under a nitrogen atmsophere. Upon reaction completion the crude mixture was concentrated onto silica gel and purified by Isolera Biotage LPLC (CH/EA 8:2) to afford pure sulfonamide/amide.

***N*-(2-(9H-Carbazol-1-yl)-5-methylphenyl)methanesulfonamide (55)**

2-(9H-Carbazol-1-yl)-5-methylaniline (50 mg, 0.184 mmol, 1 eq) and methanesulfonyl chloride (16 μL, 0.202 mmol, 1.1 eq) were reacted according to general procedure A to give **55** (44 mg, 0.126 mmol 68%) as a white solid.

**v_max_ (cm^-1^)** 3425, 3246, 1511, 1390, 1317, 1154, 970, 749, 524; **^1^H-NMR (400 MHz, CDCl_3_): δ_H_** 8.1 (t, J=7.70 Hz, 2 H), 7.9 (s, 1 H), 7.6 (s, 1 H), 7.4 (m, 1 H), 7.3 (m, 3 H), 7.2 (m, 2 H), 7.1 (m, 1 H), 6.4 (s, 1 H), 2.7 (s, 3 H), 2.4 (s, 3 H); **^13^C-NMR (100 MHz CDCl_3_): δ_C_** 139.9, 139.6, 137.8, 134.6, 130.8, 126.5, 126.5, 126.4, 126.3, 124.2, 123.4, 121.6, 120.7, 120.6, 120.2, 120.1, 119.6, 111.0, 39.7, 21.5; **LR-ESI-MS**: C_20_H_19_N_2_O_2_S [M+H]^+^ *m/z* found 351.11, cald 351.12.

**3-Amino-4-(9H-carbazol-1-yl)benzonitrile (56b)**

A degassed stirred solution of 1-bromo-9H-carbazole (213 mg, 0.86 mmol, 1 eq), (2-amino-4-cyanophenyl)boronic acid.HCl (189 mg, 0.95 mmol, 1.1 eq), Na_2_CO_3_ (275 mg, 2.60 mmol, 3 eq) and Pd(PPh_3_)_4_ (50 mg, 0.043 mmol, 0.05 eq) in a mixture of 1,4-Dioxane:EtOH:H_2_O (2.5 mL, 2:1:1, 0.5 M) under an inert N_2_ atmosphere was heated with microwave irradiation at 90°C for 2 h. Following completion the crude mixture was concentrated onto silica gel and purified by Isolera Biotage LPLC (CH/EA 8:2) to give **56b** (72 mg, 29 %) as a red solid.

**v_max_ (cm^-1^)** 3477, 3328, 2230, 1614, 1413, 1316, 1237, 752, 619; **^1^H-NMR (400 MHz, DMSO-*d*^6^): δ_H_** 10.79 (s, 1H), 8.02 - 8.33 (m, 2H), 7.50 (d, J = 8.07 Hz, 1H), 7.38 (dt, J = 1.04, 7.61 Hz, 1H), 7.24 - 7.33 (m, 3H), 7.21 (d, J = 1.59 Hz, 1H), 7.13 - 7.19 (m, 1H), 7.09 (dd, J = 1.71, 7.70 Hz, 1H), 5.19 (s, 2H); **^13^C-NMR (100 MHz CDCl_3_): δ_C_** 146.7, 140.1, 137.1, 131.7, 127.3, 126.0, 125.6, 123.1, 122.4, 120.8, 120.1, 120.0, 119.5, 119.2, 118.9, 118.7, 117.3, 111.4, 110.8; **LR-ESI-MS**: C_19_H_14_N_3_ [M+H]^+^ *m/z* found 284.05, cald 284.12.

***N*-(2-(9H-Carbazol-1-yl)-5-cyanophenyl)methanesulfonamide (56)**

3-Amino-4-(9H-carbazol-1-yl)benzonitrile (72 mg, 0.254 mmol, 1 eq) and methanesulfonyl chloride (22 μL, 0.28 mmol, 1.1 eq) were reacted according to general procedure A to give **56** (19 mg, 0.052 mmol 21%) as a white solid.

**v_max_ (cm^-1^)** 3361, 2980, 1588, 1501, 1317, 1032, 750; **^1^H-NMR (400 MHz, CDCl_3_): δ_H_** 8.2 (d, J=7.70 Hz, 1 H), 8.1 (m, 2 H), 8.0 (s, 1 H), 7.6 (d, J=0.86 Hz, 2 H), 7.4 (m, 3 H), 7.3 (m, 2 H), 6.6 (br. s., 1 H), 2.9 (s, 3 H); **^13^C-NMR (100 MHz CDCl_3_): δ_C_** 139.8, 137.1, 136.2, 133.9, 132.3, 128.4, 126.9, 125.8, 124.8, 123.1, 121.8, 120.7, 120.4, 120.4, 117.9, 117.5, 113.3, 111.2, 40.5; **LR-ESI-MS**: C_20_H_16_N_3_O_2_S [M+H]^+^ *m/z* found 362.13, cald 362.09.

***N*-(2-(9*H*-Carbazol-1-yl)-3-methylphenyl)acetamide (57)**

2-(9H-Carbazol-1-yl)-3-methylaniline (43 mg, 0.158 mmol, 1 eq) and acetyl chloride (13 μL, 0.174 mmol, 1.1 eq) were reacted according to general procedure A to give **57** (37 mg, 0.119 mmol 75%) as a white solid.

**v_max_ (cm^-1^)** 3399, 3314, 1680, 1508, 13401, 1335, 1234, 1014, 743; **^1^H-NMR (400 MHz, CDCl_3_): δ_H_** 8.1 (d, J=8.07 Hz, 1 H), 8.1 (dd, J=11.55, 7.76 Hz, 2 H), 7.9 (br. s., 1 H), 7.3 (m, 4 H), 7.2 (m, 2 H), 7.1 (d, J=7.58 Hz, 1 H), 6.8 (br. s., 1 H), 2.0 (m, 3 H), 1.6 (s, 3 H); **^13^C-NMR (100 MHz CDCl_3_): δ_C_** 168.5, 139.5, 137.9, 137.8, 136.1, 128.8, 127.3, 126.5, 126.3, 126.1, 123.9, 123.3, 120.4, 120.0, 119.7, 119.0, 118.7, 110.9, 24.5, 20.3; **LR-ESI-MS**: C_21_H_19_N_2_O [M+H]^+^ *m/z* found 315.31, cald 315.15.

***N*-(2-(9*H*-Carbazol-1-yl)-4-(trifluoromethyl)phenyl)methanesulfonamide (58)**

2-(9H-Carbazol-1-yl)-4-(trifluoromethyl)aniline (82 mg, 0.25 mmol, 1 eq) and methanesulfonyl chloride (21 μL, 0.275 mmol, 1.1 eq) were reacted according to general procedure A to give **58** (11 mg, 0.028 mmol 11%) as a white solid.

**v_max_ (cm^-1^)** 3408, 3328, 1321, 1148, 758, 750, 532; **^19^F-{H}-NMR (376 MHz, DMSO-*d*^6^): δ_F_** –58.8; **^1^H-NMR (400 MHz, CDCl_3_): δ_H_** 8.2 (m, 2 H), 7.9 (m, 2 H), 7.8 (m, 2 H), 7.4 (m, 3 H), 7.3 (m, 2 H), 6.6 (s, 1 H), 2.9 (s, 3 H); **^13^C-NMR (100 MHz CDCl_3_): δ_C_** 139.6, 138.3, 137.3, 128.7, 128.3 (m, J=3.67 Hz), 127.1, 126.8, 126.8, 126.2, 124.7, 123.3, 121.6, 120.7, 120.5, 120.4, 119.0, 117.6, 111.1, 40.3, 26.9; **LR-ESI-MS**: C_20_H_16_F_3_N_2_O­_2_S [M+H]^+^ *m/z* found 405.08, cald 405.09.

**2-(9*H*-Carbazol-1-yl)-5-methylaniline (59)**

A degassed stirred solution of 1-bromo-9H-carbazole (200 mg, 0.81 mmol, 1 eq), (2-amino-4-methylphenyl)boronic acid (135 mg, 0.89 mmol, 1.1 eq), Na_2_CO_3_ (258 mg, 2.44 mmol, 3 eq) and Pd(PPh_3_)_4_ (47 mg, 0.041 mmol, 0.05 eq) in a mixture of 1,4-Dioxane:EtOH:H_2_O (2.2 mL, 2:1:1, 0.5 M) under an inert N_2_ atmosphere was heated with microwave irradiation at 90°C for 2 h. Following completion the crude mixture was concentrated onto silica gel and purified by Isolera Biotage LPLC (CH/EA 8:2) to give **59** (99 mg, 45 %) as an off white solid.

**v_max_ (cm^-1^)** 3401, 3328, 1600, 1570, 1452, 1412, 742; **^1^H-NMR (400 MHz, CDCl_3_): δ_H_** 8.32 (br. s., 1H), 8.12 (t, J = 7.27 Hz, 2H), 7.38 - 7.49 (m, 3H), 7.31 - 7.37 (m, 1H), 7.20 - 7.28 (m, 2H), 6.77 (d, J = 7.70 Hz, 1H), 6.73 (s, 1H), 3.67 (s, 2H), 2.40 (s, 3H); **^13^C-NMR (100 MHz CDCl_3_): δ_C_** 143.5, 139.6, 139.0, 137.7, 131.1, 126.9, 125.9, 123.5, 123.5, 122.0, 121.4, 120.4, 120.0, 119.8, 119.4, 119.4, 116.5, 110.8, 21.3; **LR-ESI-MS**: C_19_H_17_N_2_ [M+H]^+^ *m/z* found 273.09, cald 273.14.

**2-(9*H*-Carbazol-1-yl)-4-(trifluoromethyl)aniline (60)**

A degassed stirred solution of 1-bromo-9H-carbazole (200 mg, 0.81 mmol, 1 eq), (2-amino-5-(trifluoromethyl)phenyl)boronic acid (183 mg, 0.89 mmol, 1.1 eq), Na_2_CO_3_ (258 mg, 2.44 mmol, 3 eq) and Pd(PPh_3_)_4_ (47 mg, 0.041 mmol, 0.05 eq) in a mixture of 1,4-Dioxane:EtOH:H_2_O (2.2 mL, 2:1:1, 0.5 M) under an inert N_2_ atmosphere was heated with microwave irradiation at 90°C for 2 h. Following completion the crude mixture was concentrated onto silica gel and purified by Isolera Biotage LPLC (CH/EA 8:2) to give **60** (102 mg, 38 %) as a brown solid.

**v_max_ (cm^-1^)** 3310, 1659, 1599, 1524, 1454, 1113, 1148, 635, 590; **^19^F-NMR (376 MHz, CDCl_3_): δ_F_** -61.08**­; ^1^H-NMR (400 MHz, CDCl_3_): δ_H_** 8.02 - 8.22 (m, 3H), 7.59 (d, J = 1.59 Hz, 1H), 7.52 (dd, J = 1.71, 8.44 Hz, 1H), 7.40 - 7.47 (m, 3H), 7.34 - 7.39 (m, 1H), 7.24 - 7.31 (m, 1H), 6.92 (d, J = 8.44 Hz, 1H), 4.01 (s, 2H); **^13^C-NMR (100 MHz CDCl_3_): δ_C_** 146.7, 139.6, 137.3, 128.4 (q, J=3.7 Hz), 126.8, 126.3, 126.2, 123.8, 123.5, 123.3, 120.5, 120.3, 120.0, 119.8, 115.1, 110.9, 26.9; **LR-ESI-MS**: C_19_H_12_F_3_N_2_ [M-H]^-^ *m/z* found 325.01, cald 325.09.

**3-Methyl-2-(4,4,5,5-tetramethyl-1,3,2-dioxaborolan-2-yl)aniline (61b)**

A degassed stirred solution of 2-bromo-3-methylaniline (1.0 g, 5.37 mmol, 1 eq), 4,4,4',4',5,5,5',5'-octamethyl-2,2'-bi(1,3,2-dioxaborolane) (2.73 g, 10.75 mmol, 2 eq), KOAc (1.58 g, 16.1 mmol, 3 eq) and PdCl_2_(dppf) (197 mg, 0.269 mmol, 0.05 eq) in DMF (9 mL, 0.6 M) under an inert N_2_ atmosphere was heated with microwave irradiation at 100°C for 16 h. Following completion the crude mixture was concentrated onto silica gel and purified by Isolera Biotage LPLC (CH/EA 8:2) to give a clear oil which was subsequently purified by SC-X NH_2_ column (MeOH then 7*N* NH_3_ in MeOH) to give **61b** (142 mg, 11 %) as a clear oil.

**v_max_ (cm^-1^)** 2975, 2935, 1459, 1370, 1120, 845; **^1^H-NMR (400 MHz, CDCl_3_): δ_H_** 7.1 (t, J=7.70 Hz, 4 H), 6.5 (d, J=7.46 Hz, 4 H), 6.4 (d, J=8.07 Hz, 1 H), 2.5 (s, 3 H), 1.4 (s, 12 H); **^13^C-NMR (100 MHz CDCl_3_): δ_C_** 154.4, 146.9, 131.6, 119.6, 112.7, 82.9, 24.9, 23.4; **LR-ESI-MS**: C_13_H_21_BNO_2_ [M+H]^+^ *m/z* found 234.36, cald 234.17.

**2-(9*H*-Carbazol-1-yl)-3-methylaniline (61)**

A degassed stirred solution of 1-bromo-9H-carbazole (136 mg, 0.55 mmol, 1 eq), 3-methyl-2-(4,4,5,5-tetramethyl-1,3,2-dioxaborolan-2-yl)aniline **61b** (142 mg, 0.61 mmol, 1.1 eq), Na_2_CO_3_ (176 mg, 1.66 mmol, 3 eq) and Pd(PPh_3_)_4_ (32 mg, 0.028 mmol, 0.05 eq) in a mixture of 1,4-Dioxane:EtOH:H2O (2 mL, 2:1:1, 0.5 M) under an inert N_2_ atmosphere was heated with microwave irradiation at 90°C for 2 h. Following completion the crude mixture was concentrated onto silica gel and purified by Isolera Biotage LPLC (CH/EA 8:2) to give **61** (69 mg, 46 %) as a white solid.

**v_max_ (cm^-1^)** 3394, 3151, 1599, 1415, 801, 654; **^1^H-NMR (400 MHz, CDCl_3_): δ_H_** 8.0 (m, 2 H), 7.9 (br. s., 1 H), 7.3 (m, 1 H), 7.2 (d, J=7.46 Hz, 1 H), 7.2 (m, 1 H), 7.1 (m, 2 H), 7.1 (t, J=7.76 Hz, 1 H), 6.7 (d, J= 7.5 Hz, 1H), 6.6 (d, J= 7.9 Hz, 1H), 3.3 (br. s., 2 H), 1.9 (s, 3 H); **^13^C-NMR (100 MHz CDCl_3_): δ_C_** 144.7, 139.4, 138.3, 138.1, 128.8, 126.9, 125.9, 123.6, 123.5, 122.9, 120.4, 120.2, 119.9, 119.7, 119.4, 112.9, 110.7, 26.9, 20.2; **LR-ESI-MS**: C_19_H_17_N_2_ [M+H]^+^ *m/z* found 273.05, cald 273.14.

***N*-(2-(9H-carbazol-1-yl)phenyl)ethanesulfonamide (62)**

2-(9*H*-Carbazol-1-yl)aniline (100 mg, 0.387 mmol, 1 eq) and ethanesulfonyl chloride (38 μL, 0.4 mmol, 1.1 eq) were reacted according to general procedure A to give **62** (105 mg, 0.362 mmol 74%) as a white solid.

**Mpt:** 157 °C. **v_max_ (cm^-1^)** 3421, 1624, 1602, 1455, 1419, 1320, 1233, 1153, 970, 755, 523; **^1^H-NMR (500 MHz, CD_2_Cl_2_): δ_H_** 8.2 (dd, *J*= 7.4, 1.5 Hz, 1H), 8.1 (d, *J*= 7.8 Hz, 1H), 8.0 (bs, 1H), 7.7 (dd, *J*= 8.3, 1.2 Hz, 1H), 7.5 (td, *J*= 7.9, 1.7, 1H), 7.5-7.4 (m, 3H), 7.3 (t, *J*= 7.3 Hz, 1H), 7.3 (m, 2H), 7.2 (ddd, *J*= 8.0, 6.4, 1.7 Hz, 1H), 6.3 (bs, 1H), 2.9 (qd, *J*= 7.4, 2.2 Hz, 2H), 0.9 (t, *J*= 7.4 Hz, 3H); **^13^C-NMR (101 MHz CD_2_Cl_2_): δ_C_** 140.2, 138.3, 135.6, 131.7, 130.1, 129.9, 126.9, 126.8, 125.9, 124.7, 123.9, 121.3, 121.2, 121.1, 120.7, 120.5, 120.3, 111.5, 47.0, 8.2; **HR-ESI-MS:** C_20_H_17_N_2_O_2_S [M-H]^-^ *m/z* found 349.1016, cald 349.1011.

***N*-(2-(9*H*-Carbazol-1-yl)phenyl)propane-1-sulfonamide (63)**

2-(9*H*-Carbazol-1-yl)aniline (100 mg, 0.387 mmol, 1 eq) and propanesulfonyl chloride (44 μL, 0.39 mmol, 1.1 eq) were reacted according to general procedure A to give **63** (116 mg, 0.318 mmol 82%) as a white solid.

**Mpt:** 160 °C. **v_max_ (cm^-1^)** 3370, 3240, 1600, 1579, 1506, 1489, 1483, 1454, 1410, 1331, 1240, 1156, 928, 754; **^1^H-NMR (500 MHz, CD_2_Cl_2_): δ_H_** 8.18 (ddd, *J*= 7.4, 1.5, 0.6 Hz, 1H), 8.17 (bs, 1H), 8.14 (ddd, *J*= 7.9, 1.0 Hz, 1H), 7.74 (dd, *J*= 8.2, 1.1 Hz, 1H), 7.53-7.49 (m, 1H), 7.47 (ddd, *J*= 7.6, 1.7, 0.5, 1H), 7.43 (dd, *J*= 6.6, 1.2 Hz, 1H), 7.42-7.39 (m, 1H), 7.39-7.34 (m, 2H), 7.34–7.31 (m, 1H), 7.28 (ddd, J = 8.1, 6.6, 1.6 Hz, 1H), 6.41 (bs, 1H), 2.88–2.81 (m, 2H), 1.50–1.36 (m, 2H), 0.76 (t, J =7.4 Hz, 3H); **^13^C-NMR (101 MHz CD_2_Cl_2_): δ_C_** 140.3, 138.4, 135.7, 131.7, 130.0, 129.9, 126.9, 126.8, 125.7, 124.7, 123.9, 121.4, 121.2, 121.0, 120.7, 120.5, 120.4, 111.6, 54.4, 17.6, 12.9; **HR-EI-MS:** C_21_H_20_N_2_O_2_S *m/z* found 364.1218, cald 364.1246.

***N*-(2-(9*H*-Carbazol-1-yl)phenyl)butane-1-sulfonamide (64)**

2-(9*H*-Carbazol-1-yl)aniline (100 mg, 0.387 mmol, 1 eq) and butanesulfonyl chloride (52 μL, 0.4 mmol, 1.1 eq) were reacted according to general procedure A to give **64** (111 mg, 0.293 mmol 76%) as a white solid.

**Mpt:** 75 °C. **v_max_ (cm^-1^)** 3348, 1504, 1482, 1455, 1418, 1398, 1330, 1320, 1235, 1147, 925, 755; **^1^H-NMR (500 MHz, CD_2_Cl_2_): δ_H_** 8.18 (ddd, *J* = 7.6, 1.4, 0.7 Hz, 1H, 4´´-H), 8.13 (dd, *J* = 7.8, 1.0 Hz, 1H, 5´´-H), 8.10 (bs, 1H, 9´´-H), 7.75 (dd, *J* = 8.2, 1.2 Hz, 1H, 3´-H), 7.50 (ddd, *J* = 8.2, 7.4, 1.7 Hz, 1H, 4´-H), 7.47 (dd, *J* = 7.6, 1.6 Hz, 1H, 6´-H), 7.43 (dd, *J* = 6.7, 1.2 Hz, 1H, 2´´-H), 7.42 – 7.40 (m, 1H, 8´´-H), 7.38 (t, *J* = 7.4 Hz, 1H, 3´´-H), 7.36 – 7.32 (m, 2H, 5´-H, 7´´-H), 7.28 (ddd, *J* = 8.0, 6.6, 1.5 Hz, 1H), 6.40 (bs, 1H), 2.88 – 2.83 (m, 2H), 1.41 – 1.25 (m, 2H), 1.23 – 1.02 (m, 2H), 0.67 (t, *J* = 7.3 Hz, 3H); **^13^C-NMR (101 MHz CD_2_Cl_2_): δ_C_** 140.2, 138.3, 135.6, 131.7, 130.0, 129.9, 127.0, 126.8, 125.7, 124.7, 123.9, 121.5, 121.2, 121.0, 120.7, 120.5, 120.4, 111.5, 52.4, 25.7, 21.8, 13.6; **HR-EI-MS:** C_22_H_22_N_2_O_2_S *m/z* found 378.1411, cald 378.1402.

***N*-(2-(9H-carbazol-1-yl)phenyl)-2-(dimethylamino)acetamide (65)**

Initially Bromoacetylbromide (17 μL, 0.2 mmol) was stirred in DCM (5 mL) at 0°C and had 2-(9*H*-Carbazol-1-yl)aniline (34 mg, 0.13 mmol), NEt_3_ (19 μL, 0.14 mmol) added sequentially. The solution was stirred at 0 ° C for 30 minutes and then warmed to room temperature. After adding another 10 mL DCM, 15 mL saturated ammonium chloride solution was used to wash the organic solution. The organic phase was dried over sodium sulfate and the solvent removed under vacuum at room temperature. The residue was dissolved in a mixture of (4 mL) tetrahydrofuran and (8 mL) acetonitrile and then K_2_CO_3_ (138 mg, 1.00 mmol) and Dimethylamine solution (127 μL, 40%, aqueous, 1.13 mmol) was added. The solution was stirred with heating to 95°C for an hour. The resulting solid that formed was filtered off and the filtrate was concentrated under vacuum. The residue was taken up in 20 mL dichloromethane and this solution was washed with H_2_O (x2), sat. NaCl solution (x1) and dried over sodium sulfate before being filtered and concentrated to a residue which was purified using FCC eluting with Ethyl Acetate (100%) to give **65** (39 mg, 0.11 mmol, 87%).

**Mpt:** 198 °C. **v_max_ (cm^-1^)** 3292, 1676, 1580, 1529, 1453, 1412, 1320, 1239, 1043, 766, 752, 735; **^1^H-NMR (400 MHz, CD_2_Cl_2_): δ_H_** 9.36 (bs, 1H), 8.45 (d, *J* = 8.2 Hz, 1H), 8.21 (bs, 1H), 8.15 (dd, *J* = 6.1, 2.8 Hz, 1H), 8.12 (d, *J* = 7.7 Hz, 1H), 7.53–7.44 (m, 2H), 7.43–7.38 (m, 2H), 7.38–7.32 (m, 2H), 7.31–7.19 (m, 2H), 2.85–2.59 (m, 2H), 1.65 (s, 6H, 4-H); **^13^C-NMR (126 MHz CD_2_Cl_2_): δ_C_** 169.6, 140.2, 138.4, 136.5, 130.9, 129.5, 128.9, 127.3, 126.6, 124.7, 124.1, 123.8, 121.3, 120.9, 120.6, 120.2, 120.1, 111.3, 63.7, 45.4; **HR-EI-MS:** C_22_H_21_N_3_O *m/z* found 343.1680, cald 343.1685.

***N*-(2-(9H-carbazol-1-yl)phenyl)propionamide (66)**

2-(9*H*-Carbazol-1-yl)aniline (50 mg, 0.19 mmol, 1 eq) and propionyl chloride (18 μL, 0.21 mmol, 1.1 eq) were reacted according to general procedure A to give **66** (46 mg, 0.15 mmol 76%) as a white solid.

**Mpt:** 166 °C. **v_max_ (cm^-1^)** 3405, 3288, 1682, 1581, 1519, 1496, 1454, 1446, 1319, 1235, 753; **^1^H-NMR (500 MHz, CD_2_Cl_2_): δ_H_** 8.38 (bs, 1H), 8.28 (d, *J* = 8.4 Hz, 1H), 8.16 (ddd, *J* = 6.8, 2.2, 0.7 Hz, 1H), 8.13 (dd, *J* = 7.8, 0.8 Hz, 1H), 7.48–7.43 (m, 2H), 7.43–7.39 (m, 2H), 7.39–7.34 (m, 2H), 7.29 (td, *J* = 7.5, 1.3 Hz, 1H), 7.26 (ddd, *J* = 8.0, 5.7, 2.4 Hz, 1H), 7.11 (bs, 1H), 2.06–1.88 (m, 2H), 0.81 (t, *J* = 7.6 Hz, 3H); **^13^C-NMR (126 MHz CD_2_Cl_2_): δ_C_** 173.0, 140.2, 138.3, 136.2, 131.2, 129.6, 129.3, 127.2, 126.8, 125.3, 124.2, 123.7, 123.0, 121.0, 120.9, 120.8, 120.3, 120.1, 111.4, 31.0, 9.6; **HR-EI-MS:** C_21_H_18_N_2_O *m/z* found 314.1424, cald 314.1419.

***N*-(2-(9H-carbazol-1-yl)phenyl)butyramide (67)**

2-(9*H*-Carbazol-1-yl)aniline (100 mg, 0.387 mmol, 1 eq) and butanoyl chloride (43 μL, 0.416 mmol, 1.1 eq) were reacted according to general procedure A to give **67** (67 mg, 0.2 mmol 51%) as a white solid.

**Mpt:** 145 °C. **v_max_ (cm^-1^)** 3409, 3009, 1681, 1519, 1497, 1455, 1447, 1418, 1320, 1237, 1216, 1052, 1028, 752, 666; **^1^H-NMR (400 MHz, CD_2_Cl_2_): δ_H_** 8.29 (d, *J* = 8.1 Hz, 1H), 8.25 (bs, 1H), 8.16 (dd, *J* = 6.1, 2.8 Hz, 1H), 8.13 (d, *J* = 7.8 Hz, 1H), 7.51–7.43 (m, 2H), 7.43–7.38 (m, 2H), 7.38–7.33 (m, 2H), 7.29 (t, *J* = 9.2, 7.4 Hz, 1H), 7.24 (dd, *J* = 6.4, 1.5 Hz, 1H), 7.07 (bs, 1H), 2.03–1.85 (m, 2H), 1.41–1.30 (m, 2H), 0.69 (t, *J* = 7.4 Hz, 3H); **^13^C-NMR (126 MHz CD_2_Cl_2_): δ_C_** 172.2, 140.2, 138.4, 136.3, 131.2, 129.6, 129.4, 127.2, 126.8, 125.3, 124.3, 123.7, 123.1, 121.1, 120.9, 120.8, 120.4, 120.2, 111.4, 39.9, 19.2, 13.8; **HR-ESI-MS:** C_22_H_21_N_2_O [M+H]^+^ *m/z* found 329.1654, cald 329.1654.

***N*-(2-(9H-carbazol-1-yl)phenyl)pentanamide (68)**

2-(9*H*-Carbazol-1-yl)aniline (100 mg, 0.387 mmol, 1 eq) and butanoyl chloride (49 μL, 0.416 mmol, 1.1 eq) were reacted according to general procedure A to give **68** (109 mg, 0.32 mmol 82%) as a white solid.

**Mpt:** 156 °C. **v_max_ (cm^-1^)** 3395, 3285, 2955, 1687, 1677, 1581, 1518, 1498, 1445, 1420, 1407, 1321, 1299, 1241, 1219, 755; **^1^H-NMR (500 MHz, CD_2_Cl_2_): δ_H_** 8.57 (bs, 1H), 8.30 (d, *J* = 8.2 Hz, 1H), 8.17 (dd, *J* = 5.9, 3.1 Hz, 1H), 8.14 (d, *J* = 7.8 Hz, 1H), 7.48–7.34 (m, 6H), 7.31–7.24 (m, 2H), 7.15 (bs, 1H), 1.99–1.85 (m, 2H), 1.30–1.09 (m, 2H), 1.01 (h, *J* = 7.5 Hz, 2H), 0.60 (t, *J* = 7.3 Hz, 3H); **^13^C-NMR (126 MHz CD_2_Cl_2_): δ_C_** 172.4, 140.3, 138.3, 136.3, 131.2, 129.6, 129.3, 127.2, 126.7, 125.2, 124.2, 123.7, 122.9, 121.1, 120.9, 120.8, 120.3, 120.1, 111.5, 37.7, 27.8, 22.6, 13.8; **HR-EI-MS:** C_23_H_22_N_2_O *m/z* found 342.1734, cald 342.1732.

***N*-(2-(9*H*-Carbazol-1-yl)phenyl)-2-methoxyethane-1-sulfonamide (69)**

2-(9*H*-Carbazol-1-yl)aniline (50 mg, 0.194 mmol, 1 eq) and 2-methoxyethane-1-sulfonyl chloride (18.71 μL, 0.232 mmol, 1.2 eq) were reacted according to general procedure **A** to give **69** (33 mg, 0.087 mmol 45%) as a white solid.

**v_max_ (cm^-1^)** 3366, 3266, 3219, 2919, 1600, 1322, 1107, 922, 740; **^1^H-NMR (400 MHz, CDCl_3_): δ_H_** 8.5 (s, 1 H), 8.1 (m, 2 H), 7.8 (dd, J=8.31, 0.86 Hz, 1 H), 7.3 (m, 6 H), 7.2 (m, 2 H), 6.5 (s, 1 H), 3.5 (m, 2 H), 3.2 (td, J=5.53, 2.38 Hz, 2 H), 2.8 (s, 3 H); **^13^C-NMR (100 MHz CDCl_3_): δ_C_** 139.6, 137.6, 135.3, 131.4, 129.3, 128.5, 126.9, 126.3, 124.6, 123.9, 123.3, 120.7, 120.6, 120.0, 119.8, 119.0, 110.7, 66.2, 58.5, 52.4, 26.9; **LR-ESI-MS**: C_21_H_21_N_2_O_3_S [M+H]^+^ *m/z* found 381.16, cald 381.13.

***N*-(2-(9*H*-Carbazol-1-yl)phenyl)-5-methylisoxazole-4-sulfonamide (70)**

2-(9*H*-Carbazol-1-yl)aniline (50 mg, 0.194 mmol, 1 eq) and 5-methylisoxazole-4-sulfonyl chloride (39 mg, 0.213 mmol, 1.1 eq) were reacted according to general procedure A to give **70** (6 mg, 0.015 mmol 8%) as a white solid.

**^1^H-NMR (400 MHz, CDCl_3_): δ_H_** 8.1 (t, J=7.09 Hz, 2 H), 7.9 (d, J=8.07 Hz, 1 H), 7.8 (s, 1 H), 7.5 (m, 3 H), 7.4 (m, 3 H), 7.3 (t, J=7.64 Hz, 2 H), 7.0 (dd, J=7.34, 0.98 Hz, 1 H), 6.8 (br. s., 1 H), 1.9 (s, 3 H); **^13^C-NMR (100 MHz CDCl_3_): δ_C_** 172.0, 148.5, 139.7, 137.3, 133.4, 131.3, 130.9, 129.6, 127.0, 126.8, 125.8, 124.3, 123.8, 123.4, 120.8, 120.6, 120.5, 119.3, 116.1, 111.4, 11.0; **LR-ESI-MS**: C_22_H_18_N_3_O_3_S [M+H]^+^ *m/z* found 404.14, cald 404.11.

***N_1_*-(2-(9*H*-Carbazol-1-yl)phenyl)-*N_2_*,*N_2_*-dimethylsulfamide (71)**

2-(9*H*-Carbazol-1-yl)aniline (50 mg, 0.194 mmol, 1 eq) and dimethylsulfamoyl chloride (31 mg, 0.213 mmol, 1.1 eq) were reacted according to general procedure A to give **71** (8 mg, 0.022 mmol 12%) as a white solid.

**^1^H-NMR (400 MHz, CDCl_3_): δ_H_** 8.0 (m, 2 H), 8.0 (s, 1 H), 7.6 (dd, J=8.25, 0.92 Hz, 1 H), 7.3 (m, 4 H), 7.3 (m, 2 H), 7.2 (m, 2 H), 6.3 (s, 1 H), 2.6 (s, 6 H); **^13^C-NMR (100 MHz CDCl_3_): δ_C_** 139.6, 137.6, 135.5, 130.9, 129.3, 128.3, 126.5, 126.4, 124.5, 124.2, 123.3, 120.8, 120.6, 120.2, 119.9, 119.9, 119.6, 111.0, 37.9 **LR-ESI-MS:** C_20_H_20_N_3_O_2_S [M+H]^+^ *m/z* found 366.15, cald 366.13.

***N*-(2-(9*H*-Carbazol-1-yl)phenyl)benzenesulfonamide (72)**

2-(9*H*-Carbazol-1-yl)aniline (50 mg, 0.194 mmol, 1 eq) and benzenesulfonyl chloride (27 μL, 0.213 mmol, 1.1 eq) were reacted according to general procedure A to give **72** (66 mg, 0.165 mmol 85%) as a white solid.

**^1^H-NMR (400 MHz, CDCl_3_): δ_H_** 8.1 (dd, J=7.64, 3.61 Hz, 2 H), 7.8 (d, J=8.07 Hz, 1 H), 7.4 (m, 5 H), 7.3 (m, 6 H), 7.1 (m, 2 H), 6.9 (dd, J=7.27, 0.92 Hz, 1 H), 6.7 (s, 1 H); **^13^C-NMR (100 MHz CDCl_3_): δ_C_** 139.5, 138.6, 137.6, 134.4, 132.8, 130.5, 129.9, 129.3, 128.7, 126.9, 126.4, 125.8, 125.6, 123.9, 123.2, 122.4, 120.6, 120.5, 119.9, 119.2, 110.9; **LR-ESI-MS:** C_24_H_19_N_2_O_2_S [M+H]^+^ *m/z* found 399.12, cald 399.12.

***N*-(2-(9H-carbazol-1-yl)phenyl)-2,2,2-trifluoroacetamide (73)**

2-(9*H*-Carbazol-1-yl)aniline (100 mg, 0.387 mmol, 1 eq) and Trifluoroacetic anhydride (56 μL, 0.4 mmol, 1.1 eq) were reacted according to general procedure A to give **73** (90 mg, 0.25 mmol 66%) as a white solid.

**Mpt:** 162 °C. **v_max_ (cm^-1^)** 3381, 1720, 1585, 1544, 1457, 1418, 1320, 1274, 1234, 1201, 1160, 754, 736; **^1^H-NMR (400 MHz, CD_2_Cl_2_): δ_H_** 8.35 (d, *J* = 7.8 Hz, 1H), 8.20 (ddd, *J* = 7.0, 2.0, 0.7 Hz, 1H), 8.14 (d, *J* = 7.8 Hz, 1H), 8.06 (bs, 1H), 8.02 (bs, 1H), 7.60–7.55 (m, 2H), 7.48–7.35 (m, 5H), 7.28 (ddd, *J* = 8.0, 6.6, 1.5 Hz, 1H); **^13^C-NMR (101 MHz CD_2_Cl_2_): δ_C_** 155.2 (q, *J* = 37.3 Hz), 140.2, 138.3, 133.5, 131.2, 130.1, 129.8, 127.3, 127.0, 126.7, 124.8, 123.7, 122.5, 121.5, 121.0, 120.6, 120.5, 119.4, 114.6, 111.4; **HR-EI-MS:** C_20_H_13­­_F_3_N_2_O *m/z* found 354.0965, cald 354.0980.

***N*-(2-(9H-carbazol-1-yl)phenyl)cyclopropanecarboxamide (74)**

2-(9*H*-Carbazol-1-yl)aniline (100 mg, 0.387 mmol, 1 eq) and cyclopropylcarbonyl chloride (38 μL, 0.4 mmol, 1.1 eq) were reacted according to general procedure A to give **74** (118 mg, 0.362 mmol 93%) as a white solid.

**Mpt:** 155 °C. **v_max_ (cm^-1^)** 3398, 3389, 3289, 1676, 1582, 1522, 1497, 1443, 1419, 1319, 1300, 1237, 956, 755; **^1^H-NMR (500 MHz, CD_2_Cl_2_): δ_H_** 8.33 (bs, 1H), 8.24 (d, *J* = 8.3 Hz, 1H), 8.16 (dt, *J* = 7.5, 0.9 Hz, 1H), 8.13 (dd, *J* = 7.9, 0.7 Hz, 1H), 7.47–7.43 (m, 2H), 7.43–7.39 (m, 3H), 7.37 (t, *J* = 7.4 Hz, 1H), 7.33 (bs, 1H), 7.29 (td, *J* = 7.4, 1.2 Hz, 1H), 7.26 (ddd, *J* = 8.0, 6.6, 1.5 Hz, 1H), 1.13–0.95 (m, 1H), 0.96–0.43 (m, 4H); **^13^C-NMR (126 MHz CD_2_Cl_2_): δ_C_** 173.1, 140.2, 138.4, 136.3, 131.2, 129.6, 129.3, 127.2, 126.7, 125.3, 124.2, 123.7, 123.3, 121.2, 120.9, 120.8, 120.3, 120.1, 111.4, 15.9, 8.2; **HR-EI-MS:** C_22_H_18_N_2_O *m/z* found 326.1427, cald 326.1419.

***N*-(2-(9H-carbazol-1-yl)phenyl)cyclohexanecarboxamide (75)**

2-(9*H*-Carbazol-1-yl)aniline (100 mg, 0.387 mmol, 1 eq) and cyclohexylcarbonyl chloride (54 μL, 0.4 mmol, 1.1 eq) were reacted according to general procedure A to give **75** (113 mg, 0.307 mmol 79%) as a beige solid.

**Mpt:** 150 °C. **v_max_ (cm^-1^)** 3401, 3278, 2928, 1629, 1517, 1446, 1414, 1319, 1232, 1232, 747, 735; **^1^H-NMR (500 MHz, CD_2_Cl_2_): δ_H_** 8.50 (bs, 1H), 8.26 (d, *J* = 8.2 Hz, 1H), 8.17 (dd, *J* = 6.1, 2.8 Hz, 1H), 8.14 (d, *J* = 7.9 Hz, 1H), 7.51–7.32 (m, 6H), 7.34–7.21 (m, 2H), 7.20 (bs, 1H), 1.81 (tt, *J* = 11.5, 3.5 Hz, 1H), 1.65–1.29 (m, 5H), 1.18–0.88 (m, 5H); **^13^C-NMR (126 MHz CD_2_Cl_2_): δ_C_** 175.3, 140.2, 138.3, 136.3, 131.1, 129.9, 129.3, 127.2, 126.7, 125.3, 124.2, 123.6, 123.3, 121.1, 120.8, 120.8, 120.2, 120.1, 111.5, 46.3, 29.7, 29.6, 26.0, 25.9, 25.8; **HR-EI-MS:** C_25_H_24_N_2_O *m/z* found 368.1866, cald 368.1889.

***N*-(2-(9H-carbazol-1-yl)phenyl)furan-2-carboxamide (76)**

2-(9*H*-Carbazol-1-yl)aniline (50 mg, 0.19 mmol, 1 eq) and furan-2-carbonyl chloride (54 μL, 0.4 mmol, 1.1 eq) were reacted according to general procedure A to give **76** (45 mg, 0.13 mmol 64%) as a white solid.

**Mpt:** 173 °C. **v_max_ (cm^-1^)** 3386, 1672, 1583, 1528, 1449, 1318, 1109, 751; **^1^H-NMR (400 MHz, CD_2_Cl_2_): δ_H_** 8.53 (d, *J* = 8.7 Hz, 1H), 8.25 (bs, 1H), 8.21–8.16 (m, 2H), 8.14 (d, *J* = 7.8 Hz, 1H), 7.54–7.47 (m, 2H), 7.45–7.35 (m, 4H), 7.32 (td, *J* = 7.5, 1.2 Hz, 1H), 7.25 (ddd, *J* = 8.1, 6.5, 1.7 Hz, 1H), 7.12 (dd, *J* = 1.7, 0.8 Hz, 1H), 6.97 (dd, *J* = 3.5, 0.8 Hz, 1H), 6.36 (dd, *J* = 3.5, 1.8 Hz, 1H); **^13^C-NMR (126 MHz CD_2_Cl_2_): δ_C_** 156.6, 148.2, 145.0, 140.2, 138.5, 135.9, 131.0, 129.5, 129.0, 127.1, 126.8, 125.2, 124.5, 123.7, 122.0, 121.0, 120.9, 120.6, 120.4, 120.2, 115.2, 112.7, 111.4; **HR-ESI-MS:** C_23_H_17_N_2_O_2_ *m/z* [M+H]^+^ found 353.1291, cald 353.1290.

***N*-(2-(9H-carbazol-1-yl)phenyl)benzamide (77)**

2-(9*H*-Carbazol-1-yl)aniline (100 mg, 0.387 mmol, 1 eq) and benzoyl chloride (48 μL, 0.42 mmol, 1.1 eq) were reacted according to general procedure A to give **77** (82 mg, 0.23 mmol 57%) as a white solid.

**Mpt:** 210 °C. **v_max_ (cm^-1^)** 3405, 3277, 1662, 1583, 1528, 1498, 1450,1418, 1314, 1241, 759, 753, 682, 583; **^1^H-NMR (400 MHz, CD_2_Cl_2_): δ_H_** 8.55 (d, *J* = 8.0 Hz, 1H), 8.31 (bs, 1H), 8.19 (d, *J* = 7.7 Hz, 1H), 8.13 (d, *J* = 7.8 Hz, 1H), 7.95 (bs, 1H), 7.57–7.49 (m, 2H), 7.45 (d, *J* = 7.4 Hz, 1H), 7.43–7.35 (m, 4H), 7.35–7.29 (m, 3H), 7.25 (ddd, *J* = 7.9, 6.0, 2.0 Hz, 1H), 7.19 (t, *J* = 7.7 Hz, 2H); **^13^C-NMR (126 MHz CD_2_Cl_2_): δ_C_** 166.0, 140.2, 138.4, 136.4, 135.2, 132.2, 131.0, 129.6, 129.5, 129.1, 127.2, 126.9, 125.4, 124.5, 123.7, 122.4, 121.1, 121.0, 120.9, 120.5, 120.3, 111.4; **HR-ESI-MS:** C_25_H_17_N_2_O *m/z* [M-H]^-^ found 361.1347, cald 361.1341.

***N*-(2-(9*H*-Carbazol-1-yl)phenyl)-2-phenylacetamide (78)**

2-(9*H*-Carbazol-1-yl)aniline (50 mg, 0.194 mmol, 1 eq) and 2-phenylacetyl chloride (33 mg, 0.213 mmol, 1.1 eq) were reacted according to general procedure A to give **78** (41 mg, 0.11 mmol 57%) as a white solid.

**v_max_ (cm^-1^)** 3354, 3311, 1661, 1520, 1447, 1320, 1238, 748; **^1^H-NMR (400 MHz, CDCl_3_): δ_H_** 8.3 (d, J=8.19 Hz, 1 H), 8.0 (d, J=7.70 Hz, 1 H), 8.0 (d, J=7.70 Hz, 1 H), 7.9 (s, 1 H), 7.4 (m, 2 H), 7.2 (m, 3 H), 7.1 (m, 2 H), 7.0 (dd, J=7.21, 0.98 Hz, 1 H), 6.9 (br. s., 1 H), 6.8 (m, 1 H), 6.6 (t, J=7.64 Hz, 2 H), 6.5 (d, J=7.34 Hz, 2 H), 3.3 (m, 2 H); **^13^C-NMR (100 MHz CDCl_3_): δ_C_** 169.6, 139.5, 137.2, 135.4, 132.9, 130.6, 128.9, 128.7, 128.6, 128.5, 127.1, 126.4, 126.2, 124.8, 123.6, 123.5, 121.3, 120.4, 120.2, 119.8, 119.7, 110.9, 44.8; **LR-ESI-MS**: C_26_H_21_N_2_O [M+H]^+^ *m/z* found 377.21, cald 377.17.

***N*-(2-(9*H*-Carbazol-1-yl)phenyl)-1-phenylmethanesulfonamide (79)**

2-(9*H*-Carbazol-1-yl)aniline (50 mg, 0.194 mmol, 1 eq) and phenylmethanesulfonyl chloride (41 mg, 0.213 mmol, 1.1 eq) were reacted according to general procedure A to give **79** (13 mg, 0.032 mmol 16%) as a white solid.

**^1^H-NMR (400MHz, CDCl_3_): δ_H_** 8.1 (d, J=7.82 Hz, 2 H), 7.9 (s, 1 H), 7.8 (dd, J=8.25, 0.67 Hz, 1 H), 7.5 (m, 4 H), 7.3 (m, 5 H), 7.2 (m, 1 H), 7.1 (m, 3 H), 6.3 (s, 1 H), 4.3 (m, 2 H); **^13^C-NMR (100 MHz CDCl_3_): δ_C_** 139.5, 137.6, 135.2, 131.2, 130.5, 129.6, 128.9, 128.8, 128.1, 127.9, 126.5, 126.1, 124.6, 124.2, 123.3, 120.8, 120.6, 120.0, 119.9, 119.1, 118.7, 110.9, 57.9; **LR-ESI-MS:** C_25_H_21_N_2_O_2_S [M+H]^+^ *m/z* found 413.16, cald 413.13.

***N*-(2-(9*H*-Carbazol-1-yl)phenyl)-2-methoxybenzamide (80)**

2-(9*H*-Carbazol-1-yl)aniline (50 mg, 0.194 mmol, 1 eq) and 2-methoxybenzoyl chloride (32 μL, 0.213 mmol, 1.1 eq) were reacted according to general procedure A to give **80** (55 mg, 0.140 mmol 73%) as a white solid.

**^1^H-NMR (400 MHz, CDCl_3_): δ_H_** 9.8 (s, 1 H), 8.7 (m, 1 H), 8.1 (m, 4 H), 7.4 (m, 2 H), 7.3 (m, 4 H), 7.2 (m, 3 H), 6.9 (m, 1 H), 6.5 (d, J=8.07 Hz, 1 H), 2.8 (s, 3 H); **^13^C-NMR (100 MHz CDCl_3_): δ_C_** 163.7, 157.0, 139.6, 138.1, 136.9, 133.1, 132.4, 130.8, 129.1, 128.4, 127.1, 126.2, 124.2, 123.6, 123.1, 121.9, 121.4, 121.1, 121.0, 120.2, 120.0, 119.8, 119.5, 110.9, 54.9; **LR-ESI-MS**: C_26_H_21_N_2_O_2_ [M+H]^+^ *m/z* found 393.14, cald 393.16.

***N*-(2-(9*H*-Carbazol-1-yl)phenyl)-3-methoxybenzamide (81)**

2-(9*H*-Carbazol-1-yl)aniline (50 mg, 0.194 mmol, 1 eq) and 3-methoxybenzoyl chloride (30 μL, 0.213 mmol, 1.1 eq) were reacted according to general procedure A to give **81** (72 mg, 0.184 mmol 95%) as a white solid.

**v_max_ (cm^-1^)** 3391, 3281, 1655, 1485, 1026, 734, 591, 542; **^1^H-NMR (400 MHz, CDCl_3_): δ_H_** 8.5 (d, J=8.07 Hz, 1 H), 8.3 (s, 1 H), 8.1 (m, 2 H), 7.8 (s, 1 H), 7.3 (m, 6 H), 7.2 (m, 2 H), 6.9 (m, 1 H), 6.7 (m, 2 H), 6.7 (m, 1 H), 3.3 (s, 3 H); **^13^C-NMR (100 MHz CDCl_3_): δ_C_** 165.5, 159.6, 139.7, 137.6, 135.9, 135.7, 130.4, 129.6, 129.2, 128.4, 126.7, 126.5, 124.8, 123.9, 123.2, 121.4, 120.6, 120.4, 120.2, 120.1, 119.9, 118.9, 118.8, 111.0, 110.4, 54.9; **LR-ESI-MS**: C_26_H_21_N_2_O_2_ [M+H]^+^ *m/z* found 393.16, cald 393.16.

***N*-(2-(9*H*-Carbazol-1-yl)phenyl)-4-methoxybenzamide (82)**

2-(9H-carbazol-1-yl)aniline (50 mg, 0.194 mmol, 1 eq) and 4-methoxybenzoyl chloride (36 mg, 0.213 mmol, 1.1 eq) were reacted according to general procedure A to give **82** (56 mg, 0.142 mmol 74%) as a white solid.

**v_max_ (cm^-1^)** 3400, 3258, 1652, 1600, 1504, 1313, 1170, 801, 587; **^1^H-NMR (400 MHz, CDCl_3_): δ_H_** 8.5 (m, 1 H), 8.2 (s, 1 H), 8.1 (m, 2 H), 7.8 (s, 1 H), 7.4 (m, 2 H), 7.3 (m, 4 H), 7.2 (m, 4 H), 6.5 (m, 2 H), 3.6 (s, 3 H); **^13^C-NMR (100 MHz CDCl_3_): δ_C_** 165.1, 162.3, 139.6, 137.7, 135.9, 130.3, 129.2, 128.5, 128.4, 126.6, 126.6, 126.4, 124.5, 123.9, 123.2, 121.6, 120.6, 120.5, 120.2, 119.9, 119.8, 113.8, 110.9, 55.3; **LR-ESI-MS**: C_26_H_21_N_2_O_2_ [M+H]^+^ *m/z* found 393.13, cald 393.16.

***N*-(2-(9*H*-Carbazol-1-yl)phenyl)-2-(pyridin-3-yl)acetamide (83)**

2-(9*H*-Carbazol-1-yl)aniline (50 mg, 0.194 mmol, 1 eq), 2-(pyridin-3-yl)acetic acid (32 mg, 0.232 mmol, 1.2 eq) and DIPEA (162 μL, 0.929 mmol, 4.8 eq) were dissolved in DCM (2 mL, 0.1 M) and then had HATU (88 mg, 0.232 mmol, 1.2 eq) added before the solution was stirred for 16 h at room temperature. Upon reaction completion the crude mixture was concentrated onto silica gel before being purified by Isolera Biotage LPLC (CH/EA 8:2) to give **83** (22 mg, 0.057 mmol, 29%) as a white solid.

**Mpt:** 145.2-147.2 °C; **v_max_ (cm^-1^)** 1675, 1498, 1240, 1015, 839, 751, 702; **^1^H-NMR (400 MHz, DMSO-*d*^6^): δ_H_** 10.7 (s, 1 H), 9.2 (s, 1 H), 8.3 (m, 1 H), 8.3 (s, 1 H), 8.2 (m, 2 H), 7.8 (m, 1 H), 7.5 (d, J=8.07 Hz, 3 H), 7.4 (s, 2 H), 7.3 (m, 1 H), 7.2 (m, 3 H), 7.1 (m, 1 H), 3.4 (s, 2 H); **^13^C-NMR (100 MHz DMSO-*d*^6^): δ_C_** 168.8, 164.6, 149.9, 147.5, 140.2, 137.7, 136.3, 135.6, 132.5, 131.3, 130.7, 127.9, 126.3, 125.7, 125.5, 123.2, 123.2, 122.6, 121.4, 120.0, 119.5, 118.6, 118.6, 111.5, 38.2; **LR-ESI-MS**: C_25_H_18_N_3_O [M-H]^-^ *m/z* found 378.25, cald 378.16.

***N*-(2-(9*H*-Carbazol-1-yl)phenyl)-2-(6-methylpyridin-3-yl)acetamide (84)**

2-(9H-carbazol-1-yl)aniline (50 mg, 0.194 mmol, 1 eq), 2-(6-methylpyridin-3-yl)acetic acid (35 mg, 0.232 mmol, 1.2 eq) and DIPEA (162 μL, 0.929 mmol, 4.8 eq) were dissolved in DCM (2 mL, 0.1 M) and then had HATU (88 mg, 0.232 mmol, 1.2 eq) added before the solution was stirred for 16 h at room temperature. Upon reaction completion the crude mixture was concentrated onto silica gel before being purified by Isolera Biotage LPLC (CH/EA 8:2) to give **84** (56 mg, 0.143 mmol, 74%) as a white solid.

**Mpt:** 126.6-128.6 °C; **v_max_ (cm^-1^)** 2981, 1683, 1602, 1287, 1021, 839, 732; **^1^H-NMR (400 MHz, DMSO-*d*^6^): δ_H_** 10.7 (s, 1 H), 9.0 (s, 1 H), 8.1 (m, 3 H), 7.8 (d, J=7.95 Hz, 1 H), 7.4 (d, J=7.09 Hz, 3 H), 7.3 (m, 2 H), 7.1 (m, 4 H), 6.8 (d, J=7.95 Hz, 1 H), 2.3 (m, 3 H); **^13^C-NMR (100 MHz DMSO-*d*^6^): δ_C_** 168.9, 155.8, 149.1, 140.2, 137.6, 136.5, 135.6, 132.3, 130.7, 128.0, 126.3, 125.6, 125.3, 123.2, 122.6, 122.5, 121.3, 120.1, 119.6, 118.7, 118.6, 111.5, 23.6; **LR-ESI-MS**: C_26_H_22_N_3_O [M+H]^+^ *m/z* found 392.26, cald 392.18.

***N*-(2-(9*H*-Carbazol-1-yl)phenyl)-2-(pyrazin-2-yl)acetamide (85)**

2-(9*H*-Carbazol-1-yl)aniline (50 mg, 0.194 mmol, 1 eq), 2-(pyrazin-2-yl)acetic acid (40 mg, 0.29 mmol, 1.5 eq) and DIPEA (162 μL, 0.929 mmol, 4.8 eq) were dissolved in DCM (2 mL, 0.1 M) and then had HATU (88 mg, 0.232 mmol, 1.2 eq) added before the solution was stirred for 16 h at room temperature. Upon reaction completion the crude mixture was concentrated onto silica gel before being purified by Isolera Biotage LPLC (CH/EA 8:2) to give **85** (32 mg, 0.084 mmol, 44%) as a white solid.

**Mpt:** 189.0-191.0 °C; **v_max_ (cm^-1^)** 3296, 1659, 1581, 1532, 1312, 1236, 738; **^1^H-NMR (400 MHz, CDCl_3_): δ_H_** 8.8 (br. s., 1 H), 8.3 (d, J=7.95 Hz, 1 H), 8.0 (m, 3 H), 7.8 (d, J=2.57 Hz, 2 H), 7.4 (m, 1 H), 7.3 (m, 5 H), 7.2 (m, 3 H), 3.5 (m, 2 H); **^13^C-NMR (100 MHz CDCl_3_): δ_C_** 166.6, 149.4, 144.1, 142.8, 142.6, 139.2, 137.6, 135.5, 130.9, 129.2, 128.9, 126.9, 126.3, 125.0, 123.4, 123.1, 122.4, 120.8, 120.3, 120.0, 119.8, 119.7, 110.7, 42.9; **LR-ESI-MS**: C_24_H_19_N_4_O [M+H]^+^ *m/z* found 379.25, cald 379.16.

***N*-(2-(9*H*-Carbazol-1-yl)phenyl)-2-(2-cyanopyridin-4-yl)acetamide (86)**

2-(9*H*-Carbazol-1-yl)aniline (50 mg, 0.194 mmol, 1 eq), 2-(2-cyanopyridin-4-yl)acetic acid (38 mg, 0.232 mmol, 1.2 eq) and DIPEA (101 μL, 0.581 mmol, 3 eq) were dissolved in DCM (2 mL, 0.1 M) and then had HATU (88 mg, 0.232 mmol, 1.2 eq) added before the solution was stirred for 16 h at room temperature. Upon reaction completion the crude mixture was concentrated onto silica gel before being purified by Isolera Biotage LPLC (CH/EA 8:2) to give **86** (57 mg, 0.142 mmol, 73%) as a white solid.

**Mpt:** 194.1-196.1 °C; **v_max_ (cm^-1^)** 3387, 3319, 1671, 1523, 1313, 1218, 750; **^1^H-NMR (400 MHz, DMSO-*d*^6^): δ_H_** 10.7 (s, 1 H), 9.4 (s, 1 H), 8.4 (d, J=5.01 Hz, 1 H), 8.1 (dd, J=8.19, 6.60 Hz, 2 H), 7.8 (d, J=7.82 Hz, 1 H), 7.6 (s, 1 H), 7.4 (m, 5 H), 7.2 (d, J=4.03 Hz, 1 H), 7.2 (m, 3 H), 3.5 (s, 2 H); **^13^C-NMR (100 MHz DMSO-*d*^6^): δ_C_** 167.7, 150.8, 146.7, 140.1, 137.6, 135.4, 133.1, 132.3, 130.8, 129.4, 128.1, 128.0, 126.2, 125.9, 125.5, 123.1, 122.4, 121.6, 120.1, 119.5, 118.6, 118.4, 117.4, 111.3, 41.2; **LR-ESI-MS**: C_26_H_19_N_4_O [M+H]^+^ *m/z* found 403.53, cald 403.16.

***N*-(2-(9*H*-Carbazol-1-yl)phenyl)-2-(6-cyanopyridin-3-yl)acetamide (87)**

2-(9*H*-Carbazol-1-yl)aniline (50 mg, 0.194 mmol, 1 eq), 2-(6-cyanopyridin-3-yl)acetic acid (38 mg, 0.232 mmol, 1.2 eq) and DIPEA (101 μL, 0.581 mmol, 3 eq) were dissolved in DCM (2 mL, 0.1 M) and then had HATU (88 mg, 0.232 mmol, 1.2 eq) added before the solution was stirred for 16 h at room temperature. Upon reaction completion the crude mixture was concentrated onto silica gel before being purified by Isolera Biotage LPLC (CH/EA 8:2) to give **87** (64 mg, 0.159 mmol, 82%) as a white solid.

**Mpt:** 220.3-222.3 °C; **v_max_ (cm^-1^)** 3280, 2980, 1667, 1581, 1519, 1445, 1318, 1032, 751; **^1^H-NMR (400 MHz, DMSO-*d*^6^): δ_H_** 10.6 (s, 1 H), 9.3 (s, 1 H), 8.4 (s, 1 H), 8.1 (m, 2 H), 7.8 (d, J=7.82 Hz, 1 H), 7.6 (d, J=7.95 Hz, 1 H), 7.5 (m, 3 H), 7.4 (m, 3 H), 7.2 (m, 3 H), 3.6 (s, 2 H); **^13^C-NMR (100 MHz DMSO-*d*^6^): δ_C_** 167.8, 151.5, 140.1, 137.6, 137.5, 136.0, 135.4, 132.8, 130.8, 130.5, 128.3, 128.0, 126.3, 125.8, 125.5, 123.0, 122.5, 121.5, 120.0, 119.5, 118.6, 118.5, 117.5, 111.4; **LR-ESI-MS**: C_26_H_19_N_4_O [M+H]^+^ *m/z* found 403.56, cald 403.16.

***N*-(2-(9*H*-Carbazol-1-yl)phenyl)-2-(6-oxo-1,6-dihydropyridin-3-yl)acetamide (88)**

2-(9*H*-Carbazol-1-yl)aniline (50 mg, 0.194 mmol, 1 eq), 2-(6-oxo-1,6-dihydropyridin-3-yl)acetic acid (36 mg, 0.232 mmol, 1.2 eq) and DIPEA (101 μL, 0.581 mmol, 3 eq) were dissolved in DCM (2 mL, 0.1 M) and then had HATU (88 mg, 0.232 mmol, 1.2 eq) added before the solution was stirred for 16 h at room temperature. Upon reaction completion the crude mixture was concentrated onto silica gel before being purified by Isolera Biotage LPLC (CH/EA 8:2) to give **88** (49 mg, 0.123 mmol, 64%) as a white solid.

**Mpt:** 219.4-221.4 °C; **v_max_ (cm^-1^)** 3348, 3260, 1662, 1624, 1518, 1445, 1238, 555; **^1^H-NMR (400 MHz, DMSO-*d*^6^): δ_H_** 11.3 (br. s., 1 H), 10.8 (s, 1 H), 9.0 (s, 1 H), 8.1 (m, 2 H), 7.8 (d, J=7.95 Hz, 1 H), 7.5 (m, 3 H), 7.4 (m, 2 H), 7.2 (m, 3 H), 7.0 (d, J=1.71 Hz, 1 H), 6.9 (dd, J=9.35, 2.38 Hz, 1 H), 6.0 (d, J=9.29 Hz, 1 H), 3.2 (m, 2 H); **^13^C-NMR (100 MHz DMSO-*d*^6^): δ_C_** 169.2, 161.6, 142.2, 140.1, 137.6, 135.6, 134.1, 132.2, 130.6, 127.9, 126.2, 125.5, 125.5, 125.3, 123.2, 122.5, 121.3, 120.1, 119.6, 119.5, 118.6, 118.5, 112.1, 111.4, 37.9; **LR-ESI-MS**: C_25_H_20_N_3_O_2_ [M+H]^+^ *m/z* found 394.56, cald 394.16.

***N*-(2-(9*H*-Carbazol-1-yl)phenyl)-1-(2-fluorophenyl)methanesulfonamide (89)**

2-(9*H*-Carbazol-1-yl)aniline (50 mg, 0.194 mmol, 1 eq) and (2-fluorophenyl)methanesulfonyl chloride (44 mg, 0.213 mmol, 1.1 eq) were reacted according to general procedure A to give **89** (34 mg, 0.079 mmol 41%) as a white solid.

**Mpt:** 176.9-178.9 °C; **v_max_ (cm^-1^)** 3340, 1585, 1491, 1184, 1113, 748, 503; **^19^F-{H}-NMR (376 MHz, DMSO-*d*^6^): δ_F_** -116.6; **^1^H-NMR (400MHz, DMSO-*d*^6^): δ_H_** 10.8 (s, 1 H), 8.8 (s, 1 H), 8.1 (m, 2 H), 7.5 (m, 4 H), 7.4 (m, 4 H), 7.3 (m, 1 H), 7.1 (m, 4 H), 4.1 (s, 2 H); **^13^C-NMR (100 MHz DMSO-*d*^6^): δ_C_** 160.8 (d, J=247.96 Hz), 140.2, 138.0, 135.1, 133.7, 132.9 (d, J=2.93 Hz), 131.3, 130.5 (d, J=8.80 Hz), 128.5, 127.0, 126.0, 125.4 (d, J=22.01 Hz), 124.2 (d, J=5.13 Hz), 123.0, 122.5, 121.6, 120.0, 119.6, 118.6, 118.5, 116.7 (d, J=13.94 Hz), 115.4 (d, J=21.27 Hz), 111.4, 51.8; **LR-ESI-MS**: C_25_H_20_FN_2_O_2_S [M+H]^+^ *m/z* found 431.78, cald 431.12.

***N*-(2-(9*H*-Carbazol-1-yl)phenyl)-2-(2-fluorophenyl)acetamide (90)**

2-(9*H*-Carbazol-1-yl)aniline (50 mg, 0.194 mmol, 1 eq) and 2-(2-fluorophenyl)acetyl chloride (33 μL, 0.213 mmol, 1.1 eq) were reacted according to general procedure A to give **90** (42 mg, 0.106 mmol, 55%) as a white solid.

**v_max_ (cm^-1^)** 3372, 3226, 1676, 1582, 1467, 1335, 1231, 747; **^19^F-{H}-NMR (376 MHz, DMSO-*d*^6^): δ_F_** -117.5; **^1^H-NMR (400 MHz, DMSO-*d*^6^): δ_H_** 10.7 (s, 1 H), 8.9 (s, 1 H), 8.1 (m, 2 H), 7.9 (d, J=7.95 Hz, 1 H), 7.5 (m, 3 H), 7.4 (m, 2 H), 7.2 (m, 4 H), 6.9 (m, 3 H), 3.5 (s, 2 H); **^13^C-NMR (100 MHz DMSO-*d*^6^): δ_C_** 168.2, 161.5, 159.0, 140.2, 137.6, 135.7, 131.9, 131.3 (d, J=4.40 Hz), 130.7, 128.6 (d, J=8.07 Hz), 128.0, 126.3, 125.5, 125.3, 124.8, 124.0 (d, J=3.67 Hz), 123.2, 122.6, 122.3 (d, J=16.14 Hz), 121.2, 119.8 (d, J= 44.8 Hz), 118.6, 114.9, 114.7, 111.5, 35.8; **LR-ESI-MS**: C_26_H_18_FN_2_O [M-H]^-^ *m/z* found 393.08, cald 393.14.

***N*-(2-(9*H*-Carbazol-1-yl)phenyl)-2-(2-chlorophenyl)acetamide (91)**

2-(9*H*-Carbazol-1-yl)aniline (50 mg, 0.194 mmol, 1 eq) and 2-(2-chlorophenyl)acetyl chloride (40 mg, 0.213 mmol, 1.1 eq) were reacted according to general procedure A to give **91** (39 mg, 0.095 mmol 49%) as a white solid.

**Mpt:** 158.2-160.2 °C; **v_max_ (cm^-1^)** 3351, 3241, 1671, 1522, 1445, 1235, 1052; **^1^H-NMR (400 MHz, DMSO-*d*^6^): δ_H_** 10.7 (s, 1 H), 8.7 (s, 1 H), 8.1 (m, 2 H), 7.9 (s, 1 H), 7.5 (m, 3 H), 7.4 (m, 2 H), 7.2 (m, 4 H), 7.1 (m, 1 H), 7.0 (m, 2 H), 3.5 (s, 2 H); **^13^C-NMR (100 MHz DMSO-*d*^6^): δ_C_** 168.0, 140.2, 137.6, 135.7, 133.3, 133.1, 131.5, 130.7, 128.8, 128.5, 128.0, 126.9, 126.4, 125.5, 125.2, 124.4, 123.2, 122.5, 121.1, 120.0, 119.6, 118.6, 111.5, 40.6; **LR-ESI-MS**: C_26_H_18_ClN_2_O [M-H]^-^ *m/z* found 409.05, cald 409.11.

***N*-(2-(9*H*-Carbazol-1-yl)phenyl)-2-(p-tolyl)acetamide (92)**

2-(9*H*-Carbazol-1-yl)aniline (50 mg, 0.194 mmol, 1 eq) and 2-(p-tolyl)acetyl chloride (33 μL, 0.213 mmol, 1.1 eq) were reacted according to general procedure A to give **92** (52 mg, 0.133 mmol 69%) as a white solid.

**v_max_ (cm^-1^)** 3352, 3296, 1582, 1516, 1446, 1236, 739; **^1^H-NMR (400 MHz, DMSO-*d*^6^): δ_H_** 10.7 (s, 1 H), 8.6 (s, 1 H), 8.1 (m, 2 H), 8.0 (d, J=7.95 Hz, 1 H), 7.4 (m, 4 H), 7.3 (m, 1 H), 7.2 (m, 3 H), 6.7 (m, 4 H), 3.4 (s, 2H), 2.2 (s, 3 H); **^13^C-NMR (100 MHz DMSO-*d*^6^): δ_C_** 169.3, 140.2, 137.5, 135.8, 135.4, 131.9, 131.2, 130.7, 128.7, 128.6, 128.0, 126.4, 125.5, 124.9, 123.9, 123.1, 122.6, 120.9, 120.0, 119.5, 118.6, 118.5, 111.4, 42.7; **LR-ESI-MS**: C_27_H_21_N_2_O [M-H]^-^ *m/z* found 389.12, cald 389.17.

***N*-(2-(9*H*-Carbazol-1-yl)phenyl)-2-(4-fluorophenyl)acetamide (93)**

2-(9*H*-Carbazol-1-yl)aniline (50 mg, 0.194 mmol, 1 eq) and 2-(4-fluorophenyl)acetyl chloride (30 μL, 0.213 mmol, 1.1 eq) were reacted according to general procedure A to give **93** (41 mg, 0.104 mmol 54%) as a white solid.

**Mpt:** 191.1-193.1 °C; **v_max_ (cm^-1^)** 3354, 3313, 1664, 1521, 1219, 740; **^19^F-{H}-NMR (376 MHz, DMSO-*d*^6^): δ_F_** -116.0; **^1^H-NMR (400 MHz, DMSO-*d*^6^): δ_H_** 10.7 (s, 1 H), 8.8 (s, 1 H), 8.1 (m, 2 H), 7.9 (d, J=7.95 Hz, 1 H), 7.4 (m, 3 H), 7.4 (m, 2 H), 7.2 (m, 3 H), 6.9 (dd, J=8.50, 5.69 Hz, 2 H), 6.8 (m, 2 H), 3.4 (s, 2 H); **^13^C-NMR (100 MHz DMSO-*d*^6^): δ_C_** 169.1, 162.0, 159.6, 140.2, 137.6, 135.7, 131.8, 131.4 (d, J=2.94 Hz), 130.7, 130.6, 128.0, 126.4, 125.5, 125.3, 124.6, 122.8 (d, J=53.55 Hz), 121.1, 120.0, 119.5, 118.6, 114.7 (d, J=21.27 Hz), 111.4, 41.9; **LR-ESI-MS**: C_26_H_18_FN_2_O [M-H]^-^ *m/z* found 393.07, cald 393.14.

***N*-(2-(9*H*-Carbazol-1-yl)phenyl)-2-(4-methoxyphenyl)acetamide (94)**

2-(9*H*-Carbazol-1-yl)aniline (50 mg, 0.194 mmol, 1 eq) and 2-(4-methoxyphenyl)acetyl chloride (33 μL, 0.213 mmol, 1.1 eq) were reacted according to general procedure A to give **94** (54 mg, 0.133 mmol 69%) as a white solid.

**Mpt:** 191.1-193.1 °C; **v_max_ (cm^-1^)** 3349, 3293, 1657, 1511, 1448, 762; **^1^H-NMR (400 MHz, DMSO-*d*^6^): δ_H_** 10.7 (s, 1 H), 8.6 (s, 1 H), 8.1 (m, 2 H), 8.0 (d, J=7.95 Hz, 1 H), 7.4 (m, 5 H), 7.2 (m, 3 H), 6.8 (d, J=8.68 Hz, 2 H), 6.5 (d, J=8.56 Hz, 2 H), 3.7 (s, 3 H), 3.3 (s, 2H); **^13^C-NMR (100 MHz DMSO-*d*^6^): δ_C_** 169.5, 157.8, 140.2, 137.6, 135.8, 131.3, 130.7, 129.8, 128.0, 126.9, 126.4, 125.5, 125.0, 124.1, 123.2, 122.6, 121.0, 120.0, 119.6, 118.7, 118.6, 113.5, 111.5, 54.9, 42.1; **LR-ESI-MS**: C_27_H_23_N_2_O_2_ [M+H]^+^ *m/z* found 407.49, cald 407.18.

***N*-(2-(9*H*-Carbazol-1-yl)phenyl)-1-(4-cyanophenyl)methanesulfonamide (95)**

2-(9*H*-Carbazol-1-yl)aniline (50 mg, 0.194 mmol, 1 eq) and (4-cyanophenyl)methanesulfonyl chloride (46 mg, 0.213 mmol, 1.1 eq) were reacted according to general procedure A to give **95** (65 mg, 0.149 mmol, 77%) as a white solid.

**v_max_ (cm^-1^)** 3348, 1503, 1415, 1333, 1152, 920, 751; **^1^H-NMR (400 MHz, DMSO-*d*^6^): δ_H_** 10.8 (s, 1 H), 8.8 (s, 1 H), 8.1 (dd, J=7.09, 5.50 Hz, 2 H), 7.7 (d, J=8.31 Hz, 2 H), 7.5 (m, 4 H), 7.4 (m, 3 H), 7.3 (m, 3 H), 7.2 (m, 1 H), 4.1 (s, 2 H); **^13^C-NMR (100 MHz DMSO-*d*^6^): δ_C_** 140.2, 138.0, 135.2, 135.1, 133.9, 131.9, 131.6, 131.3, 128.5, 127.0, 126.1, 125.7, 125.6, 123.0, 122.5, 121.7, 120.1, 119.7, 118.7, 118.5, 111.4, 110.8, 58.1; **LR-ESI-MS**: C_26_H_18_N_3_O_2_S [M-H]^-^ *m/z* found 436.05, cald 436.11.

***N*-(2-(9*H*-Carbazol-1-yl)phenyl)-2-(4-chlorophenyl)acetamide (96)**

2-(9*H*-Carbazol-1-yl)aniline (50 mg, 0.194 mmol, 1 eq) and 2-(4-chlorophenyl)acetyl chloride (40 mg, 0.213 mmol, 1.1 eq) were reacted according to general procedure A to give **96** (37 mg, 0.09 mmol 47%) as a white solid.

**v_max_ (cm^-1^)** 3358, 3316, 1661, 1119, 762; **^1^H-NMR (400 MHz, DMSO-*d*^6^): δ_H_** 10.7 (s, 1 H), 8.8 (s, 1 H), 8.1 (m, 2 H), 7.9 (m, 1 H), 7.4 (m, 3 H), 7.4 (s, 2 H), 7.1 (m, 3 H), 7.0 (m, 2 H), 6.9 (d, J=8.31 Hz, 2 H), 3.4 (s, 2H); **^13^C-NMR (100 MHz DMSO-*d*^6^): δ_C_** 168.8, 140.2 ,137.5, 135.7, 134.3, 131.9, 131.1, 130.7, 130.6, 128.0, 127.9, 126.3, 125.5, 125.3, 124.7, 123.1, 122.6, 121.2, 120.1, 119.5, 118.6, 111.4, 42.0; **LR-ESI-MS**: C_26_H_18_ClN_2_O [M-H]^-^ *m/z* found 409.12, cald 409.11.

***N*-(2-(9*H*-Carbazol-1-yl)phenyl)-1-(4-(trifluoromethyl)phenyl)methanesulfonamide (97)**

2-(9*H*-Carbazol-1-yl)aniline (50 mg, 0.194 mmol, 1 eq) and (4-(trifluoromethyl)phenyl)methanesulfonyl chloride (55 mg, 0.213 mmol, 1.1 eq) were reacted according to general procedure A to give **97** (33 mg, 0.068 mmol 35%) as a white solid.

**v_max_ (cm^-1^)** 3382, 3241, 1417, 1320, 1118, 753; **^19^F-{H}-NMR (376 MHz, DMSO-*d*^6^): δ_F_** -61.2; **^1^H-NMR (400 MHz, DMSO-*d*^6^): δ_H_** 10.8 (s, 1 H), 8.7 (s, 1 H), 8.1 (m, 2 H), 7.5 (m, 6 H), 7.4 (m, 3 H), 7.3 (m, 3 H), 7.2 (m, 1 H), 4.1 (s, 2H); **^13^C-NMR (100 MHz DMSO-*d*^6^): δ_C_** 140.2, 138.0, 135.1, 134.4, 133.7, 131.5, 131.3, 128.5, 128.4, 127.0, 126.0, 125.5, 125.4, 125.0 (m), 123.0, 122.5, 121.6, 120.1, 119.6, 118.6, 118.5, 111.4, 57.9. **LR-ESI-MS**: C_26_H_18_F_3_N_2_O_2_S [M-H]^-^ *m/z* found 479.05, cald 479.10.

***N*-(2-(9*H*-Carbazol-1-yl)phenyl)-2-(3,4-dimethoxyphenyl)acetamide (98)**

2-(9*H*-Carbazol-1-yl)aniline (50 mg, 0.194 mmol, 1 eq) and 2-(3,4-dimethoxyphenyl)acetyl chloride (37 μL, 0.213 mmol, 1.1 eq) were reacted according to general procedure A to give **98** (16 mg, 0.037 mmol 19%) as a white solid.

**v_max_ (cm^-1^)** 3310, 2978, 1671, 1512, 1157, 1122, 545; **^1^H-NMR (400 MHz, DMSO-*d*^6^): δ_H_** 10.7 (m, 1 H), 8.6 (m, 1 H), 8.1 (d, J=7.70 Hz, 1 H), 8.1 (d, J=7.21 Hz, 1 H), 8.0 (d, J=8.31 Hz, 1 H), 7.4 (m, 3 H), 7.4 (m, 1 H), 7.3 (m, 1 H), 7.2 (m, 1 H), 7.1 (m, 2 H), 6.6 (m, 1 H), 6.4 (m, 1 H), 6.3 (dd, J=8.19, 1.96 Hz, 1 H), 3.7 (s, 3 H), 3.5 (s, 3 H); **^13^C-NMR (100 MHz DMSO-*d*^6^): δ_C_** 169.4, 148.4, 147.4, 140.1, 137.5, 135.8, 130.6, 128.0, 127.2, 126.1, 125.5, 123.8, 123.1, 122.5, 120.8, 120.0, 119.6, 118.6, 118.5, 112.7, 111.4, 111.4, 55.3, 55.1, 42.7; **LR-ESI-MS**: C_28_H_23_N_2_O_3_ [M-H]^-^ *m/z* found 435.04, cald 435.17.

***N*-(2-(9*H*-Carbazol-1-yl)phenyl)-2-(benzo[d][1,3]dioxol-5-yl)acetamide (99)**

2-(9*H*-Carbazol-1-yl)aniline (50 mg, 0.194 mmol, 1 eq) and 2-(benzo[d][1,3]dioxol-5-yl)acetyl chloride (38 μL, 0.213 mmol, 1.1 eq) were reacted according to general procedure A to give **99** (54 mg, 0.128 mmol, 66%) as a white solid.

**v_max_ (cm^-1^)** 3359, 3288, 1525, 1582, 1297, 742; **^1^H-NMR (400 MHz, DMSO-*d*^6^): δ_H_** 10.7 (s, 1 H), 8.6 (s, 1 H), 8.1 (t, J=8.07 Hz, 2 H), 8.0 (d, J=7.95 Hz, 1 H), 7.4 (t, J=8.62 Hz, 3 H), 7.3 (m, 2 H), 7.2 (m, 2 H), 7.1 (m, 1 H), 6.5 (s, 1 H), 6.4 (m, 1 H), 6.3 (dd, J=7.89, 1.41 Hz, 1 H), 5.9 (s, 2 H), 3.3 (s, 2 H); **^13^C-NMR (100 MHz DMSO-*d*^6^): δ_C_** 169.2, 146.9, 145.7, 140.2, 137.5, 135.7, 131.2, 130.6, 128.5, 128.0, 126.3, 125.5, 125.0, 123.9, 123.2, 122.6, 121.9, 120.8, 120.0, 119.6, 118.6, 111.4, 109.2, 107.8, 100.7, 42.6; **LR-ESI-MS**: C_27_H_19_N_2_O_3_ [M-H]^-^ *m/z* found 419.15, cald 419.14.

***N*-(2-(9*H*-Carbazol-1-yl)phenyl)-1-(5-cyano-2-fluorophenyl)methanesulfonamide (100)**

2-(9*H*-Carbazol-1-yl)aniline (50 mg, 0.194 mmol, 1 eq) and (5-cyano-2-fluorophenyl)methanesulfonyl chloride (50 mg, 0.213 mmol, 1.1 eq) were reacted according to general procedure A to give **100** (68 mg, 0.149 mmol 77%) as a white solid.

**Mpt:** 204.4-206.4 °C; **v_max_ (cm^-1^)** 3338, 3300, 2239, 1497, 1334, 1152, 1055, 756; **^19^F-{H}-NMR (376 MHz, CDCl_3_): δ_F_** -106.2; **^1^H-NMR (400MHz, CDCl_3_): δ_H_** 8.1 (dd, J=11.98, 7.82 Hz, 2 H), 8.0 (s, 1 H), 7.7 (m, 1 H), 7.5 (m, 6 H), 7.3 (m, 4 H), 7.0 (t, J=8.74 Hz, 1 H), 6.5 (s, 1 H), 4.3 (m, 2 H); **^13^C-NMR (100 MHz CDCl_3_): δ_C_** 139.6, 137.5, 136.5, 136.4, 135.0 (d, J=10.27 Hz), 134.5, 131.3, 129.7, 128.6, 126.6, 126.1, 125.4, 124.4, 123.5, 121.1, 120.7, 120.3, 120.2, 119.3, 119.0, 118.1 (d, J=16.87 Hz), 117.2, 117.0, 111.1, 50.7 (d, J=2.20 Hz); **LR-ESI-MS**: C_26_H_19_FN_3_O_2_S [M+H]^+^ *m/z* found 456.20, cald 456.12.

***N*-(2-(9*H*-Carbazol-1-yl)phenyl)-2-(2,5-dimethylphenyl)acetamide (101)**

2-(9*H*-Carbazol-1-yl)aniline (50 mg, 0.194 mmol, 1 eq) and 2-(2,5-dimethylphenyl)acetyl chloride (35 μL, 0.213 mmol, 1.1 eq) were reacted according to general procedure A to give **101** (52 mg, 0.129 mmol, 66%) as a white solid.

**v_max_ (cm^-1^)** 3336, 1662, 1498, 1446, 1234, 749, 574; **^1^H-NMR (400 MHz, DMSO-*d*^6^): δ_H_** 10.7 (s, 1 H), 8.1 (m, 4 H), 7.4 (m, 2 H), 7.4 (m, 2 H), 7.3 (m, 1 H), 7.2 (m, 3 H), 6.6 (m, 3 H), 3.4 (s, 2 H), 2.0 (s, 3 H), 1.9 (s, 3 H); **^13^C-NMR (100 MHz DMSO-*d*^6^): δ_C_** 168.9, 140.2, 137.3, 135.8, 134.5, 132.9, 132.7, 130.6, 130.3, 129.6, 128.1, 127.4, 126.1, 125.4, 124.7, 123.1, 123.0, 122.7, 120.6, 120.1, 119.8, 118.6, 118.5, 111.4, 41.2, 20.3, 18.3; **LR-ESI-MS**: C_28_H_23_N_2_O [M-H]^-^ *m/z* found 403.16, cald 403.18.

***N*-(2-(9*H*-Carbazol-1-yl)phenyl)-2-(3,4-dichlorophenyl)acetamide (102)**

2-(9*H*-Carbazol-1-yl)aniline (50 mg, 0.194 mmol, 1 eq) and 2-(3,4-dichlorophenyl)acetyl chloride (40 μL, 0.213 mmol, 1.1 eq) were reacted according to general procedure A to give **102** (65 mg, 0.146 mmol 75%) as a white solid.

**v_max_ (cm^-1^)** 3366, 3305, 1670, 1524, 1449, 670, 616; **^1^H-NMR (400 MHz, DMSO-*d*^6^): δ_H_** 10.7 (s, 1 H), 9.0 (s, 1 H), 8.1 (m, 2 H), 7.8 (d, J=7.82 Hz, 1 H), 7.5 (m, 3 H), 7.4 (m, 2 H), 7.3 (d, J=1.10 Hz, 1 H), 7.1 (m, 4 H), 6.9 (dd, J=8.19, 1.71 Hz, 1 H), 3.4 (s, 2 H); **^13^C-NMR (100 MHz DMSO-*d*^6^): δ_C_** 168.4, 140.1, 137.6, 136.5, 135.5, 132.3, 130.9, 130.7, 130.6, 130.1, 129.2, 127.9, 126.2, 125.5 125.2, 123.1, 122.5, 121.2, 120.0, 119.5, 118.6, 118.5, 111.4, 41.5; **LR-ESI-MS**: C_26_H_17_Cl_2_N_2_O [M-H]^-^ *m/z* found 443.05, cald 443.07.

***N*-(2-(9*H*-Carbazol-1-yl)phenyl)-2-(3-methoxyphenyl)acetamide (103)**

2-(9*H*-Carbazol-1-yl)aniline (50 mg, 0.194 mmol, 1 eq) and 2-(3-methoxyphenyl)acetyl chloride (33 μL, 0.213 mmol, 1.1 eq) were reacted according to general procedure A to give **103** (56 mg, 0.138 mmol 71%) as a white solid.

**v_max_ (cm^-1^)** 3343, 3293, 1489, 1464, 1321, 1219, 712, 615; **^1^H-NMR (400 MHz, DMSO-*d*^6^): δ_H_** 10.7 (s, 1 H), 8.8 (s, 1 H), 8.1 (m, 2 H), 7.9 (d, J=8.07 Hz, 1 H), 7.4 (m, 3 H), 7.4 (m, 2 H), 7.1 (m, 3 H), 6.9 (m, 1 H), 6.6 (m, 2 H), 6.5 (d, J=7.58 Hz, 1 H), 3.6 (s, 3 H), 3.4 (s, 2 H); **^13^C-NMR (100 MHz DMSO-*d*^6^): δ_C_** 169.1, 159.0, 140.2, 137.6, 136.6, 135.7, 131.6, 130.6, 129.1, 127.9, 126.2, 125.5, 125.2, 124.6, 123.2, 122.6, 121.0, 120.0, 119.6, 118.6, 114.6, 111.9, 111.4, 54.8, 42.9; **LR-ESI-MS**: C_27_H_23_N_2_O_2_ [M+H]^+^ *m/z* found 407.89, cald 407.18.

***N*-(2-(9*H*-Carbazol-1-yl)phenyl)-2-(3-fluorophenyl)acetamide (104)**

2-(9*H*-Carbazol-1-yl)aniline (50 mg, 0.194 mmol, 1 eq) and 2-(3-fluorophenyl)acetyl chloride (33 μL, 0.213 mmol, 1.1 eq) were reacted according to general procedure A to give **104** (58 mg, 0.147 mmol, 76%) as a white solid.

**v_max_ (cm^-1^)** 3369, 3309, 1581, 1488, 1238, 747; **^19^F-{H}-NMR (376 MHz, DMSO-*d*^6^): δ_F_** -113.6; **^1^H-NMR (400 MHz, DMSO-*d*^6^): δ_H_** 10.7 (s, 1 H), 9.0 (s, 1 H), 8.1 (m, 2 H), 7.8 (d, J=7.82 Hz, 1 H), 7.5 (m, 3 H), 7.4 (m, 2 H), 7.1 (m, 4 H), 6.9 (m, 3 H), 3.4 (s, 2 H); **^13^C-NMR (100 MHz DMSO-*d*^6^): δ_C_** 168.7, 163.1, 160.7, 140.2, 138.1 (d, J=8.07 Hz), 137.6, 135.6, 132.1, 130.7, 129.9 (d, J=8.07 Hz), 127.9, 126.3, 125.5, 125.4, 125.2, 125.0 (d, J= 2.2 Hz), 123.2, 122.6, 121.2, 120.0, 119.5, 118.6 (d, J=8.07 Hz), 115.8 (d, 21.3 Hz), 113.0 (d, J= 21.3 Hz), 111.4, 42.3; **LR-ESI-MS**: C_26_H_18_FN_2_O [M-H]^-^ *m/z* found 393.07, cald 393.14.

***N*-(2-(9*H*-Carbazol-1-yl)phenyl)-1-(3-fluorophenyl)methanesulfonamide (105)**

2-(9*H*-Carbazol-1-yl)aniline (50 mg, 0.194 mmol, 1 eq) and (3-fluorophenyl)methanesulfonyl chloride (44 mg, 0.213 mmol, 1.1 eq) were reacted according to general procedure A to give **105** (62 mg, 0.144 mmol 74%) as a white solid.

**Mpt:** 75.9-77.9 °C; **v_max_ (cm^-1^)** 3336, 2923, 2848, 1588, 1483, 1318, 1150, 921, 750, 500; **^19^F-{H}-NMR (376 MHz, DMSO-*d*^6^): δ_F_** -113.5; **^1^H-NMR (400 MHz, DMSO-*d*^6^): δ_H_** 10.8 (m, 1 H), 8.7 (m, 1 H), 8.1 (t, J=6.72 Hz, 2 H), 7.5 (m, 4 H), 7.3 (s, 3 H), 7.3 (s, 2 H), 7.1 (m, 2 H), 6.9 (s, 2 H), 4.0 (s, 2 H); **^13^C-NMR (100 MHz DMSO-*d*^6^): δ_C_** 162.9, 160.5, 140.2, 138.0, 135.3, 133.8, 132.1 (d, J=8.07 Hz), 131.3, 130.1 (d, J=8.80 Hz), 128.5, 127.0, 126.9 (d, J=2.93 Hz), 126.0, 125.6, 123.0, 122.5, 121.8, 120.1, 119.5, 119.6, 118.6 (d, J=12.47 Hz), 117.4 (d, J=22.01 Hz), 115.0 (d, J=20.54 Hz), 111.4, 57.9; **LR-ESI-MS**: C_25_H_18_FN_2_O_2_S [M-H]^+^ *m/z* found 429.11, cald 429.11.

***N*-(2-(9*H*-Carbazol-1-yl)phenyl)-2-(3-(trifluoromethyl)phenyl)acetamide (106)**

2-(9*H*-Carbazol-1-yl)aniline (50 mg, 0.194 mmol, 1 eq) and 2-(3-(trifluoromethyl)phenyl)acetyl chloride (43 μL, 0.213 mmol, 1.1 eq) were reacted according to general procedure A to give **106** (58 mg, 0.130 mmol, 67%) as a white solid.

**v_max_ (cm^-1^)** 3361, 3312, 2972, 1666, 1525, 1449, 1333, 1127, 584; **^19^F-{H}-NMR (376 MHz, DMSO-*d*^6^): δ_F_** -60.9; **^1^H-NMR (400 MHz, DMSO-*d*^6^): δ_H_** 10.7 (s, 1 H), 9.1 (s, 1 H), 8.1 (m, 2 H), 7.8 (s, 1 H), 7.5 (m, 5 H), 7.4 (m, 2 H), 7.3 (s, 1 H), 7.2 (m, 4 H), 3.5 (s, 2 H); **^13^C-NMR (100 MHz DMSO-*d*^6^): δ_C_** 168.7, 140.1, 137.6, 137.0, 135.6, 133.0, 132.4, 130.7, 129.0, 128.9, 128.7, 127.9, 126.2, 125.6 (m), 125.5, 123.2, 123.1, 123.0, 122.5, 121.3, 120.0, 119.5, 118.6, 118.4, 111.4, 42.1; **LR-ESI-MS**: C_27_H_18_F_3_N_2_O [M-H]^-^ *m/z* found 443.12, cald 443.14.

***N*-(2-(9*H*-Carbazol-1-yl)phenyl)-2-(3-cyanophenyl)acetamide (107)**

2-(9*H*-Carbazol-1-yl)aniline (50 mg, 0.194 mmol, 1 eq), 2-(3-cyanophenyl)acetic acid (37 mg, 0.232 mmol, 1.2 eq) and DIPEA (81 μL, 0.465 mmol, 2.4 eq) were dissolved in DCM (2 mL, 0.1 M) and then had HATU (88 mg, 0.232 mmol, 1.2 eq) added before the solution was stirred for 16 h at room temperature. Upon reaction completion the crude mixture was concentrated onto silica gel before being purified by Isolera Biotage LPLC (CH/EA 8:2) to give **107** (56 mg, 0.139 mmol, 72%) as a white solid.

**v_max_ (cm^-1^)** 3359, 3305, 1523, 1313, 1237, 746; **^1^H-NMR (400 MHz, CDCl_3_): δ_H_** 8.3 (d, J=8.19 Hz, 1 H), 8.1 (t, J=8.68 Hz, 2 H), 7.7 (s, 1 H), 7.5 (m, 2 H), 7.3 (m, 5 H), 7.2 (dd, J=7.34, 0.98 Hz, 1 H), 7.1 (d, J=7.70 Hz, 1 H), 7.0 (s, 1 H), 6.8 (m, 2 H), 6.7 (m, 1 H), 3.4 (s, 2 H); **^13^C-NMR (100 MHz CDCl_3_): δ_C_** 168.0, 139.4, 137.2, 134.9, 134.6, 132.9, 131.9, 130.9, 130.6, 129.3, 129.2, 126.5, 126.4, 125.3, 123.5, 123.3, 121.7, 120.7, 120.1, 119.6, 118.0, 112.7, 110.8, 44.1; **LR-ESI-MS**: C_27_H_18_N_3_O [M-H]^-^ *m/z* found 400.19, cald 400.15.

***N*-(2-(9*H*-Carbazol-1-yl)phenyl)-1-(3-cyanophenyl)methanesulfonamide (108)**

2-(9*H*-Carbazol-1-yl)aniline (50 mg, 0.194 mmol, 1 eq) and (3-cyanophenyl)methanesulfonyl chloride (46 mg, 0.213 mmol, 1.1 eq) were reacted according to general procedure A to give **115** (62 mg, 0.142 mmol 73%) as a white solid.

**Mpt:** 88.0-90.0 °C; **v_max_ (cm^-1^)** 3334, 2231, 1454, 1150, 920, 752, 685; **^1^H-NMR (400MHz, DMSO-*d*^6^): δ_H_** 10.8 (m, 1 H), 8.8 (s, 1 H), 8.1 (t, J=7.46 Hz, 2 H), 7.7 (m, 1 H), 7.5 (m, 5 H), 7.4 (m, 5 H), 7.3 (m, J=7.58 Hz, 1 H), 7.2 (t, J=7.46 Hz, 1 H), 4.1 (s, 2 H); **^13^C-NMR (100 MHz DMSO-*d*^6^): δ_C_** 140.2, 138.0, 135.6, 135.1, 134.1, 134.0, 131.8, 131.4, 129.5, 128.5, 127.1, 126.2, 125.9, 125.6, 123.0, 122.5, 121.8, 120.1, 119.7, 118.7, 118.5, 118.4, 111.5, 111.3, 57.6; **LR-ESI-MS**: C_26_H_18_N_3_O_2_S [M+H]^+^ *m/z* found 436.09, cald 436.11.

***N*-(2-(9*H*-Carbazol-1-yl)phenyl)-1-(m-tolyl)methanesulfonamide (109)**

2-(9*H*-Carbazol-1-yl)aniline (50 mg, 0.194 mmol, 1 eq) and m-tolylmethanesulfonyl chloride (79 mg, 0.387 mmol, 2 eq) were reacted according to general procedure A to give *N*-(2-(9H-carbazol-1-yl)phenyl)-1-(m-tolyl)methanesulfonamide (14 mg, 0.033 mmol 17%) as a white solid.

**v_max_ (cm^-1^)** 3704, 2972, 1600, 1389, 1318, 1149, 1054, 1014, 919, 636; **^1^H-NMR (400 MHz, DMSO-*d*^6^): δ_H_** 10.8 (s, 1 H), 8.6 (s, 1 H), 8.1 (t, J=6.66 Hz, 2 H), 7.5 (m, 4 H), 7.4 (m, 3 H), 7.3 (m, 1 H), 7.2 (m, 1 H), 7.1 (m, 2 H), 6.8 (d, J=6.97 Hz, 1 H), 6.8 (s, 1 H), 3.9 (br. s., 2 H), 2.1 (s, 3 H); **^13^C-NMR (100 MHz DMSO-*d*^6^): δ_C_** 140.2, 138.0, 137.3, 135.4, 133.7, 131.3, 131.2, 129.2, 128.6, 128.1, 127.8, 126.9, 125.9, 125.5, 123.0, 122.5, 121.8, 120.1, 119.7, 118.6, 118.5, 111.4, 58.3, 20.7; **LR-ESI-MS**: C_26_H_23_N_2_O_2_S [M+H]^+^ *m/z* found 427.39, cald 427.15.

***N*-(2-(9*H*-Carbazol-1-yl)phenyl)-3-cyanobenzenesulfonamide (110)**

2-(9*H*-Carbazol-1-yl)aniline (50 mg, 0.194 mmol, 1 eq) and 3-cyanobenzenesulfonyl chloride (43 mg, 0.213 mmol, 1.1 eq) were reacted according to general procedure A to give *N*-(2-(9H-carbazol-1-yl)phenyl)-3-cyanobenzenesulfonamide (4.1 mg, 0.0097 mmol 5%) as a white solid.

**v_max_ (cm^-1^)** 3411, 3229, 1417, 1314, 1153, 752; **^1^H-NMR (400 MHz, DMSO-*d*^6^): δ_H_** 10.5 (s, 1 H), 9.8 (s, 1 H), 8.1 (d, J=7.82 Hz, 1 H), 8.0 (dd, J=6.91, 1.90 Hz, 1 H), 7.4 (m, 8 H), 7.2 (m, 5 H); **^13^C-NMR (100 MHz DMSO-*d*^6^): δ_C_** 141.1, 140.0, 137.7, 135.9, 134.8, 133.1, 131.3, 129.9, 129.5, 129.1, 128.9, 128.4, 127.6, 126.6, 125.4, 122.9, 122.4, 120.9, 119.9, 119.6, 118.5, 118.0, 117.3, 111.6, 111.4; **LR-ESI-MS**: C_25_H_16_N_3_O_2_S [M-H]^-^ *m/z* found 422.16, cald 422.09.

***N*-(2-(9*H*-Carbazol-1-yl)phenyl)-1-(4-fluorophenyl)methanesulfonamide (111)**

2-(9*H*-Carbazol-1-yl)aniline (50 mg, 0.194 mmol, 1 eq) and (4-fluorophenyl)methanesulfonyl chloride (44 mg, 0.213 mmol, 1.1 eq) were reacted according to general procedure A to give *N*-(2-(9H-carbazol-1-yl)phenyl)-1-(4-fluorophenyl)methanesulfonamide (23 mg, 0.053 mmol 28%) as a white solid.

**v_max_ (cm^-1^)** 3351, 1601, 1505, 1223, 1149, 751; **^19^F-{H}-NMR (376 MHz, DMSO-*d*^6^): δ_F_** -114.0; **^1^H-NMR (400 MHz, DMSO-*d*^6^): δ_H_** 10.8 (s, 1 H), 8.5 (s, 1 H), 8.1 (m, 2 H), 7.5 (m, 4 H), 7.4 (m, 3 H), 7.3 (m, 1 H), 7.2 (m, 1 H), 7.1 (m, 2 H), 7.0 (m, 2 H), 4.0 (s, 2 H); **^13^C-NMR (100 MHz DMSO-*d*^6^): δ_C_** 163.2, 160.7, 140.2, 138.0, 135.2, 133.5, 132.7 (d, J=8.80 Hz), 131.3, 128.5, 127.0, 125.8, 125.5, 125.3, 123.0, 122.5, 121.7, 120.1, 119.6, 118.6, 118.5, 115.1, 114.9, 111.4, 57.4; **LR-ESI-MS**: C_25_H_18_FN_2_O_2_S [M-H]^-^ *m/z* found 429.07, cald 429.11.

***N*-(2-(9*H*-Carbazol-1-yl)phenyl)-1-phenylcyclopropane-1-carboxamide (112)**

2-(9*H*-Carbazol-1-yl)aniline (25 mg, 0.097 mmol, 1 eq), 1-phenylcyclopropane-1-carboxylic acid (19 mg, 0.116 mmol, 1.2 eq) and DIPEA (81 μL, 0.465 mmol, 4.8 eq) were dissolved in DCM (1 mL, 0.1 M) and then had HATU (44 mg, 0.116 mmol, 1.2 eq) added before the solution was stirred for 16 h at room temperature. Upon reaction completion the crude mixture was concentrated onto silica gel before being purified by HPLC to give *N*-(2-(9H-carbazol-1-yl)phenyl)-1-phenylcyclopropane-1-carboxamide (2.4 mg, 0.006 mmol, 6%) as a white solid.

**v_max_ (cm^-1^)** 3366, 3303, 1659, 1519, 1319, 1306, 697; **^1^H-NMR (400 MHz, DMSO-*d*^6^): δ_H_** 10.6 (s, 1 H), 8.4 (d, J=7.95 Hz, 1 H), 8.2 (d, J=7.83 Hz, 1 H), 8.1 (d, J=6.97 Hz, 1 H), 7.4 (m, 4 H), 7.2 (m, 5 H), 7.1 (d, J=15.04 Hz, 1 H), 7.0 (dd, J=7.27, 1.04 Hz, 1 H), 6.7 (m, 1 H), 6.6 (m, 2 H), 6.5 (m, 2 H), 1.3 (m, 2 H), 0.8 (d, J=3.30 Hz, 2 H); **^13^C-NMR (100 MHz DMSO-*d*^6^): δ_C_** 171.1, 140.3, 137.4, 136.8, 136.0, 130.6, 129.4, 128.7, 128.3, 128.0, 127.2, 126.1, 125.6, 124.0, 123.1, 122.9, 120.2, 120.0, 118.9, 118.7, 111.5, 30.9, 15.4; **LR-ESI-MS**: C_28_H_23_N_2_O [M+H]^+^ *m/z* found 403.64, cald 403.18.

***N*-(2-(9*H*-Carbazol-1-yl)phenyl)-2-(4-methylpiperazin-1-yl)acetamide (113)**

2-(9*H*-Carbazol-1-yl)aniline (50 mg, 0.194 mmol, 1 eq), 2-(4-methylpiperazin-1-yl)acetic acid (37 mg, 0.232 mmol, 1.2 eq) and DIPEA (68 μL, 0.387 mmol, 2 eq) were dissolved in DCM (2 mL, 0.1 M) and then had HATU (88 mg, 0.232 mmol, 1.2 eq) added before the solution was stirred for 16 h at room temperature. Upon reaction completion the crude mixture was concentrated onto silica gel before being purified by HPLC to give *N*-(2-(9H-carbazol-1-yl)phenyl)-2-(4-methylpiperazin-1-yl)acetamide (17.2 mg, 0.043 mmol, 22%) as a white solid.

**Mpt:** 223.8-225.8 °C; **v_max_ (cm^-1^)** 3290, 3206, 1671, 1521, 1449, 829, 747, 556; **^1^H-NMR (400 MHz, DMSO-*d*^6^): δ_H_** 10.9 (s, 1 H), 9.2 (s, 1 H), 8.5 (d, J=8.31 Hz, 1 H), 8.2 (m, 2 H), 7.5 (m, 2 H), 7.3 (m, 5 H), 7.2 (m, 1 H), 3.6 (m, 1 H), 2.8 (s, 2 H), 2.0 (m, 7 H), 1.2 (m, 6 H); **^13^C-NMR (100 MHz DMSO-*d*^6^): δ_C_** 167.9, 140.4, 137.5, 135.8, 130.8, 128.5, 128.2, 126.7, 125.8, 123.8, 123.3, 122.6, 120.6, 120.3, 119.5, 119.2, 118.9, 111.4, 61.0, 53.0, 51.6; **LR-ESI-MS**: C_25_H_27_N_4_O [M+H]^+^ *m/z* found 399.64, cald 399.22.

***N*-(2-(9*H*-Carbazol-1-yl)phenyl)-3-(4-methylpiperazin-1-yl)propanamide** **(114)**

2-(9*H*-Carbazol-1-yl)aniline (50 mg, 0.194 mmol, 1 eq), 3-(4-methylpiperazin-1-yl)propanoic acid.2HCl (57 mg, 0.232 mmol, 1.2 eq) and DIPEA (135 μL, 0.774 mmol, 4 eq) were dissolved in DCM (2 mL, 0.1 M) and then had HATU (88 mg, 0.232 mmol, 1.2 eq) added before the solution was stirred for 16 h at room temperature. Upon reaction completion the crude mixture was concentrated onto silica gel before being purified by Isolera Biotage LPLC (DCM/MeOH 9:1) to give *N*-(2-(9H-carbazol-1-yl)phenyl)-3-(4-methylpiperazin-1-yl)propanamide (61 mg, 0.147 mmol, 76%) as a white solid.

**v_max_ (cm^-1^)** 3271, 2796, 1662, 1535, 1242, 737, 649; **^1^H-NMR (400 MHz, DMSO-*d*^6^): δ_H_** 10.8 (s, 1 H), 9.5 (s, 1 H), 8.1 (m, 2 H), 8.0 (d, J=8.19 Hz, 1 H), 7.5 (m, 3 H), 7.3 (m, 2 H), 7.2 (m, 2 H), 7.1 (m, 1 H), 2.2 (m, 2 H), 2.1 (br. s., 2 H), 1.8 (m, 10 H); **^13^C-NMR (100 MHz DMSO-*d*^6^): δ_C_** 170.2, 140.2, 137.8, 135.8, 131.4, 130.8, 127.9, 126.5, 125.6, 123.4, 122.5, 121.6, 120.0, 119.6, 118.7, 118.6, 111.4, 54.0, 52.9, 51.6, 45.1, 32.9; **LR-ESI-MS**: C_26_H_29_N_4_O [M+H]^+^ *m/z* found 413.60, cald 413.23.

***N*-(2-(9*H*-Ccarbazol-1-yl)phenyl)-4-(dimethylamino)butane-1-sulfonamide (115)**

2-(9*H*-Carbazol-1-yl)aniline (50 mg, 0.194 mmol, 1 eq), 4-(dimethylamino)butane-1-sulfonyl chloride.HCl (55 mg, 0.232 mmol, 1.2 eq) and DIPEA (68 μL, 0.387 mmol, 2 eq) were dissolved in DCM (2 mL, 0.1 M) and then had HATU (88 mg, 0.232 mmol, 1.2 eq) added before the solution was stirred for 16 h at room temperature. Upon reaction completion the crude mixture was concentrated onto silica gel before being purified by Isolera Biotage LPLC (DCM/MeOH 9:1) to give *N*-(2-(9H-carbazol-1-yl)phenyl)-4-(dimethylamino)butane-1-sulfonamide (55 mg, 0.131 mmol, 68%) as a white solid.

**v_max_ (cm^-1^)** 3290, 1584, 1252, 1136, 978, 750, 616; **^1^H-NMR (400 MHz, DMSO-*d*^6^): δ_H_** 10.7 (m, 1 H), 8.1 (t, J=7.52 Hz, 2 H), 7.5 (s, 1 H), 7.5 (m, 4 H), 7.3 (d, J=7.58 Hz, 2 H), 7.3 (d, J=7.58 Hz, 1 H), 7.2 (s, 1 H), 1.9 (s, 7 H), 1.7 (s, 2 H), 1.1 (m, 2 H), 0.8 (m, 2 H); **^13^C-NMR (100 MHz DMSO-*d*^6^): δ_C_** 146.6, 140.2, 138.8, 131.7, 127.7, 126.8, 126.5, 125.2, 123.9, 122.8, 122.4, 122.2, 119.9, 118.6, 118.5, 118.4, 118.2, 111.4, 83.7, 67.5, 67.2, 52.0, 45.4, 24.2, 17.9; **LR-ESI-MS**: C_24_H_28_N_3_O_2_S [M+H]^+^ *m/z* found 422.41, cald 422.19.

***N*-(2-(9*H*-Carbazol-1-yl)phenyl)-2-(3-cyanophenyl)propanamide (116)**

2-(9*H*-Carbazol-1-yl)aniline (50 mg, 0.194 mmol, 1 eq), 2-(3-cyanophenyl)propanoic acid (41 mg, 0.232 mmol, 1.2 eq) and DIPEA (101 μL, 0.581 mmol, 3 eq) were dissolved in DCM (2 mL, 0.1 M) and then had HATU (88 mg, 0.232 mmol, 1.2 eq) added before the solution was stirred for 16 h at room temperature. Upon reaction completion the crude mixture was concentrated onto silica gel before being purified by HPLC to give *N*-(2-(9H-carbazol-1-yl)phenyl)-2-(3-cyanophenyl)propanamide (10 mg, 0.024 mmol, 12%) as a white solid.

**v_max_ (cm^-1^)** 3342, 3256, 2225, 1651, 1036, 747; **^1^H-NMR (400 MHz, DMSO-*d*^6^): δ_H_** 10.7 (s, 1 H), 9.0 (s, 1 H), 8.1 (m, 2 H), 7.8 (d, J=7.83 Hz, 1 H), 7.5 (m, 5 H), 7.3 (m, 4 H), 7.2 (m, 1 H), 7.0 (d, J=6.85 Hz, 2 H), 3.7 (m, 1 H), 1.2 (d, J=6.97 Hz, 3 H); **^13^C-NMR (100 MHz DMSO-*d*^6^): δ_C_** 171.5, 142.7, 140.1, 137.5, 135.4, 132.6, 132.0, 130.8, 130.7, 130.3, 129.3, 127.9, 126.2, 125.6, 125.5, 123.1, 122.5, 121.3, 120.0, 119.5, 118.7, 118.6, 118.4, 111.4, 111.2, 44.7, 17.9; **LR-ESI-MS**: C_28_H_23_N_2_O [M+H]^+^ *m/z* found 416.46, cald 416.18.

**ITC Data**


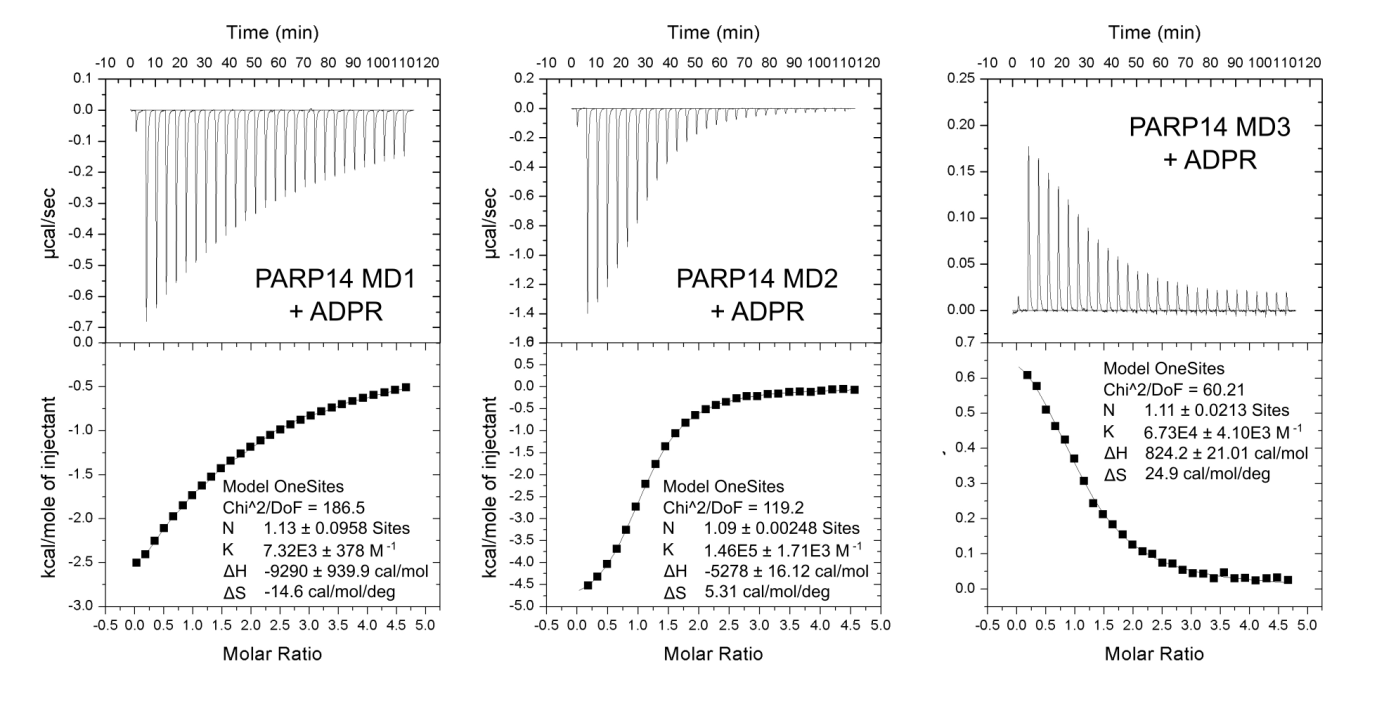


**Supplemental Figure 1.** Isothermal Titration Calorimetry curves of ADPR titrated into PARP14 MD1, MD2 and MD3.

**NMR Spectra of Representative Compounds**

**^1^H NMR Spectra**


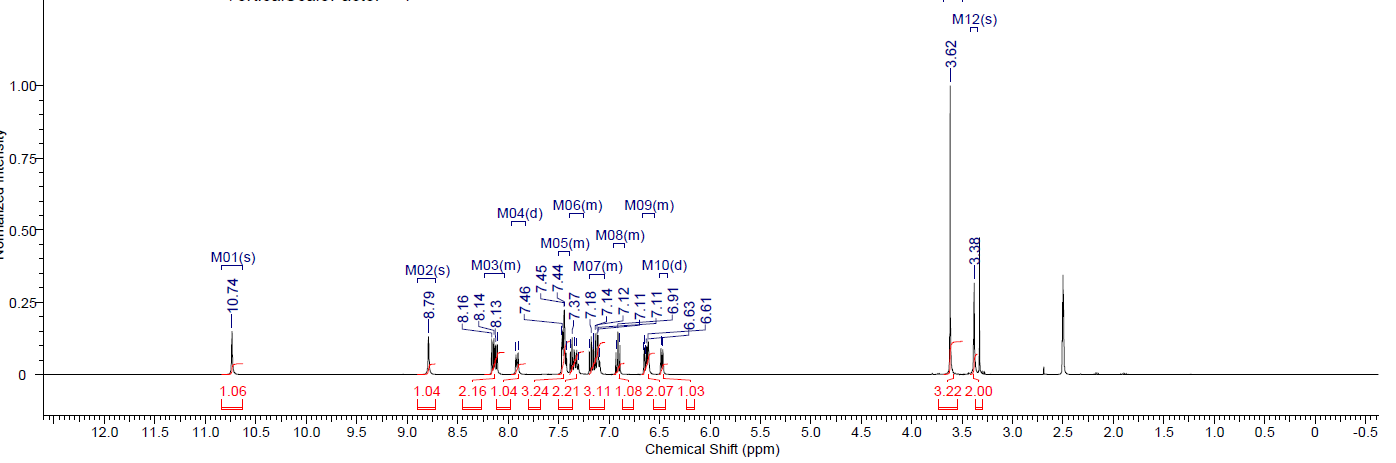


Supplementary Figure 1 ^1^H NMR of compound 103


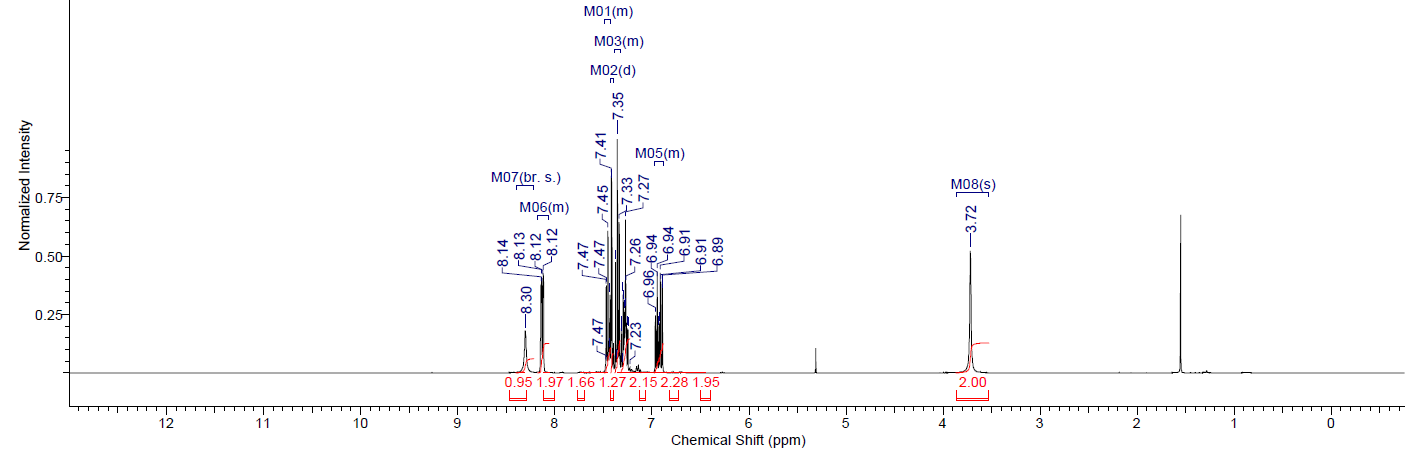


Supplementary Figure 2 ^1^H NMR of compound 48


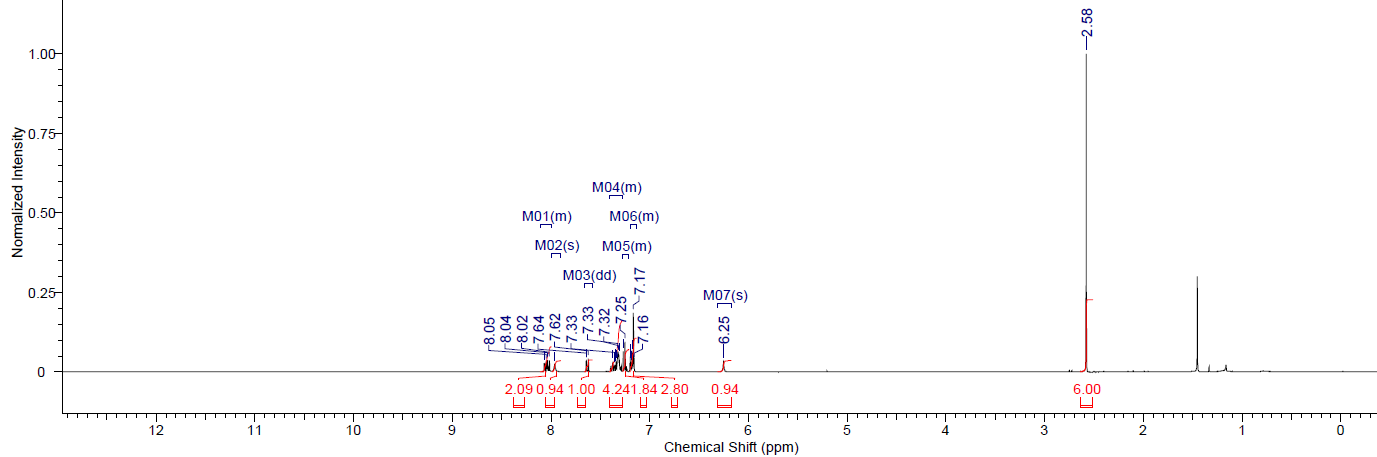


Supplementary Figure 3 ^1^H NMR of compound 71


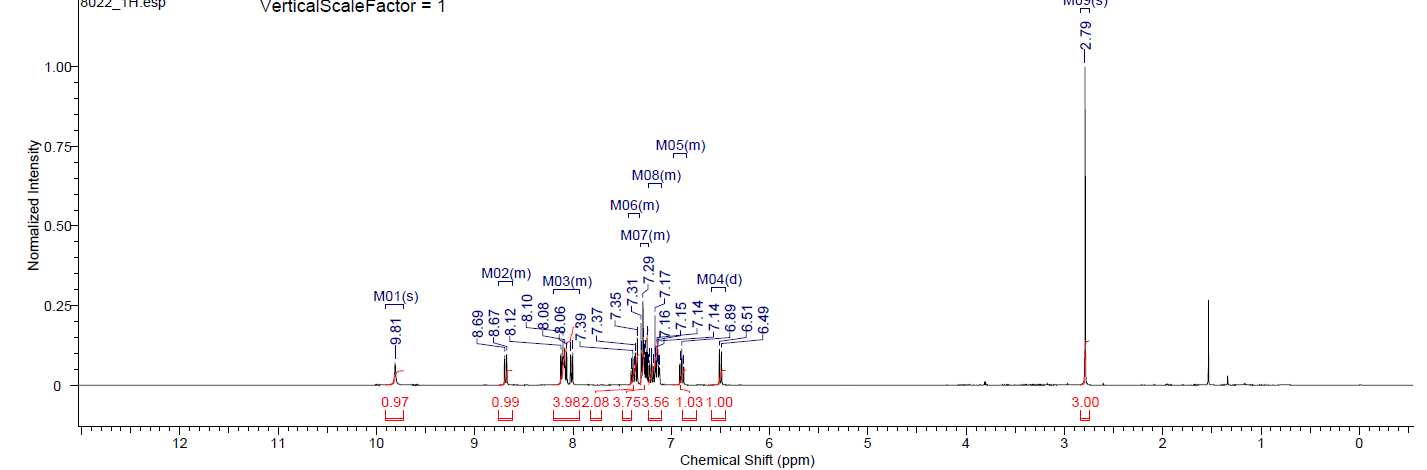


Supplementary Figure 4 ^1^H NMR of compound 80


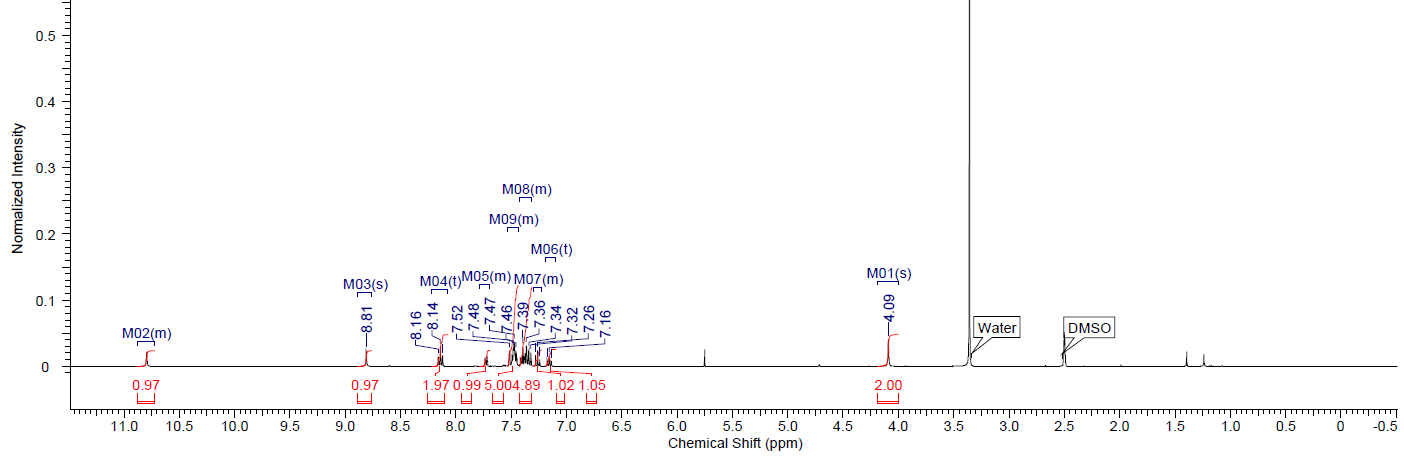


Supplementary Figure 5 ^1^H NMR of compound 108

**^13^C NMR Spectra**


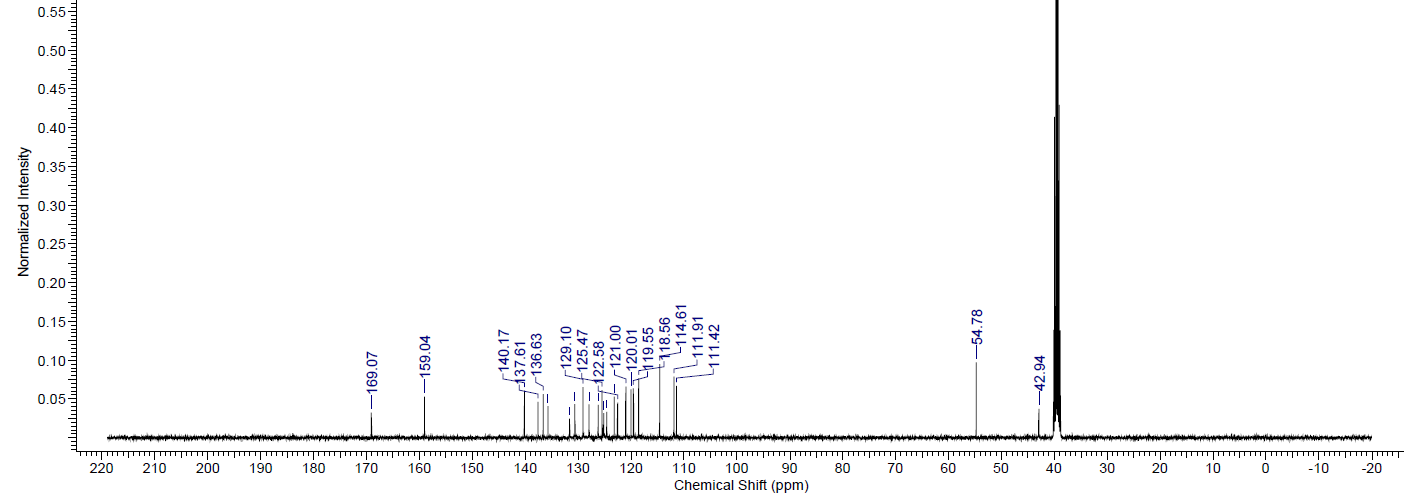


Supplementary Figure 6 ^13^C NMR of compound 103


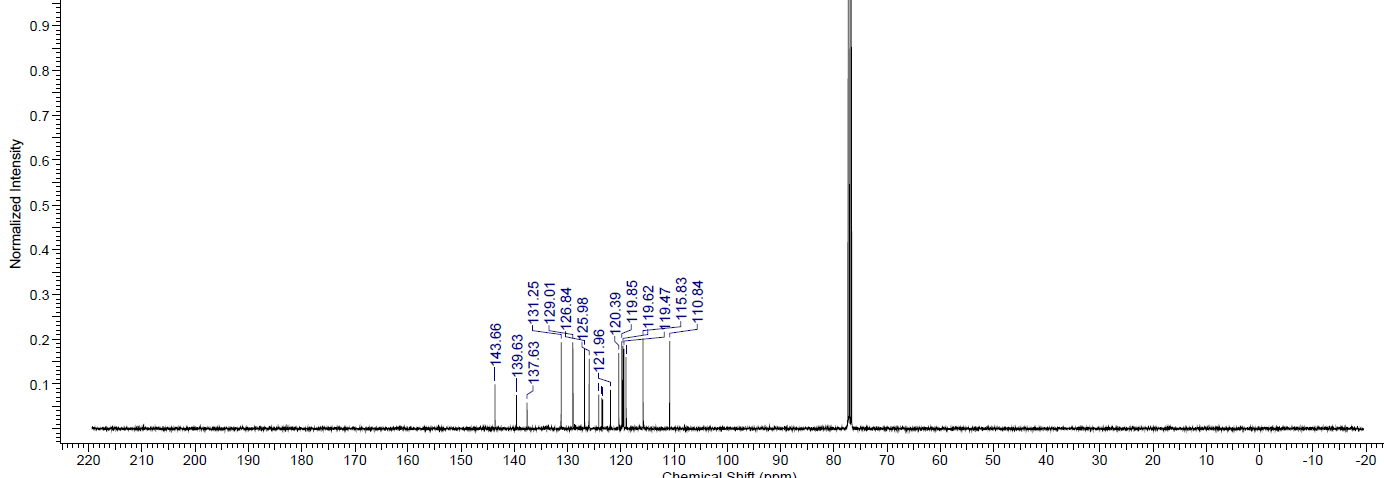


Supplementary Figure 7 ^13^C NMR of compound 48


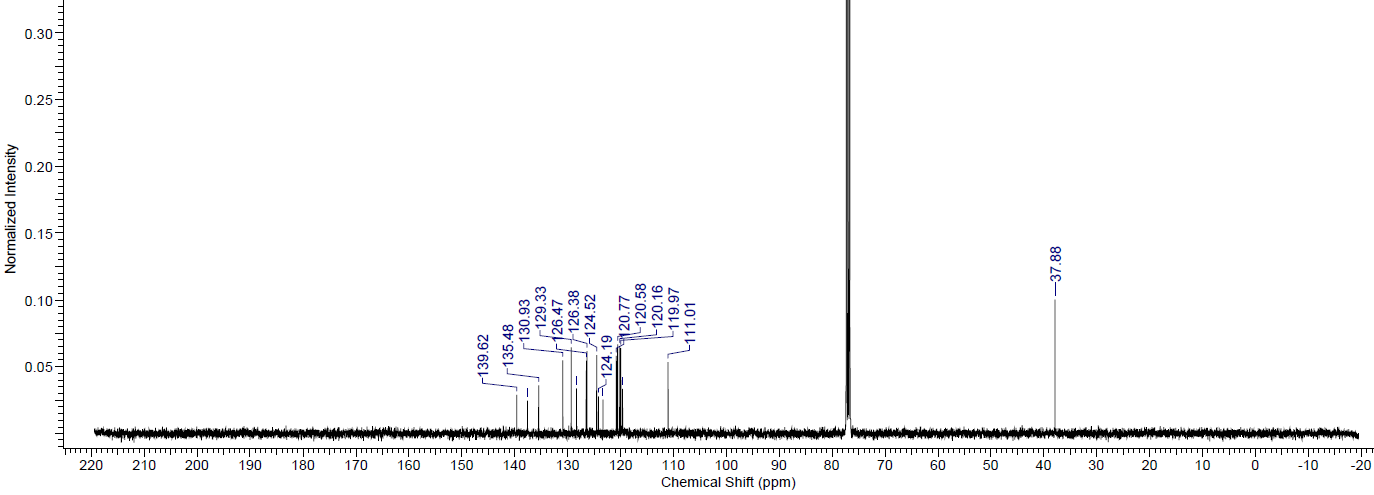


Supplementary Figure 8 ^13^C NMR of compound 71


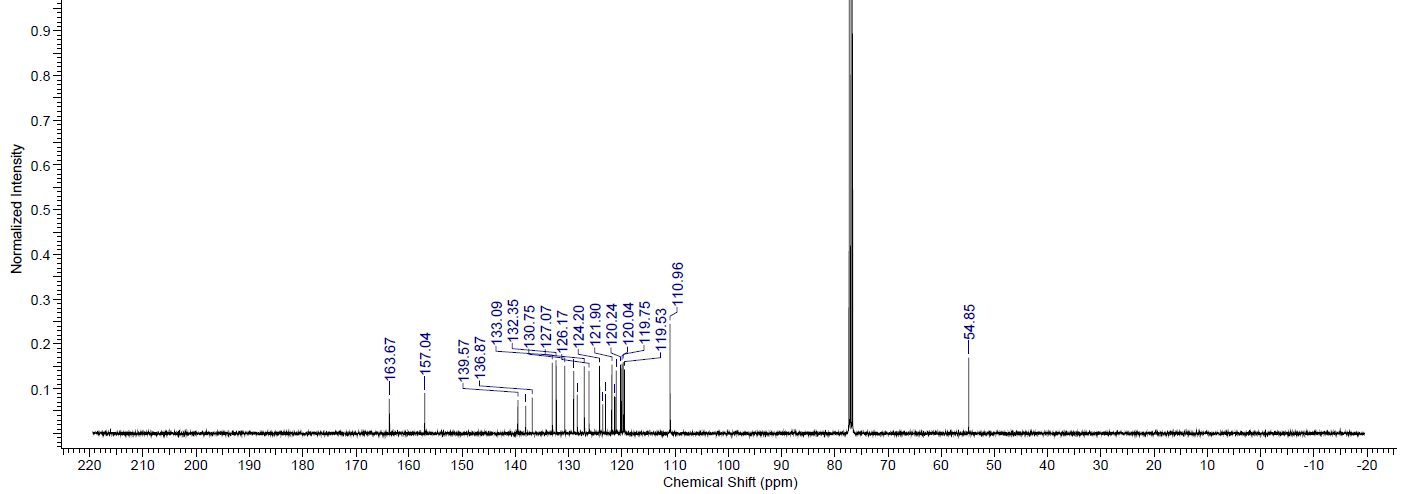


Supplementary Figure 9 ^13^C NMR of compound 80


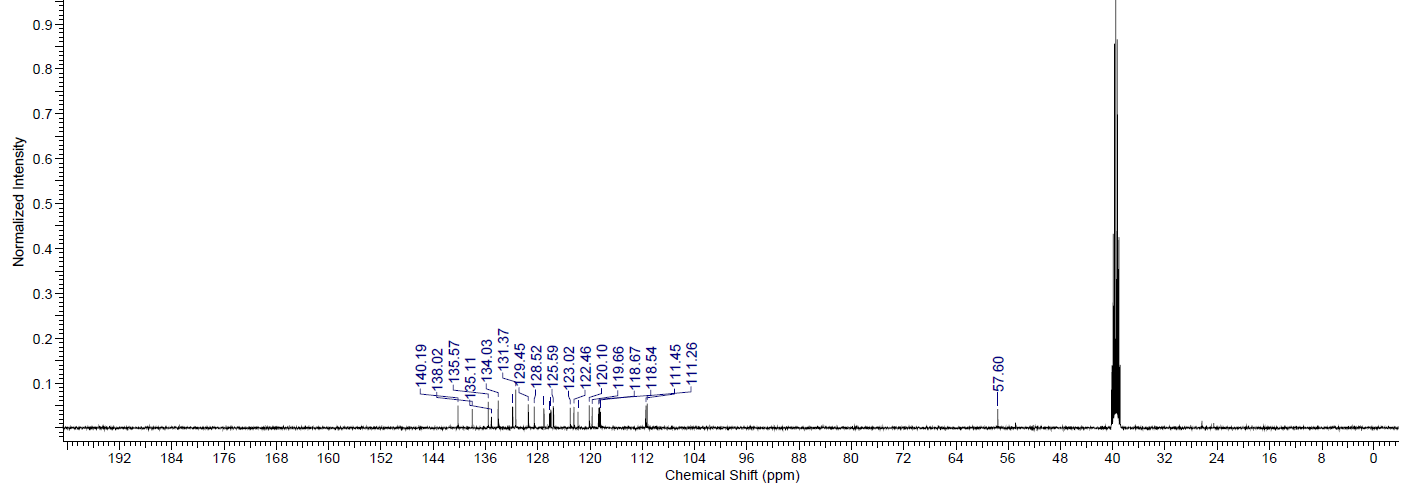


Supplementary Figure 10 ^13^C NMR of compound 108
